# Supplementary material for: The CTLA‐4 immune checkpoint protein regulates PD‐L1:PD‐1 interaction via transendocytosis of its ligand CD80
Source: EMBO J. 2023 Feb 2;42(5):e111556. doi: 10.15252/embj.2022111556 (PMC9975936; doi:10.15252/embj.2022111556)
Supplement: Supplementary file 1 — Expanded View Figures PDF [file EMBJ-42-e111556-s001.pdf]

Expanded View Figures

Figure EV1. Impact of co-expression of CD80, CD86 and PD-L1 on detection.

A CHO cells expressing PD-L1 alone or in combination with CD80 or CD86 were stained using the reagents shown and quantified by flow cytometry. The impact of CD80 and CD86 on detection of PD-L1 using PD-1 Ig (Left panel) or CTLA-4 Ig (abatacept- Right panel) is shown. Note the low binding of CTLA-4-Ig to CD86 is due to natural affinity differences (mean ± SD, three independent experiments). *$P \leq 0.05$, **$P \leq 0.01$, ****$P \leq 0.0001$, ns, not significant: RM one-way ANOVA.

B Concatenated flow cytometry plots (left panel) for Durvalumab-APC binding to DG-75 cells expressing PD-L1 or PD-L1/CD80, with dose response from three independent experiments showing mean ± SEM (middle panel). EC50 values of Durvalumab binding are plotted (right hand panel). **$P \leq 0.01$: paired *t*-test.

C Down regulation of Jurkat CD28 following engagement by CD80 or CD86 in the presence or absence of PD-L1 is shown as a measure of CD28 interaction with ligands. CD28 MFI was determined using flow cytometry is quantified in right hand panel (mean ± SEM, three independent experiments, ****$P \leq 0.0001$: two-way ANOVA with Tukey's multiple comparisons test).

© 2023 The Authors

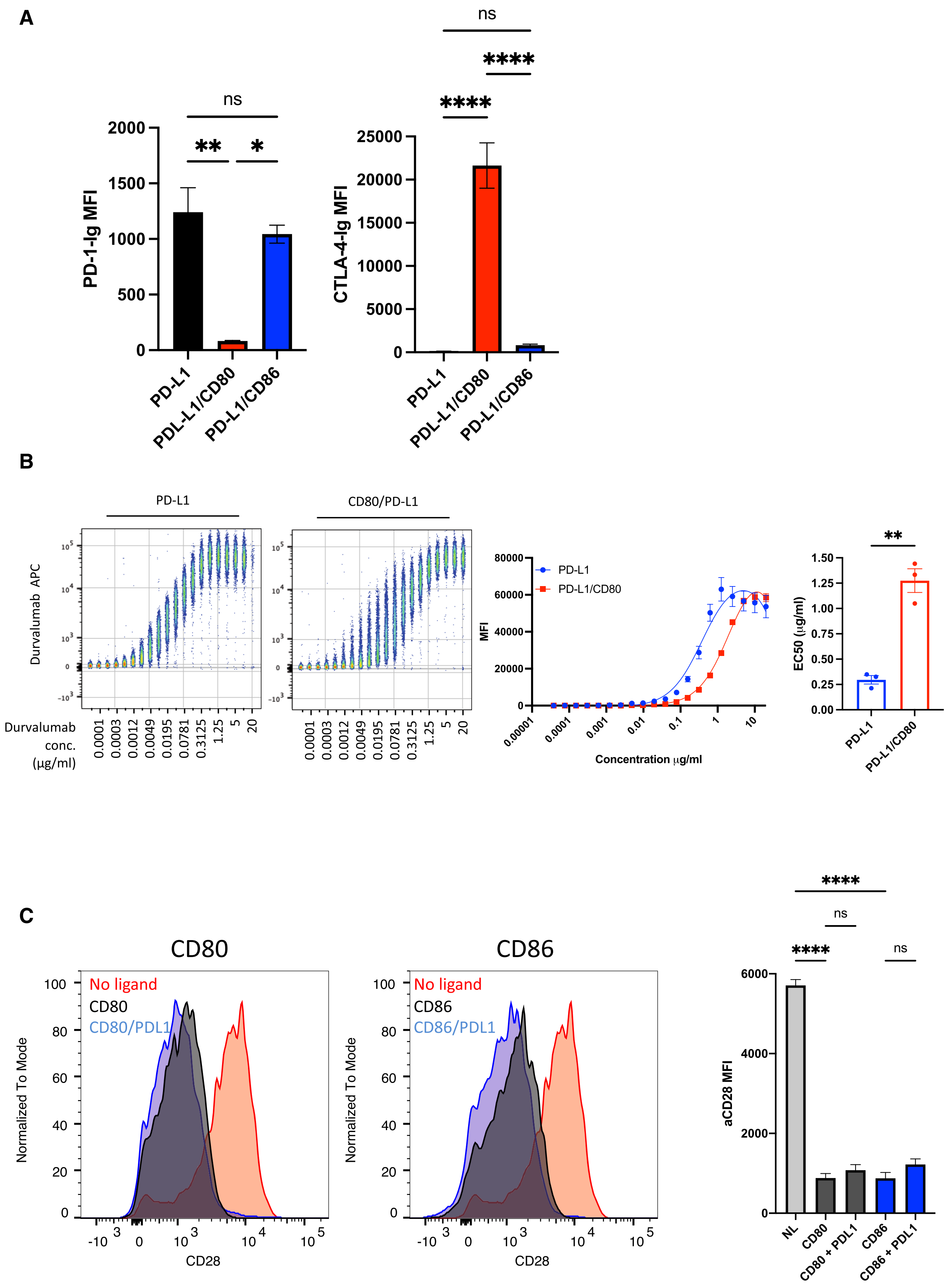

Figure EV1.

 © 2023 The Authors

Figure EV2. Measurement of transendocytosis by flow cytometry.

A Cartoon showing the principle of transendocytosis assays by flow cytometry (licenced under CC-BY from ref.22). Ligand (donor) cells expressing CD80 or CD86 proteins with GFP fusion tags (green plasma membrane) are labelled with CellTrace Violet (CTV^+, purple) and mixed with CTLA-4 expressing recipient cells (orange dots and membrane, CTV^-). During transendocytosis, plasma membrane expressed ligands are removed from donor cells (reduced green plasma membrane signal) and fluorescent ligand is now detected in CTLA-4 expressing recipient cells. Internalised ligands either separate from CTLA-4 (green dots) or remain colocalised (orange dots with green outline). Right hand panel—FACS plot shows representative result of TE, where ligand removal results in loss of GFP fluorescence from the CTV^+ donor cell, and a concomitant increase in GFP fluorescence in the CTV^- recipient cells, as indicated. Note that ligand gain by recipients is subject to continuous degradation and is not a reliable indicator of total ligand transfer. CD86 transendocytosis is unaffected by PD-L1.

B, C Transendocytosis assays were carried out using CHO cells at a ratio of 2 donor (CD86GFP alone or CD86GFP/PD-L1mCherry): 1 CTLA-4^+ recipient. Transendocytosis assays were performed for the times indicated. Representative FACS plots show CD86GFP downregulation (B) and PD-L1mCherry expression (C) at time points indicated. CD80 and CD80-PD-L1 cell lines have matched expression of ligands.

D Histograms showing CD80GFP and PD-L1mCherry expression levels on transduced DG-75 cell lines. Expression was monitored by measuring GFP (CD80) and mCherry (PD-L1) by flow cytometry.

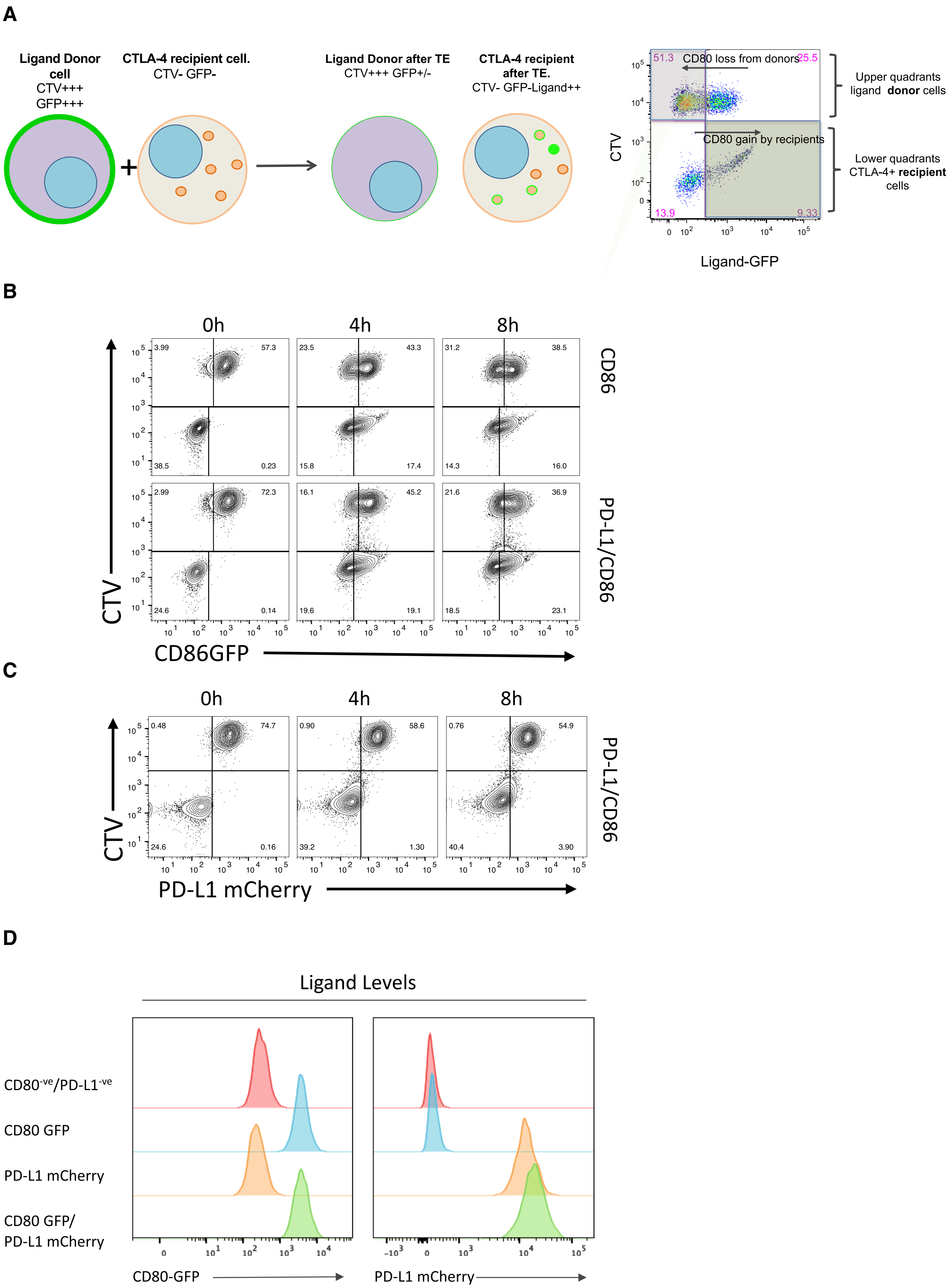

Figure EV2.

 © 2023 The Authors

Figure EV3. Efficient removal of CD80 by transendocytosis liberates free PD-L1 for antibody detection.

A, B Anti-PD-L1 Ab (clone 29E.2A3, at 0.5 μg/ml) was used to detect PD-L1 in DG-75 cells co-expressing CD80GFP (A) or CD86GFP (B). Staining was carried out following transendocytosis with Jurkat cells expressing no CTLA-4, CTLA-4 WT or CTLA-4 Del36 (at a ratio of 1:1) for the indicated durations. (A) shows representative FACS plots of CD80GFP vs. PD-1 Ig at the time points indicated with full kinetic data plotted below (mean ± SEM, three independent experiments, ***$P \leq 0.001$, ****$P \leq 0.0001$: two-way ANOVA with Tukey's multiple comparisons test). (B) As for (A) except using CD86-PD-L1 expressing cells.

C Bar chart comparing the proportion of anti-PD-L1 positive cells after 24 h transendocytosis in DG-75 cells co-expressing PD-L1mCherry and CD80GFP or CD86GFP (mean ± SEM, three independent experiments, ****$P \leq 0.0001$, ns not significant: two-way ANOVA with Tukey's multiple comparisons test).

© 2023 The Authors

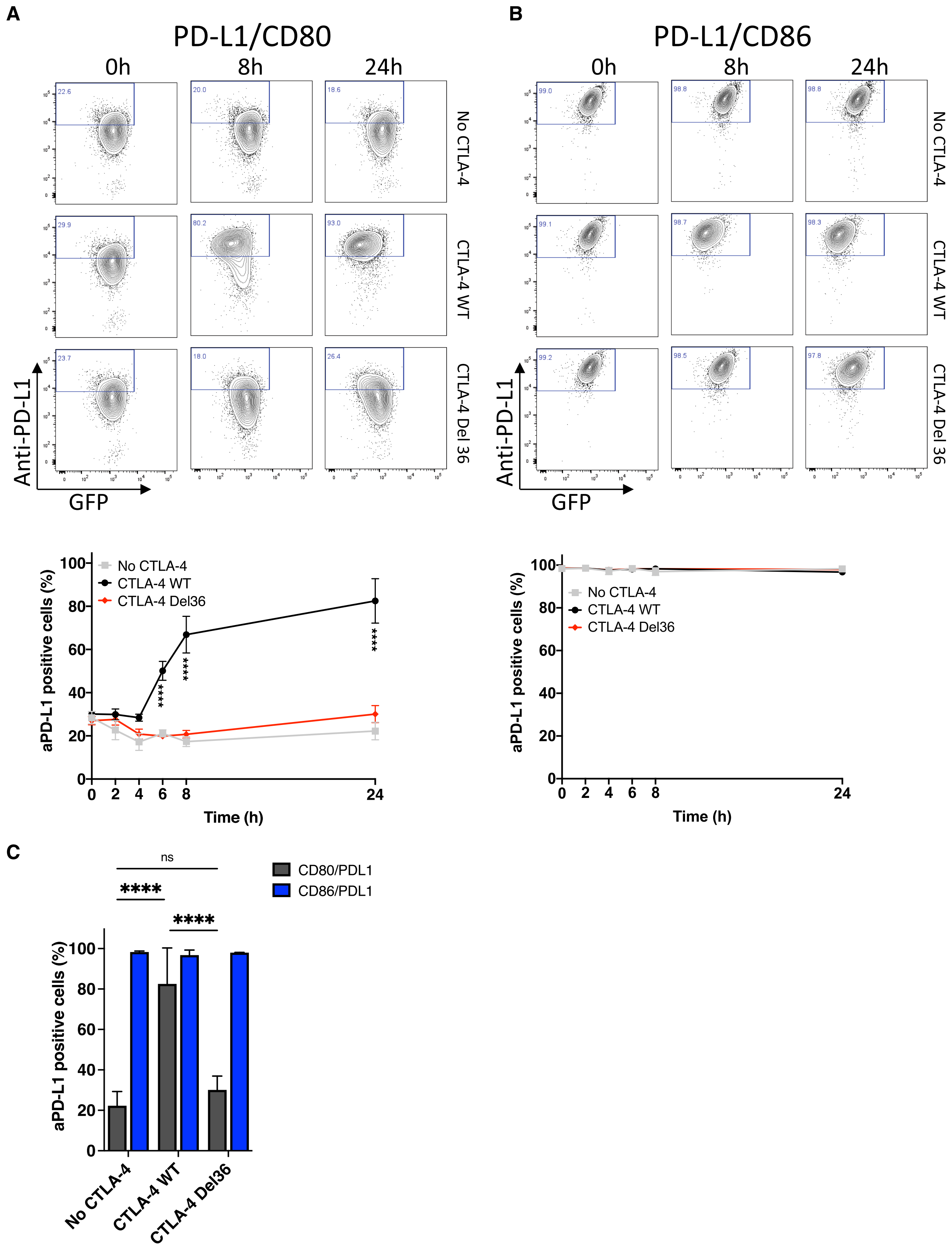

Figure EV3.

 © 2023 The Authors

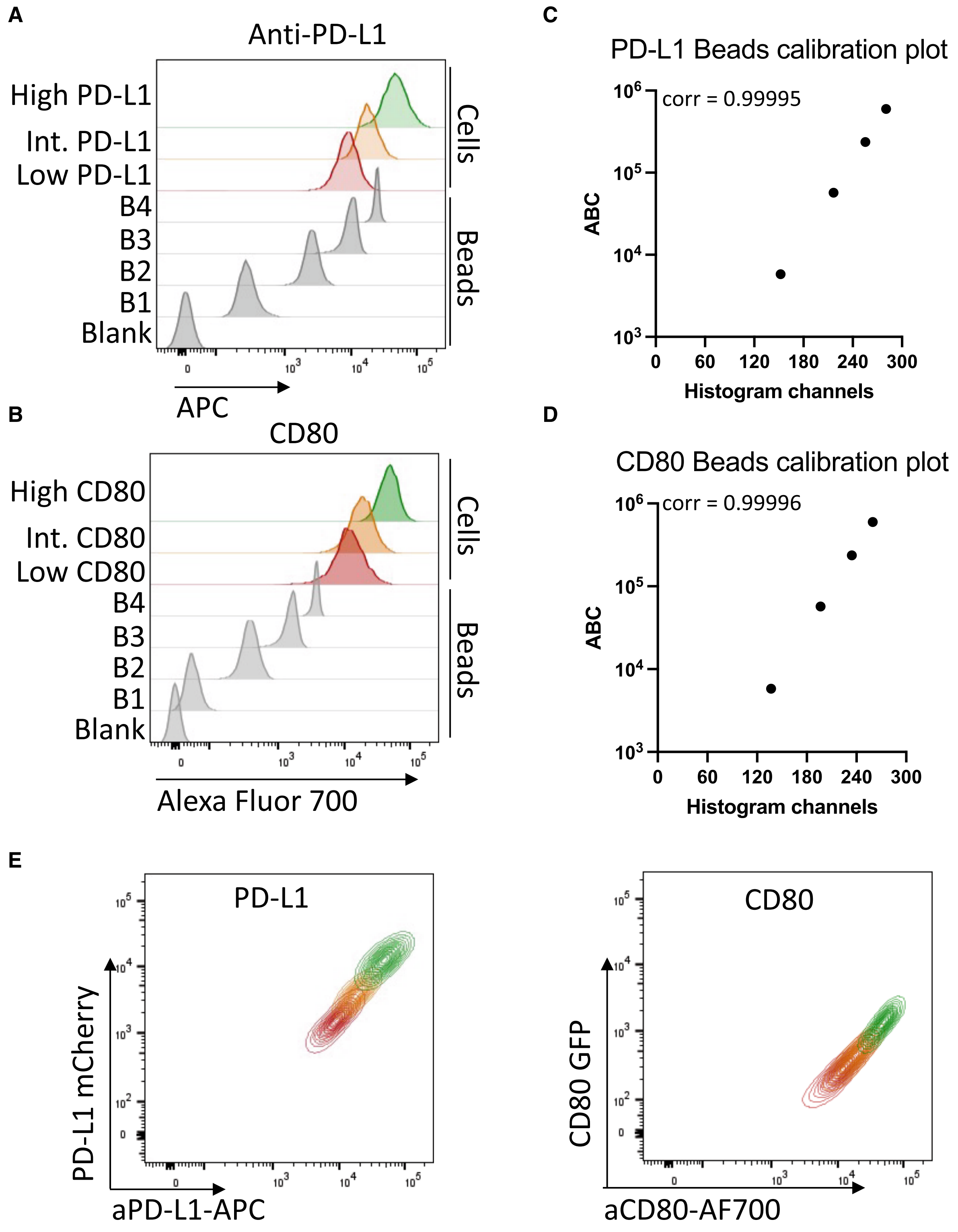
A
Anti-PD-L1
High PD-L1
Int. PD-L1
Low PD-L1
B4
B3
B2
B1
Blank
Cells
Beads
APC
B
CD80
High CD80
Int. CD80
Low CD80
B4
B3
B2
B1
Blank
Cells
Beads
Alexa Fluor 700
C
PD-L1 Beads calibration plot
corr = 0.99995
ABC
Histogram channels
D
CD80 Beads calibration plot
corr = 0.99996
ABC
Histogram channels
E
PD-L1
PD-L1 mCherry
aPD-L1-APC
CD80
CD80 GFP
aCD80-AF700

Figure EV4.

Figure EV4. Calculation of CD80 and PD-L1 ratios on DG-75 cells.

A, B Representative histograms showing antibody staining at saturating concentrations for (A) PD-L1 (clone 29E.2A3, at 25 μg/ml) or (B) CD80 (clone L307.4, at 5 μg/ml) on Quantum™ Simply Cellular® bead populations (grey) or DG-75 cell lines expressing low (red), intermediate (orange) or high (green) levels of respective ligand.

C, D Calibration curves were fitted based on the bead MFI and the corresponding antibody binding capacity (ABC). Representative calibration curves are shown for PD-L1 (C) and CD80 (D) staining with correlation coefficient calculated after curve fitting in the manufacturer-provided QuickCal® spreadsheet.

E Correlation between anti-PD-L1 staining and mCherry intensity, or anti-CD80 staining and GFP intensity in representative FACS plots showing the cell lines from (A) and (B).

Figure EV5. Abatacept immunoprecipitates PD-L1:CD80 interactions.

A Western blot analysis of Abatacept immunoprecipitates from indicated DG-75 cell lines, with and without the BS_3 crosslinker. Precipitates were immunoblotted for GFP (CD80/ CD86) and mCherry (PD-L1) as indicated. Belatacept modulates anti-PD-L1 antibody binding but fails to confer PD-1 Ig detection.

B Concatenated plot of a one in four serial dilution of Belatacept (starting at 20 μg/ml) of CD80 only (top left hand panel) or CD80/PD-L1 co-expressing DG-75 (top right hand panel), with combined titration curves (bottom left hand panel) and EC50 values (bottom right hand values) of Belatacept binding of CD80 only or PD-L1/CD80 co-expressing DG-75. Data are representative of three independent experiments showing mean ± SEM. ns, not significant: paired *t*-test.

C Concatenated plot of a one in four serial dilution of Belatacept (starting at 20 μg/ml) followed by PD-L1 detection using antibody (MIH3 or 29E.2A3 clone) or PD-1 Ig, all at 1ug/ml.

D Graphical representation showing PD-L1 detection under conditions used in (C). Data are representative of three independent experiments showing mean ± SEM.

 © 2023 The Authors

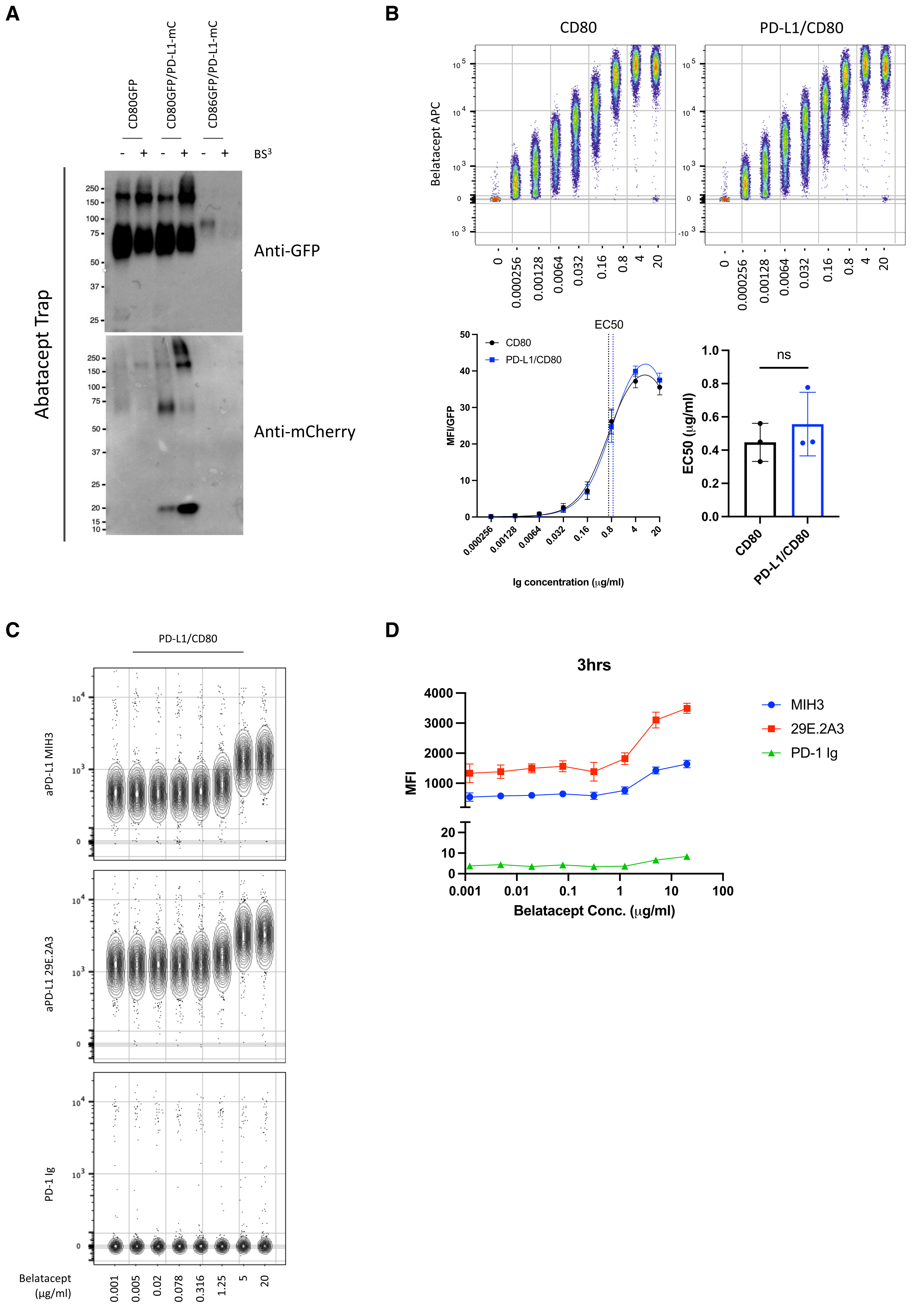
A
CD80GFP
CD80GFP/PD-L1-mC
CD86GFP/PD-L1-mC
BS3
Abatacept Trap
Anti-GFP
Anti-mCherry
B
CD80
PD-L1/CD80
Belatacept APC
EC50
CD80
PD-L1/CD80
MFI/GFP
Ig concentration (μg/ml)
ns
EC50 (μg/ml)
C
PD-L1/CD80
aPD-L1 MIH3
aPD-L1 29E.2A3
PD-1 Ig
Belatacept (μg/ml)
D
3hrs
MFI
MIH3
29E.2A3
PD-1 Ig
Belatacept Conc. (μg/ml)

Figure EV5.
