# Supplementary material for: The CTLA‐4 immune checkpoint protein regulates PD‐L1:PD‐1 interaction via transendocytosis of its ligand CD80
Source: EMBO J. 2023 Feb 2;42(5):e111556. doi: 10.15252/embj.2022111556 (PMC9975936; doi:10.15252/embj.2022111556)
Supplement: Supplementary file 2 — PDF+ [file EMBJ-42-e111556-s003.pdf]

Article

TRANSPARENT PROCESS OPEN ACCESS

THE EMBO JOURNAL

The CTLA-4 immune checkpoint protein regulates PD-L1:PD-1 interaction via transendocytosis of its ligand CD80

Alan Kennedy[1,†], Maximillian A Robinson[1,†], Claudia Hinze[1,†], Erin Waters[1], Cayman Williams[1], Neil Halliday[1], Simon Dovedi[2] & David M Sansom[1,*]

Abstract

CTLA-4 and PD-1 are key immune checkpoint receptors that are targeted in the treatment of cancer. A recently identified physical interaction between the respective ligands, CD80 and PD-L1, has been shown to block PD-L1/PD-1 binding and to prevent PD-L1 inhibitory functions. Since CTLA-4 is known to capture and degrade its ligands via transendocytosis, we investigated the interplay between CD80 transendocytosis and CD80/PD-L1 interaction. We find that transendocytosis of CD80 results in a time-dependent recovery of PD-L1 availability that correlates with CD80 removal. Moreover, CD80 transendocytosis is highly specific in that only CD80 is internalised, while its heterodimeric PD-L1 partner remains on the plasma membrane of the antigen-presenting cell (APC). CTLA-4 interactions with CD80 do not appear to be inhibited by PD-L1, but efficient removal of CD80 requires an intact CTLA-4 cytoplasmic domain, distinguishing this process from more general trogocytosis and simple CTLA-4 binding to CD80/PD-L1 complexes. These data are consistent with CTLA-4 acting as modulator of PD-L1:PD-1 interactions via control of CD80.

Keywords CD80; checkpoint blockade; CTLA4; PD-L1; transendocytosis
Subject Categories Cancer; Immunology
DOI 10.15252/embj.2022111556 | Received 29 April 2022 | Revised 16 December 2022 | Accepted 21 December 2022 | Published online 2 February 2023
The EMBO Journal (2023) 42: e111556

Background

CTLA-4 and PD-1 are two well-established immune checkpoints affecting T cell immune responses. Both pathways are involved in the regulation of autoimmunity, however deficiency in CTLA-4 and PD-1 lead to different disease outcomes. Homozygous CTLA-4 deficiency in mice leads to a fatal lympho-proliferative disease thought to be due to impaired Treg function and consequent activation of self-reactive T cells (Tivol *et al*, 1995; Wing *et al*, 2008; Walker & Sansom, 2011). Likewise in humans, heterozygous mutations have been reported to lead to a spectrum of autoimmune features (Schubert *et al*, 2014). In contrast, the phenotype of PD-1 deficiency is milder but leads to exacerbated autoimmunity in several settings including a lupus-like condition in mice, thought to be related to the lack of attenuation of ongoing T cell responses (Nishimura *et al*, 1999; Freeman *et al*, 2000; Schildberg *et al*, 2016).

Despite somewhat different phenotypes, both CTLA-4 and PD-1 pathways have been targeted in cancer immunotherapy with remarkable efficacy against a range of tumours (Sharma & Allison, 2015; Ribas & Wolchok, 2018). Combination therapies manipulating the early regulator of T cell self-reactivity (CTLA-4) and a later regulator of T cell exhaustion (PD-1) have produced outstanding results in cancer, albeit with autoimmune side effects (Larkin *et al*, 2019). Accordingly, blockade of these two pathways is attracting enormous attention and understanding their interactions is therefore important.

At the molecular level, CTLA-4 function remains incompletely understood with several proposed mechanisms (Walker & Sansom, 2015; Schildberg *et al*, 2016). However, it is clear that CTLA-4 on Treg and activated conventional T cells binds to two ligands (CD80 and CD86) found on APCs. These same ligands are used by CD28 to activate T cells and therefore interaction with higher affinity CTLA-4 reduces CD28 function (Halliday *et al*, 2020). In addition to direct competition for ligand binding, we have found that CTLA-4 is able to physically deplete its ligands from APCs in a process termed transendocytosis (Qureshi *et al*, 2011). Here, CTLA-4 binds its ligands and transfers them into the T cell followed by their degradation, although the fate of CTLA-4 itself is different depending on the ligand bound (Kennedy *et al*, 2022). Whilst there are similarities between transendocytosis and another process termed trogocytosis (Daubeuf *et al*, 2010), in that both lead to intercellular transfer of proteins, in our experience transendocytosis is detected as a time-dependent transfer of protein between cells, which remain in cell–cell contact. In contrast, trogocytosis is generally revealed following cell separation. Accordingly, transendocytosis exploits the dynamic intracellular trafficking of CTLA-4 (Qureshi

1 UCL Institute of Immunity and Transplantation, London, UK
2 Early Oncology R&D, AstraZeneca, UK
*Corresponding author. Tel: +44 020 7794 0500; E-mail: d.sansom@ucl.ac.uk
†These authors contributed equally to this work

© 2023 The Authors. Published under the terms of the CC BY 4.0 license.

et al, 2012) to efficiently harvest CD80 and CD86 ligands from target cells. This allows CTLA-4 to generate a cell extrinsic control mechanism compatible with its requirement in Treg function (Wing *et al*, 2008; Walker & Sansom, 2011). It is thought that this regulatory function of CTLA-4 operates constitutively in order to prevent costimulation of self-reactive T cells in the steady state, since loss of CTLA-4 function triggers profound autoimmunity in mice and humans (Tivol *et al*, 1995; Kuehn *et al*, 2014; Schubert *et al*, 2014).

In contrast to CTLA-4, the PD-1 pathway appears to control the ongoing activity of CD4 and CD8 T cells following immune activation (Wei *et al*, 2017, 2019). Like CTLA-4, PD-1 also binds to two ligands (PD-L1 and PD-L2), which whilst expressed on APCs also have a much wider tissue distribution (Schildberg *et al*, 2016). The expression of PD-1 is upregulated on T cell activation, although it is often considered to be associated with "exhausted" or pre-exhausted T cells resulting from chronic exposure to antigen either due to infection or due to self-reactivity (Wherry & Kurachi, 2015; Kallies *et al*, 2020). Engagement of PD-1 with its ligands appears to drive this "exhausted" phenotype since blockade of PD-1 can reinvigorate T cells responses in viral infection and in cancer.

Despite evidence for distinct functional pathways, CD28/CTLA-4 and PD-1 pathways are connected at the molecular level. The inhibitory signalling pathway triggered by PD-1 appears to be capable of targeting both TCR and CD28 signalling. Recent studies have suggested that recruitment of the phosphatase Shp2 to PD-1 may result in a preferential targeting of the CD28 pathway over the TCR (Hui *et al*, 2017; Kamphorst *et al*, 2017), although this remains controversial (Mizuno *et al*, 2019). In addition, a direct interaction between CD80 and PD-L1 has been observed to occur with significant affinity (Butte *et al*, 2007, 2008). Whilst several studies have inferred an intercellular "*trans*" interaction (Butte *et al*, 2007; Ni *et al*, 2017; Cassady *et al*, 2018), more recent evidence suggests that this interaction takes place predominantly *in cis* between CD80 and PD-L1 on the same cell (Chaudhri *et al*, 2018; Sugiura *et al*, 2019). The result of this interaction is that CD80 and PD-L1 physically associate in a manner that blocks PD-L1 binding to PD-1, but potentially allows CD80 to continue its interactions with CD28 and CTLA-4. However, it remains unclear whether CD80 simply regulates the PD-1 pathway or whether PD-L1 also acts as a regulator of the CD28/CTLA-4 pathway as has been suggested (Zhao *et al*, 2019). We have therefore addressed the role of CTLA-4 transendocytosis in regulating these processes.

Given that CTLA-4 efficiently targets CD80 via transendocytosis we investigated how transendocytosis affects the PD-1 pathway. We observed that PD-L1 did not prevent CTLA-4 binding to CD80 and that CTLA-4-dependent transendocytosis remained effective despite CD80 hetero-dimerisation with PD-L1. Furthermore, despite the fact that PD-L1 was found at the immune synapse along with CD80 during transendocytosis, PD-L1 was not removed and remained on the APC. Thus, CTLA-4 demonstrated a highly selective, time-dependent capacity to remove CD80 from APCs, which required the CTLA-4 cytoplasmic domain. Whilst some ligand transfer by trogocytosis was observed in cells expressing a tailless CTLA-4 mutant, this did not result in effective downregulation of CD80 from donor cells. Finally, depletion of CD80 by transendocytosis led to a time-dependent restoration of free PD-L1 across the cell membrane, a feature not replicated by soluble CTLA-4-Ig binding. Free PD-L1 was capable of engaging PD-1 and inhibiting events associated with TCR signalling. These data show that CD80 is a regulator of PD-L1:PD-1 interactions but not vice versa. Moreover, efficient ligand depletion by transendocytosis, but not simple CTLA-4 binding to CD80, is required to effectively liberate PD-L1 that can engage PD-1.

Results

Co-expression of CD80 and PD-L1 on the same cell disrupts PD-1 but not CTLA-4 or CD28 recognition

To study the interactions between PD-L1 and CD80 we initially used transduced cells expressing various combinations of the relevant proteins. PD-L1 was tagged with a cytoplasmic mCherry tag and probed with different anti-PD-L1 ectodomain antibodies or with CD80-Ig, PD-1-Ig or CTLA-4-Ig. When PD-L1 was expressed in isolation it was clearly detected by all PD-L1 antibodies as well as PD-1 Ig, indicating that PD-L1 was correctly expressed (Figs 1A and EV1A). Interestingly, binding of CD80-Ig to PD-L1 was surprisingly poor, presumably reflecting the unfavourable binding of CD80 and PD-L1 in "*trans*" (Chaudhri *et al*, 2018; Sugiura *et al*, 2019). In marked contrast, co-expression of CD80 on the same cell as PD-L1 inhibited staining with several different PD-L1–specific antibodies and was particularly notable when using soluble PD-1 (Figs 1B and D, and EV1A). The level of inhibition was dependent on the antibody used, presumably reflecting different epitopes and affinities. Moreover, dose–response curves using a clinical anti-PDL1 antibody (Durvalumab) also showed inhibition by CD80 was evident but could be overcome at high doses of antibody (Fig EV1B). Whilst there was a loss of PD-L1 detection upon CD80 co-expression, staining of CD80 itself was readily detectable with abatacept (CTLA-4-Ig) (Figs 1B and EV1A), suggesting that CTLA-4 binding to CD80 was not obviously impaired by the presence of co-expressed PD-L1. Furthermore, using CD28 downregulation as a measure of ligand engagement, we also confirmed that PD-L1 had no impact on the ability of CD80 or CD86 to engage with CD28 (Fig EV1C). Finally, in contrast to CD80, the co-expression of CD86 had no detectable impact on the binding of PD-L1 reagents or vice versa, highlighting clear differences between CD80 and CD86 (Fig 1C and D, and EV1A and C).

Using immunoprecipitation and Western blotting, we also observed that precipitation of PD-L1 co-precipitated CD80GFP upon chemical crosslinking, confirming the formation of a heterodimer between CD80 and PD-L1, which was not seen with the non-interacting CD86 (Fig 1E). Taken together these data were highly consistent with previous observations (Chaudhri *et al*, 2018; Sugiura *et al*, 2019) showing that co-expression of PD-L1 with CD80 in *cis* resulted in inhibition of PD-L1 interactions but retained CD80 binding to CTLA-4.

Transendocytosis by CTLA-4 selectively removes CD80 but not PD-L1

Transendocytosis is a molecular process whereby the CTLA-4 ligands, CD80 and CD86, are physically removed from their host cell by a CTLA-4 expressing cell and internalised, resulting in their destruction (Qureshi *et al*, 2011). Previously it has been suggested that CD80:PD-L1 interactions can disrupt CTLA-4 binding and

© 2023 The Authors

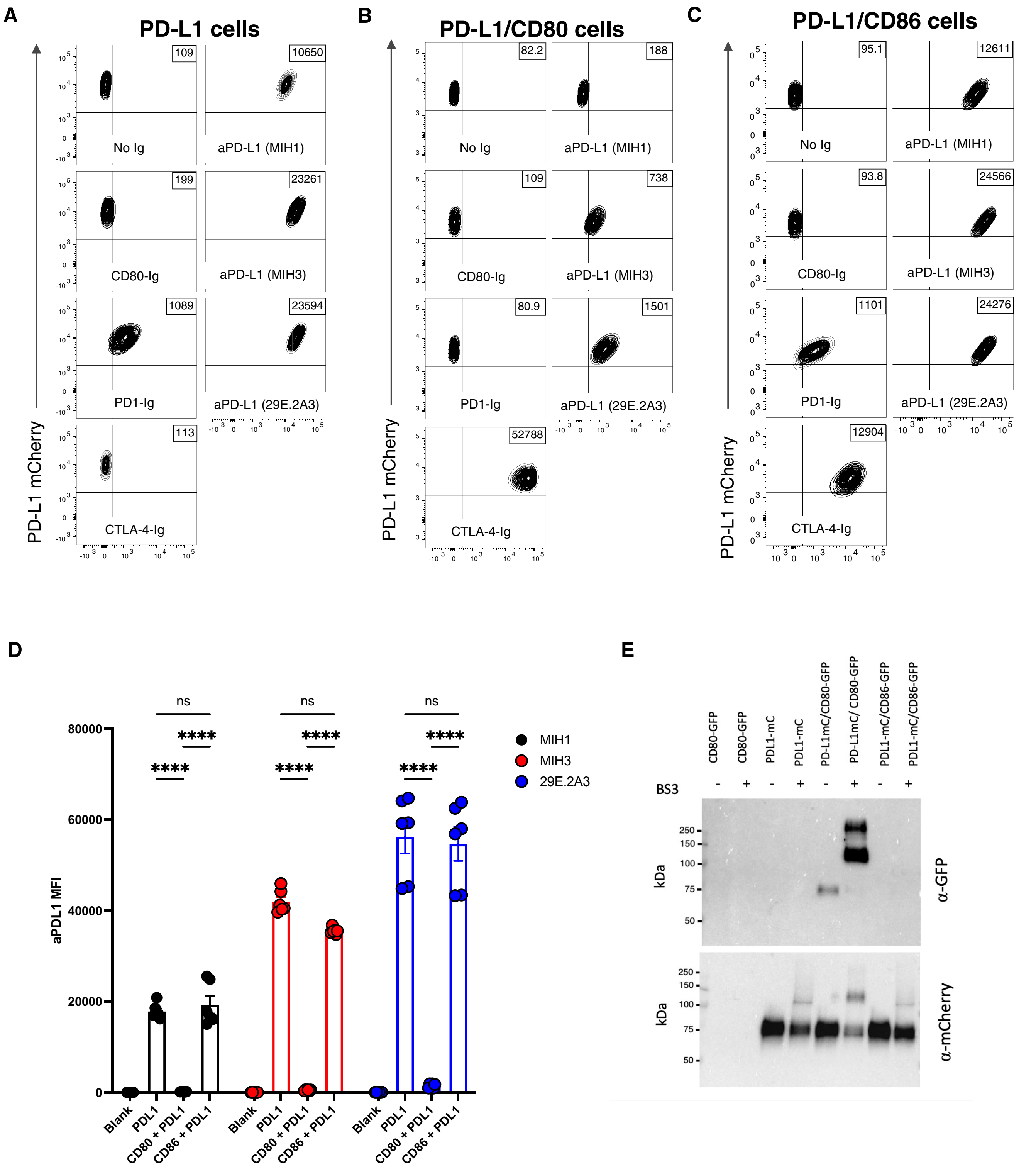

Figure 1. Expression of CD80 and PD-L1 *in cis* inhibits PD-L1 detection.

A–C CHO cells expressing PD-L1 alone (A) or co-expressing CD80 (B) or CD86 (C) were stained with CD80 Ig, PD-1 Ig, CTLA-4 Ig or anti-PD-L1 Ab clones MIH1, MIH3, 29E.2A3) at 1 μg/ml for 30 min at 37°C. Representative FACS plots show staining plotted against total PD-L1 mCherry levels.

D Integrated data showing PD-L1 detection as determined by the indicated 3 aPD-L1 Ab clones (mean ± SEM, two repeats from three independent experiments, $****P \leq 0.0001$: two-way ANOVA with Tukey's multiple comparisons test).

E Western blot analysis of an immunoprecipitation by anti-mCherry antibody (PDL1) from indicated DG-75 cell lysates, with and without the BS_3 crosslinker, and probed for mCherry (PD-L1) or GFP (CD80).

© 2023 The Authors

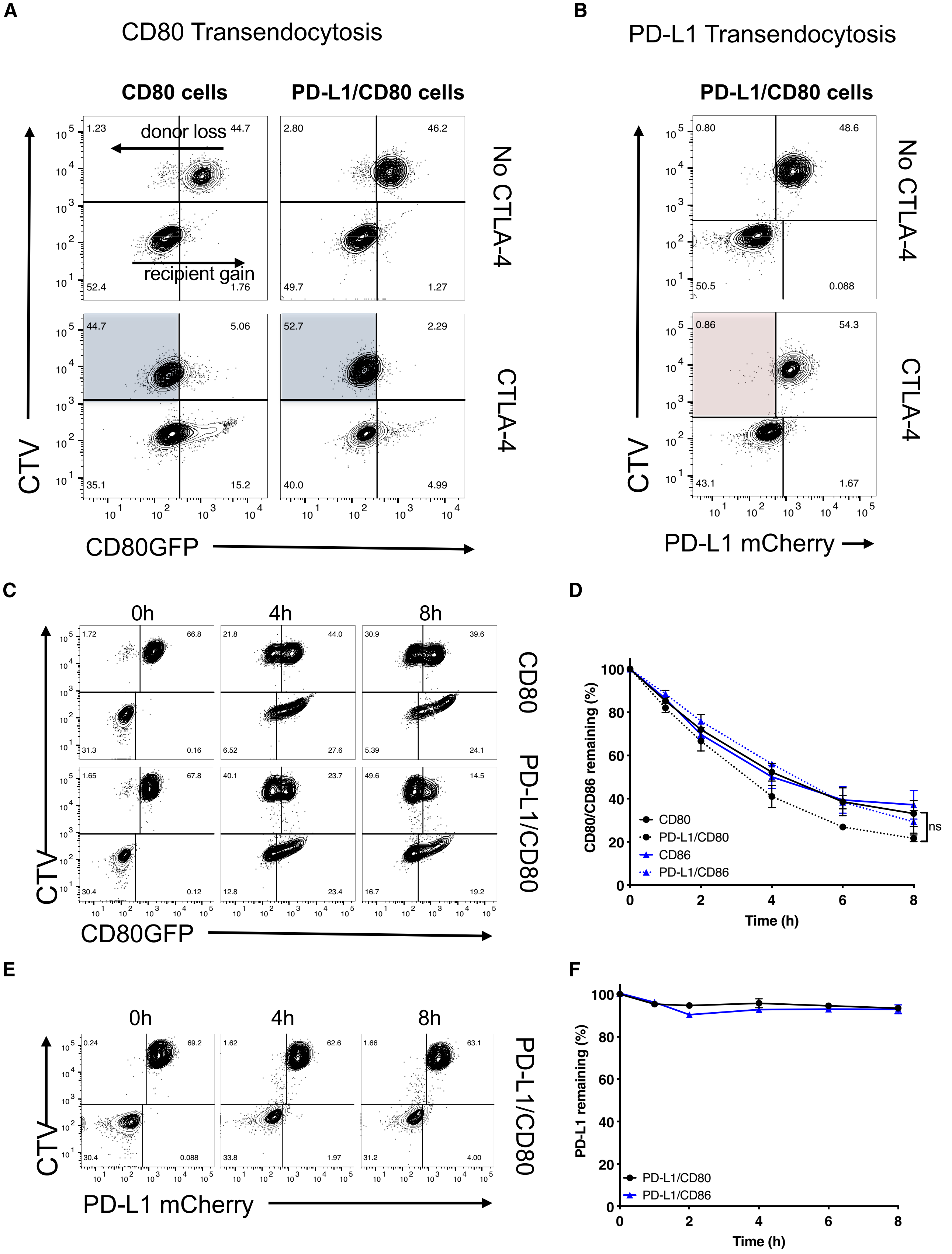

Figure 2.

© 2023 The Authors

Figure 2. Transendocytosis of CD80 is not inhibited by PD-L1 co-expression.

A Transendocytosis assays were carried out overnight (as detailed in EV2) using CHO donor cells (CD80GFP alone or CD80GFP with PD-L1mCherry co-expression) incubated with CTLA-4 recipient cells at a ratio of 1:1. Recipient cells either lacked CTLA-4 (No CTLA-4) or expressed CTLA-4. FACS plots show CD80GFP ligand loss from donor cells highlighted in blue quadrants in the presence of CTLA-4$^+$ recipients.

B Assays as in (A) but showing PD-L1 expression following CD80 transendocytosis (red shaded quadrant).

C–F Transendocytosis assays were performed at a ratio of 2 donor:1 CTLA-4 recipient for the times indicated. (C), Representative FACS plots showing CD80GFP levels and (D) full kinetic analysis of CD80 or CD86 downregulation on donor cells quantified as a percentage relative to no CTLA-4 control (mean ± SEM, three independent experiments, ns, not significant: two-way ANOVA with Tukey's multiple comparisons test). (E) Representative FACS plots showing PD-L1mCherry level at time points indicated. (F) Full kinetic analysis of PD-L1mCherry level on donor cells (mean ± SEM, three independent experiments).

therefore transendocytosis (Zhao *et al*, 2019). We therefore tested the impact of PD-L1 on CD80 transendocytosis by measuring the loss of GFP-labelled CD80 ligands from donor cells as well as their acquisition by the CTLA-4$^+$ recipient population using a well-established assay (Fig EV2A). This revealed that in the presence of CTLA-4, CD80 was very effectively removed from the CTV-labelled ligand donor cells irrespective of PD-L1 co-expression (Fig 2A, blue shaded quadrants). By tracking the PD-L1 mCherry signal in the same assay we observed that PD-L1mCherry remained completely associated with the donor cell despite the near total removal of CD80, indicating that CTLA-4 did not target PD-L1 for removal even when it was associated with CD80 (Fig 2A and B red quadrant). Thus, we concluded that whilst CD80 could prevent PD-L1 interactions with the PD-1 receptor, the reverse was not true and CD80 remained able to functionally interact with CTLA-4 and undergo transendocytosis. Moreover, the transendocytosis process demonstrated remarkable selectivity in that PD-L1 was not removed despite its CD80 partner being captured by CTLA-4.

Given that transendocytosis is a dynamic process controlled by both the amount of CTLA-4 present and the contact time (Hou *et al*, 2015), we monitored the impact of transendocytosis over time. As shown in Fig 2C, loss of CD80 from the donor cell and its uptake by recipient cells continued over time in both the presence and absence of PD-L1. CD86 transendocytosis was also unaffected (Fig EV2B), in keeping with its lack of interaction with PD-L1. Despite the effective removal of CD80 ligand over time, with no significant difference in CD80 removal observed in the presence or absence of PD-L1 (Fig 2D), there was no indication of significant PD-L1 depletion from the CD80-PD-L1 or CD86-PD-L1 donor cells at any time point (Figs 2E and F and EV2C). Whilst we noted a very slight increase in mCherry signals in the CTLA-4 recipient cells, this did not result in appreciable downregulation of PD-L1 from the donor cells in stark contrast to the downregulation of CD80. Taken together, the above data revealed that recognition of CD80 by CTLA-4 was not inhibited by the presence of PD-L1. Furthermore, transendocytosis of CD80 continued over extended times without appreciably removing PD-L1, demonstrating the remarkable specificity of transendocytosis and its ability to regulate PD-L1 availability.

Efficient removal of ligand by transendocytosis requires the CTLA-4 cytoplasmic domain

The above data showed the impact of human CTLA-4 on transendocytosis when expressed in CHO cells, which have proven a reliable model for transendocytosis (Qureshi *et al*, 2011; Schubert *et al*, 2014; Hou *et al*, 2015; Khailaie *et al*, 2018). Nonetheless, to verify these observations in immune cells, we repeated these experiments using CTLA-4$^+$ Jurkat T cells and B cells (DG-75) expressing matched levels of CD80 or PD-L1 (Fig EV2D), to allow the formation of an immune synapse. Using this system, we also investigated the role of the CTLA-4 cytoplasmic domain, since it has been suggested that CTLA-4 can remove CD80 from CD80:PD-L1 co-expressing cells via trogocytosis (Tekguc *et al*, 2021), a process not requiring its cytoplasmic domain.

In line with our observations in CHO cells, CD80 was effectively downregulated over time by wild-type (WT) CTLA-4 (Fig 3A). The downregulation of CD80 and CD86 was kinetically similar (Fig 3A–D) with ligand donor cells being depleted by approximately 50% after 4 h. In contrast, trogocytosis by CTLA-4 Del36 (which lacks the 36 amino acids comprising the cytoplasmic domain) was much more limited, reaching only ~20% downregulation by 24 h. Although the amount of ligand acquired by the CTLA-4 recipient cells appeared similar between WT and Del36 (e.g. lower right quadrants, Fig 3A) this did not reflect the level of ligand downregulation from the donor cells (upper left quadrants), since ligand is continually degraded inside CTLA-4 cells. In contrast, monitoring ligand downregulation (Fig 3A and B—upper left quadrants) showed that transendocytosis by WT CTLA-4 was much more effective in removing CD80 and CD86, than was trogocytosis by the Del36

Figure 3. The CTLA-4 cytoplasmic domain promotes efficient transendocytosis of CD80 and CD86.

A, B CTV-labelled B cells (DG-75) co-expressing PD-L1mCherry and CD80GFP (A) or PD-L1mCherry and CD86GFP (B) were incubated with Jurkat cells expressing no CTLA-4, CTLA-4 WT or a mutant CTLA-4 (Del36) lacking the cytoplasmic tail. Transendocytosis was carried out at a 1:1 ratio for the indicated times and analysed by flow cytometry. FACS plots show GFP ligand loss from donor B cells (upper left quadrants) and acquisition by Jurkat recipients (lower right quadrants).

C Levels of CD80GFP and PD-L1mCherry remaining on donor cells over a 24 h transendocytosis period with CTLA-4 WT or Del36, plotted as a percentage relative to no CTLA-4 control (mean ± SEM, six independent experiments). $^{****}P \leq 0.0001$, RM one-way ANOVA.

D Levels of CD86GFP and PD-L1mCherry remaining on donor cells over a 24 h transendocytosis period with CTLA-4 WT or Del36 plotted relative to no CTLA-4 control (mean ± SEM, six independent experiments). $^{****}P \leq 0.0001$, RM one-way ANOVA.

E Comparison of CTLA-4 surface expression levels in Jurkat cells expressing CTLA-4 WT or CTLA-4 Del36 as determined by anti-CTLA-4 (clone BNI3 at 1:100 dilution) stain on ice and analysed by flow cytometry. Graph shows mean ± SEM from four independent experiments, $^{**}P \leq 0.01$, Student's two-tailed independent samples *t*-test.

© 2023 The Authors

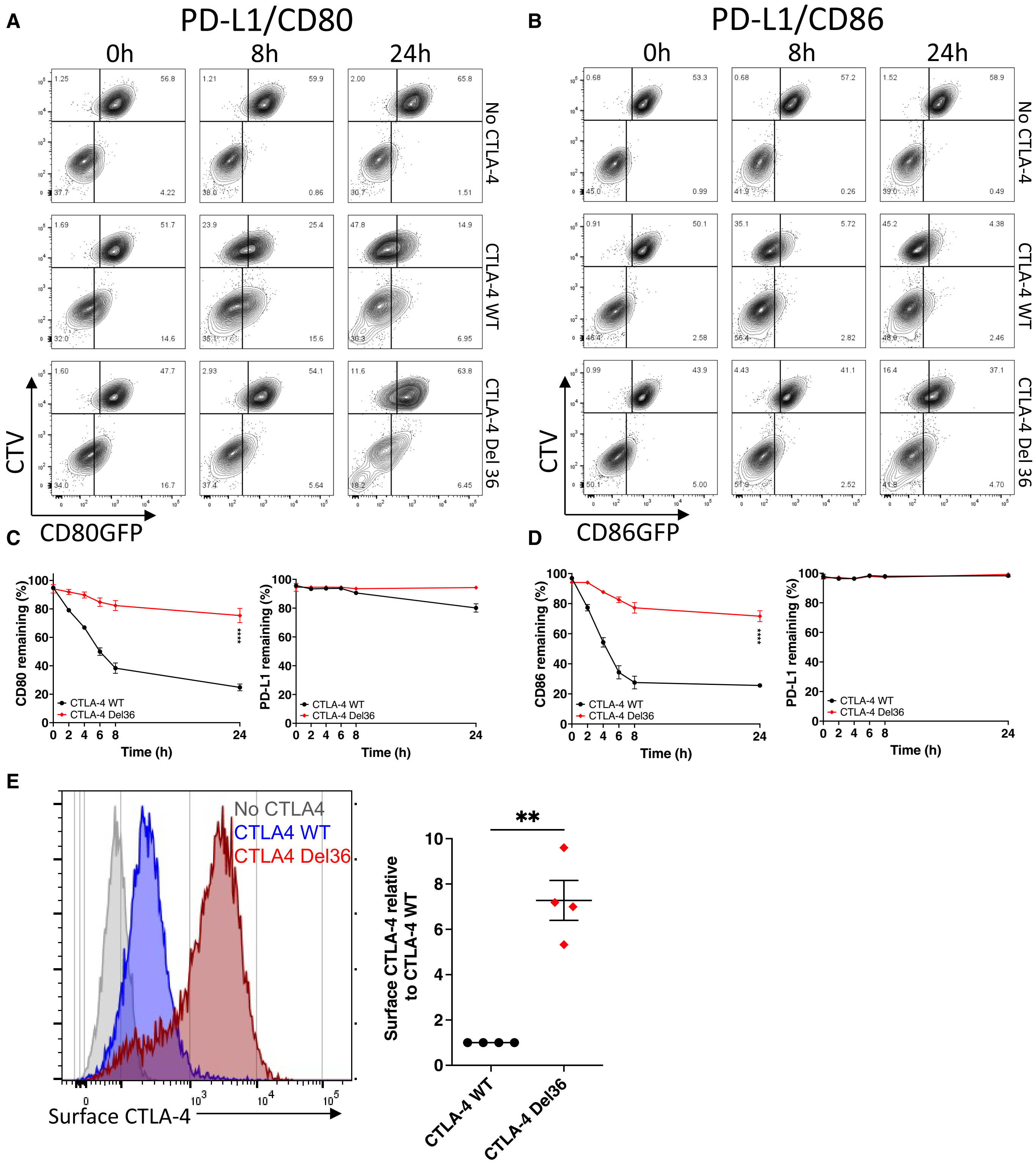

Figure 3.

mutant. This result was more notable since Del36 cells also expressed higher levels of CTLA-4 at the cell surface, due to defective CTLA-4 endocytosis (Fig 3E). We also evaluated the downregulation of PD-L1 in these experiments and confirmed again that PD-L1 remained stably associated with the donor cell in all settings (Fig 3C and D). Together these data clearly established that the CTLA-4 cytoplasmic domain promoted effective time-dependent depletion of CD80 and CD86 ligands but that PD-L1 was not effectively removed from donor cells via either transendocytosis or trogocytosis.

© 2023 The Authors

Transendocytosis of CD80 effectively liberates PD-L1 over time

Given that CD80:PD-L1 interactions *in cis* prevent the binding of PD-1, it is predicted that the physical removal of CD80 via transendocytosis should restore the availability of free PD-L1. We therefore investigated recovery of PD-1 Ig binding over time in transendocytosis assays. We observed that PD-1 Ig did not bind to cells co-expressing CD80 and PD-L1 at the outset or in the absence of CTLA-4, whereas binding to CD86/PD-L1 cells was unimpaired (Fig 4A and B, top rows). However, in the presence of WT CTLA-4 (middle rows), the loss of CD80GFP over time via transendocytosis progressively allowed detection of PD-L1 by PD-1 Ig. Depletion of CD80 was both CTLA-4 dependent and time-dependent, with a clear inverse correlation between the level of CD80GFP remaining and the detection of PD-L1 at the cell surface (Fig 4A). Once again, the ability of CTLA-4 Del36 to recover PD-1 Ig binding was substantially less (albeit detectable) compared to WT CTLA-4, in keeping with its weaker ability to effectively remove CD80 (Figs 4A and 3A). As expected, whilst transendocytosis also led to reduced expression of CD86, this had no impact on PD-L1 detection, in keeping with the

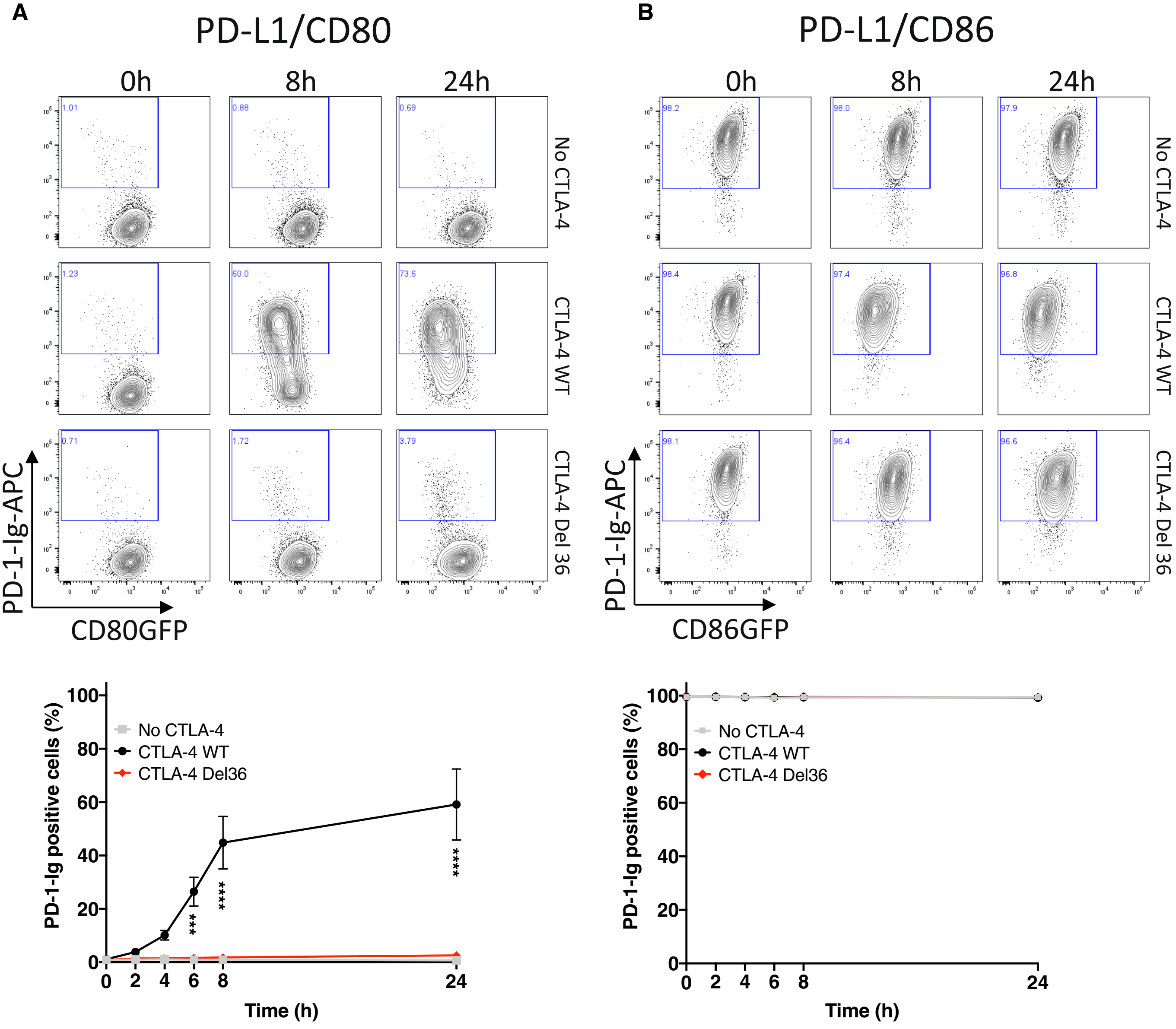

Figure 4. Efficient removal of CD80 by transendocytosis liberates free PD-L1 and restores PD-1 Ig binding.

A, B APC-labelled PD-1 Ig (0.75 μg/ml) was used to detect PD-L1 in DG-75 cells co-expressing CD80GFP (A) or CD86GFP (B). Staining was carried out following transendocytosis using Jurkat cells expressing no CTLA-4, CTLA-4 WT or CTLA-4 Del36 (at a ratio of 1:1) for the indicated durations. (A) shows representative FACS plots of CD80GFP vs. PD-1 Ig at the time points indicated, with full kinetic data plotted below (mean ± SEM, three independent experiments, ****P* ≤ 0.001, *****P* ≤ 0.0001: two-way ANOVA with Tukey's multiple comparisons test). (B) As for (A) except using CD86-PD-L1 expressing cells.

© 2023 The Authors

lack of CD86:PD-L1 interaction (Fig 4B). These data demonstrated that binding of PD-1 Ig was remarkably sensitive to changes in CD80 levels and that PD-L1 availability was related to the level of CD80 expression. We repeated these experiments using anti-PD-L1 antibodies rather than PD-1 Ig to detect PD-L1. In this case, the reduction in anti-PD-L1 staining due to CD80 co-expression was less marked than for PD-1 Ig staining, indicating that PD-L1 detection was dependent on the reagents used. Nonetheless, PD-L1 staining was still impaired in the presence of CD80 (Fig EV3A). Once again transendocytosis of CD80 by WT CTLA-4 markedly increased detection such that after 24 h, PD-L1 staining was comparable to the level seen in the CD86 co-expressing cells, where no significant inhibition of PD-L1 staining had occurred (Fig EV3B and C). In keeping with the above findings, the CTLA-4 Del36 mutant did not effectively restore PD-L1 staining—although there was a limited improvement in staining following trogocytosis for 24 h this was not found to be significant. Together these data revealed differences between the ability of PD-1-Ig and antibody to detect PD-L1 as a result of CD80 interaction and that PD-1-Ig binding was generally more sensitive to inhibition by CD80.

Given the clear relationship between CD80 expression and the ability of PD-L1 to bind to PD-1-Ig, we carried out further experiments to establish the stoichiometry at which CD80 prevents PD-L1: PD-1 interaction. We therefore quantified the number of CD80 molecules and PD-L1 molecules on several independent cell lines (Fig EV4A–E) and calculated the ratios of CD80:PD-L1 (Fig 5A). PD-1-Ig staining of these cells revealed that at ratios of ~ 4:1, PD1-Ig binding was highly impaired compared to cells with no CD80 (Fig 5A). To complement this approach, we carried out a more detailed analysis by taking a single cell line with defined PD-L1 expression and plotting a heat map of PD-1-Ig staining relative to CD80GFP expression (Fig 5B). By gating on different levels of CD80 and calculating the ratio of PD-L1:CD80 we could then plot PD-1-Ig binding at different ratios. Aggregated data from several independent experiments showed that above a CD80:PD-L1 ratio of 1:1, PD-1 binding was strongly inhibited (Fig 5C) suggesting that a 1:1 stoichiometry between CD80 and PD-L1 is effective in inhibiting PD-1 binding.

We next examined the time-dependent recovery of PD-1 Ig binding following transendocytosis using confocal microscopy, with similar results to our FACS experiments (Fig 6A). These data revealed clear synaptic contacts between CTLA-4$^+$ Jurkat cells and B-cells expressing CD80 or CD86 alongside PD-L1. In addition, transferred CD80 or CD86 could be observed as green vesicles inside of CTLA-4$^+$ cells; however, there was no evidence of transferred PD-L1 inside CTLA-4$^+$ cells. As expected, CD86/PD-L1 cells showed clear plasma membrane staining by PD-1 Ig as shown by purple staining at all time points, consistent with CD86 having no impact on PD-1 Ig binding (Fig 6A—left column). In contrast, PD-1 Ig staining gradually recovered in CD80/PD-L1 cells at later time points, as CD80 was removed by WT CTLA-4 (Fig 6A—middle column). Again, the CTLA-4 Del36 mutant, whilst showing some evidence of CD80GFP uptake, did not sufficiently deplete CD80 to enable PD-1 Ig detection (Fig 6A—right column), showing that high levels of CTLA-4 binding *per se* did not release PD-L1. Accordingly, during maintained cell–cell contacts, only WT CTLA-4 effectively removed CD80 to reveal PD-1:PD-L1 interactions (Fig 1). Interestingly, previous data have indicated that the physical binding of CTLA-4 to the CD80-PD-L1 heterodimer, might induce dissociation of PD-L1 and CD80 (Garrett-Thomson *et al*, 2020). In such a case, we might expect that PD-1 detection would focus at the synapse, where either WT or Del36 engagement of CD80 should liberate free PD-L1, even at early time points. However, we did not observe such an effect, despite clear evidence of CTLA-4:CD80 contacts (yellow synapse), indicating other parameters must affect this outcome. Together these data highlighted a requirement for the physical removal of CD80 by CTLA-4 transendocytosis and that simple binding of CD80 by CTLA-4 did not liberate free PD-L1 capable of binding PD-1.

Transendocytosis of CD80 liberates functional PD-L1

The above data unequivocally show that efficient removal of CD80 by transendocytosis restores the ability of PD-L1 to bind to soluble PD-1-Ig. To test whether PD-L1 was functionally capable, we designed a "3-cell" assay (Fig 7A) where CTLA-4 expressing Jurkat cells (Jreg) were used to regulate CD80 expression on DG-75 APCs, which expressed CD80 and PD-L1. As a readout cell, we used CTV-labelled responder Jurkat cells, which expressed PD-1 and measured their response to the superantigen SEE via CD69 expression. At 24 h, CD80 expression on the APC was downregulated by WT CTLA-4 (Fig 7B). Conversely, CD28 expression on the PD-1$^+$ responder Jurkat, was downregulated by CD80 engagement and prevented by transendocytosis, establishing that the PD-1$^+$ responders contacted APCs and could sense changes in CD80 levels (Fig 7C). We then measured CD69 expression as a marker of activation on the PD-1$^+$ responder cells and observed that in the presence of WT CTLA-4, CD69 expression was significantly lower, in keeping with PD-1 inhibition of CD69 expression downstream of TCR signalling (Fig 7D). Moreover, this downregulation of CD69 was reversed in the presence of blocking PD-1 antibodies. Finally, because in the above system the responder Jurkat cell was exposed to different levels of CD80 (and therefore CD28 costimulation) as well as PD-1 engagement, we repeated these experiments using a CD28 negative PD-1+ responder cell. This gave similar results, indicating that the control of CD69 via PD-1 engagement was via the TCR and not CD28 signalling (Fig 7E). Moreover, the ability of PD-1 to reduce CD69 expression was observed over a wide range of SEE doses (Fig 7F). Together these data indicated that functional PD-L1:PD-1 interactions were revealed by the presence of WT CTLA-4 and not by the Del36 CTLA-4 mutant and that PD-L1 release resulting from CD80 transendocytosis could then functionally engage PD-1 and inhibit TCR signals.

Abatacept alters antibody detection of PD-L1 but does not confer PD-1 binding

The above data indicated CD80 removal by CTLA-4 transendocytosis, but not simple CTLA-4 binding, was required for liberation of competent PD-L1 expression. We therefore tested this concept in a second experiment, studying the ability of soluble CTLA-4 (abatacept) to liberate PD-L1 from CD80-PD-L1 heterodimers. It has recently been shown that soluble CTLA-4 can enhance antibody detection of PD-L1 on cells expressing both CD80 and PD-L1 (Tekguc *et al*, 2021). However, the reason for such an effect is not clear, since abatacept binds effectively to CD80 as part of a CD80: PD-L1 heterodimer and can be used to co-immunoprecipitate PD-

 © 2023 The Authors

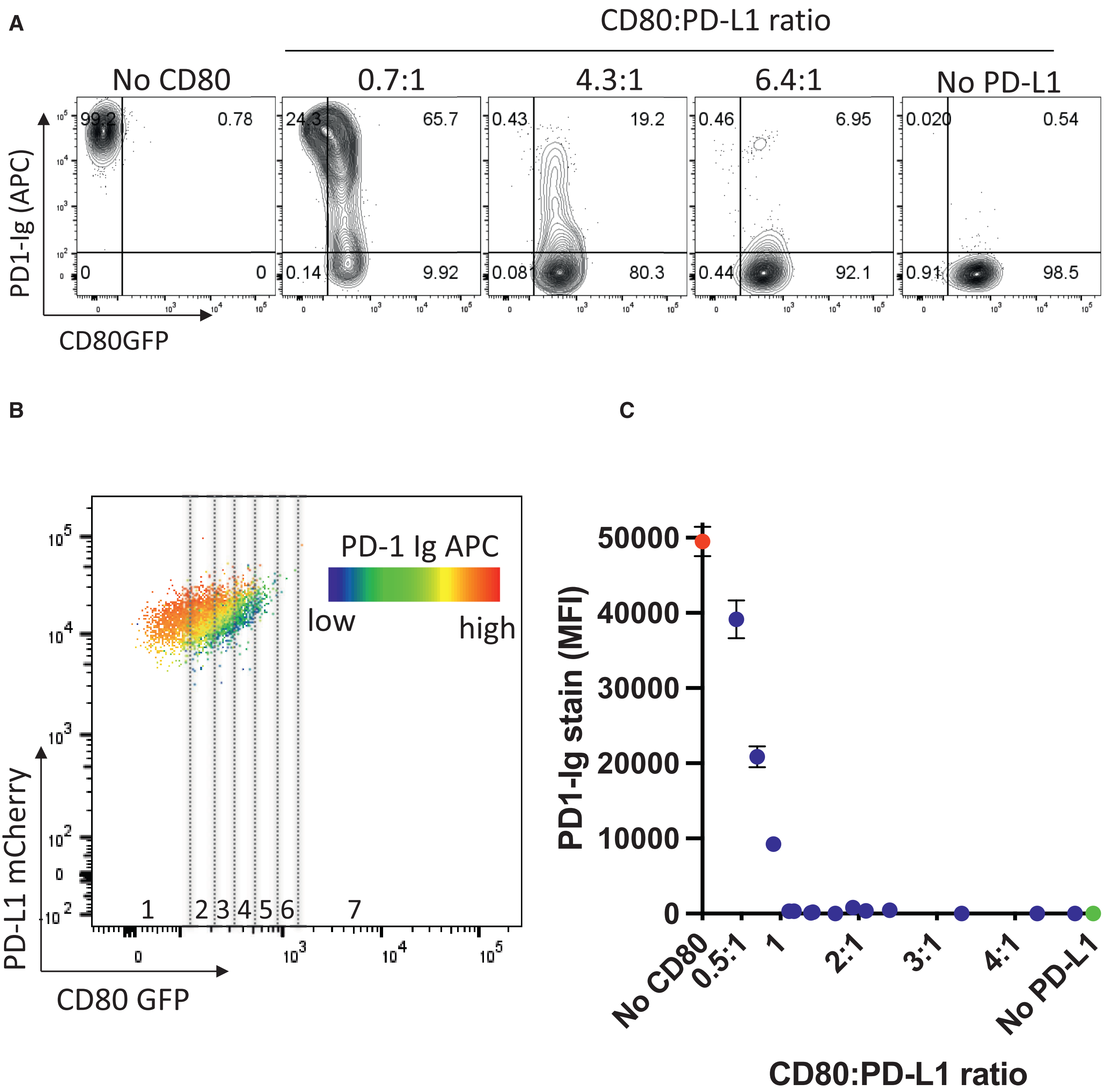

Figure 5. A 1:1 stoichiometry between PD-L1 and CD80 prevents PD-1 binding.

Molecular ratios of CD80GFP and PD-L1mCherry were determined using Quantum™ Simply Cellular® beads and ratios of PD-L1:CD80 calculated.

A Staining of free PD-L1 with APC-conjugated PD-1 Ig in DG-75 cells expressing only PD-L1mCherry, using cell lines with increasing ratios of CD80GFP: PD-L1mCherry (as calculated in EV4) compared to CD80GFP only. FACS plots are representative of four independent experiments.

B Representative FACS plot of a single DG-75 cell line co-expressing CD80GFP and PD-L1mCherry. Heatmap colours indicate levels of PD-L1 stained by PD-1 Ig as a function of CD80GFP expression. GFP levels of each cell line were gated into fractions (indicated by dotted lines) representing different GFP expression levels and the ratio of CD80:PD-L1 calculated and plotted against the respective APC-conjugated PD-1 Ig bound.

C Integrated data showing PD1-Ig detection as a function of the calculated ratio of CD80:PD-L1 (mean ± SEM, four independent experiments).

L1 (Fig EV5A) suggesting it does not necessarily displace PD-L1 on binding. Accordingly, using B cells expressing CD80/PD-L1 or CD80 alone, the dose–response curves of abatacept binding were not significantly different whether or not PD-L1 was present (Fig 8A and B), with an EC 50 of 0.25 µg/ml in the absence of PD-L1 and 0.29 µg/ml in the presence.

© 2023 The Authors

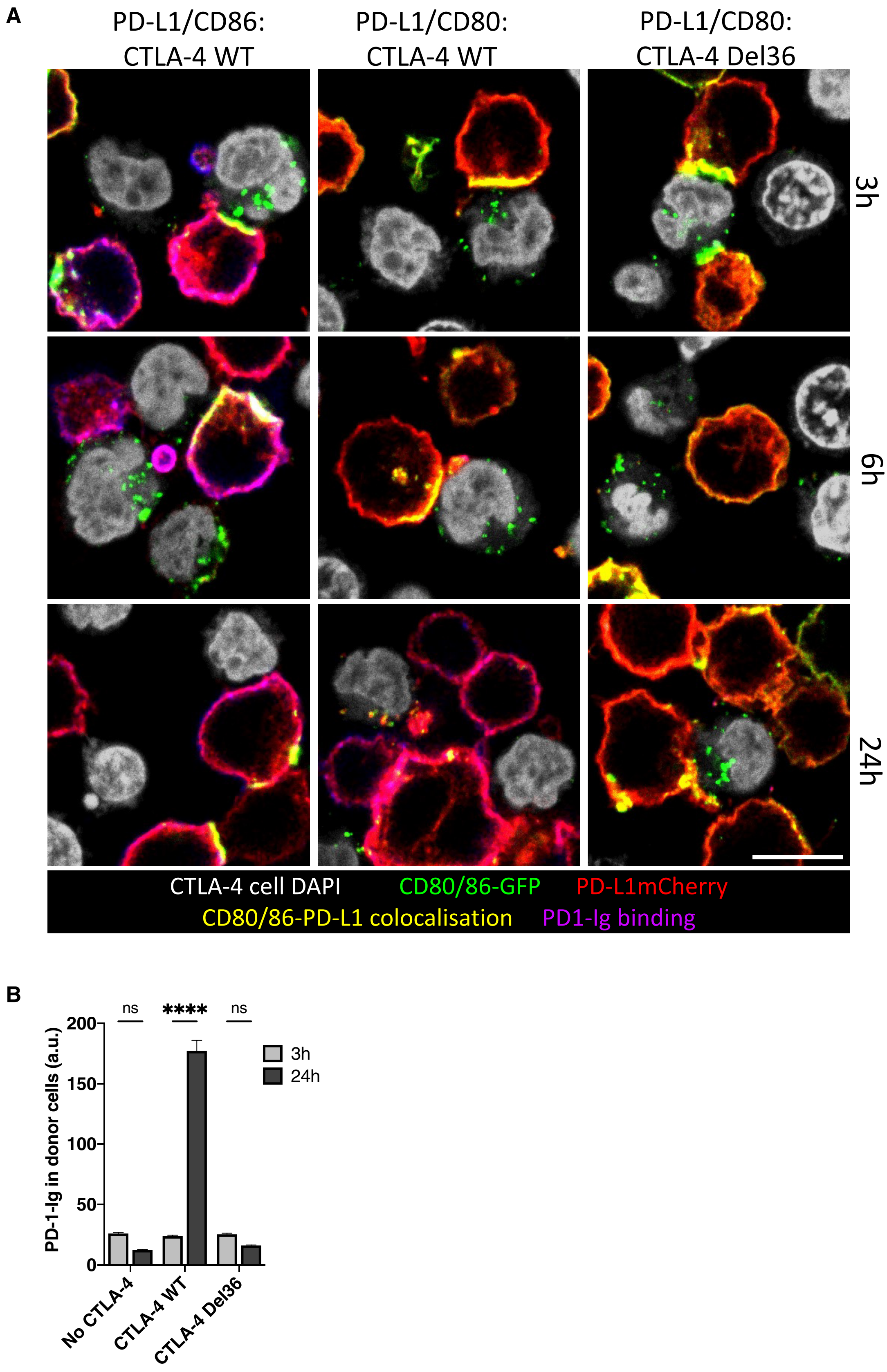

Figure 6.

 © 2023 The Authors

Figure 6. CD80/CD86 and PD-L1 are co-localised at the immune synapse during transendocytosis.

A DG-75 B cells expressing CD80GFP or CD86GFP (green) and PD-L1mCherry (red) were incubated with Jurkat cells (nuclei shown in grey) expressing CTLA-4 WT or mutant CTLA-4 Del36 for the indicated durations and analysed by confocal microscopy. Two hours prior to assay endpoint, APC-labelled PD-1 Ig (0.75 µg/ml) was added. CD80/CD86 and PD-L1 co-localisation at the immune synapse is shown in yellow with PD-1 Ig binding shown in purple. Scale bar, 10 µm.

B Fluorescence intensity of PD-1 Ig-APC binding to CD80/PD-L1 co-expressing B cells quantified after 3 and 24 h transendocytosis (mean ± SEM of a minimum of 44 cells per condition, $^{****}P \leq 0.0001$, ns, not significant: two-way ANOVA with Tukey's multiple comparisons test).

We also studied the impact of abatacept titrations on PD-L1 detection. In keeping with the observations of Tekguc *et al* (2021) we observed that abatacept enhanced the ability of two independent anti-PD-L1 antibodies to bind in a similar manner (Fig 8C and D). However, neither antibody achieved the maximal staining seen in the complete absence of CD80 or as observed following transendocytosis. Remarkably, when we attempted to detect PD-L1 with PD-1 Ig rather than antibody, we did not observe any impact of Abatacept (Fig 8C and D). Accordingly, PD-1-Ig staining remained inhibited by CD80, even when abatacept was bound at high concentrations. Furthermore, increasing abatacept incubation times did not substantially increase the detection by PD-1 Ig, indicating that free PD-L1 was not released significantly over time following abatacept binding (Fig 8E). In addition, we also tested the impact of a high affinity CTLA-4-Ig (belatacept), which performed no better than abatacept in terms of increasing PD-L1 staining (Fig EV5B–D), indicating that binding CTLA-4 molecules more strongly to CD80 does not liberate PD-L1 *per se*. Overall, these data highlight differences between the binding of anti-PD-L1 antibodies and PD-1 Ig and indicate CTLA-4 binding to CD80 alone is insufficient for effective release of PD-L1.

Discussion

The CTLA-4 and PD-1 pathways are critical regulators of T-cell immunity in the settings of autoimmunity and cancer immunotherapy (Schildberg *et al*, 2016; Ribas & Wolchok, 2018). Understanding the roles of these distinct pathways as well as their interactions is therefore increasingly important therapeutically. It is now emerging that these two pathways are functionally connected by an interaction between the CTLA-4 ligand, CD80 and the PD-1 ligand, PD-L1 when expressed on the same cell (Chaudhri *et al*, 2018; Sugiura *et al*, 2019). Thus, it is now important to understand the impact of this interaction on both the PD-1 and CTLA-4/CD28 pathways.

Previous data on the PD-L1:CD80 interaction offer conflicting evidence as to the nature, context and significance of this interaction. The interaction was initially reported and characterised using Fc–fusion proteins, which revealed CD80-Ig binding to PD-L1 expressing cells (Butte *et al*, 2007, 2008). Several further reports suggested that this "*trans*" intercellular interaction between ligand expressing cells resulted in inhibition of T cells responses via either CD80 or PD-L1 (Butte *et al*, 2007; Paterson *et al*, 2011; Ni *et al*, 2017; Cassady *et al*, 2018). However, more recently it has emerged that the interaction between CD80 and PD-L1 in the intercellular "*trans*" conformation is weak and likely occurs in "*cis*" where CD80 and PD-L1 are expressed on the same membrane. Alternatively, "trans" interactions can occur where there is significant flexibility for binding, such as when Ig-fusion proteins are used in specific configurations (Chaudhri *et al*, 2018) (Sugiura *et al*, 2019). Our results show that CD80-Ig stained PD-L1 expressing cells very poorly, whereas in accordance with data from others we detected a robust CD80:PD-L1 *cis*-interaction when both proteins are expressed on the same cell. This *cis* CD80:PD-L1 interaction precluded PD-L1 binding to PD-1 in two independent cell types (CHO and B cells). In line with the data from Sugiura *et al* (2019) we also found that CD80 forms heterodimers with PD-L1, which can be co-precipitated upon crosslinking, supporting a significant interaction between the two partners.

Structural data indicate that the heterodimeric interaction between CD80 and PD-L1 obscures the PD-1 binding site, but leaves the overlapping CD28 and CTLA-4 binding sites, which occur via the opposite (AGFCC'C") face of CD80 (Sugiura *et al*, 2019) available. Interestingly the PD-1:PD-L1 interactions are unusual in structure and similar to interactions between V_H and V_L domains of an antibody, generating a "cheek to cheek" interaction (Freeman, 2008; Lin *et al*, 2008). It is the location of this binding site that appears to be obscured by CD80 when it forms a heterodimer with PD-L1. In contrast, for CD80 this interaction occurs at its normal dimerization interface allowing the formation of CD80-PD-L1 heterodimers, which are on the opposite face to the CD28/CTLA-4 binding site (Stamper *et al*, 2001). Accordingly, we saw no obvious effect on binding of CTLA-4 and CD28 as others have reported (Sugiura

Figure 7. CTLA-4 mediated trans-endocytosis of CD80 rescues PD-L1:PD-1 inhibition.

A Schematic of an *in vitro* assay to monitor CD80 transendocytosis and subsequent PD-1 mediated inhibition. CTFR-stained DG-75 cells expressing CD80 and PD-L1 were loaded with 200 ng/ml of SEE superantigen and co-incubated for 24 h with WT CTLA-4$^+$ Jurkat T-cells (J_{reg}) or control CTLA-4$^-$ Jurkat cells. CTV-stained PD-1+ve Jurkat T-cells were used as responder cells to measure functional PD-L1-PD-1 interactions.

B Flow cytometry plots of CD80GFP levels on DG-75 B-cells (gated on CTFR$^+$ cells) at 24 h with graphical representation of the MFI in each condition (RH graph). The J_{reg} cells used, and the anti-PD-1 status is indicated above the plot.

C Flow cytometry plots of CD28 levels indicating contact between the CD80$^+$ APC and the PD-1$^+$ CD28$^+$ responder Jurkat T-cells, following CD80 transendocytosis. Graphical representation of the CD28 MFI in each condition is shown on the right.

D Flow cytometry of CD69 levels on responder Jurkat T-cells and graphical representation of CD69 MFI in each condition.

E Flow cytometry of CD69 levels on CD28KO (PD-1$^+$) responder Jurkat T-cells and graphical representation of the CD69$^+$ cells MFI in each condition. Cartoons on the left of (B–E) illustrate the cell type analysed.

F As in (E), but showing MFI of CD69$^+$ cells on responder Jurkat T-cells with titration of SEE superantigen.

Data information: Data are representative of three independent experiments showing mean ± SEM. $^*P \leq 0.05$, $^{**}P \leq 0.01$, ns, not significant: two-way ANOVA with Tukey's multiple comparisons test.

© 2023 The Authors

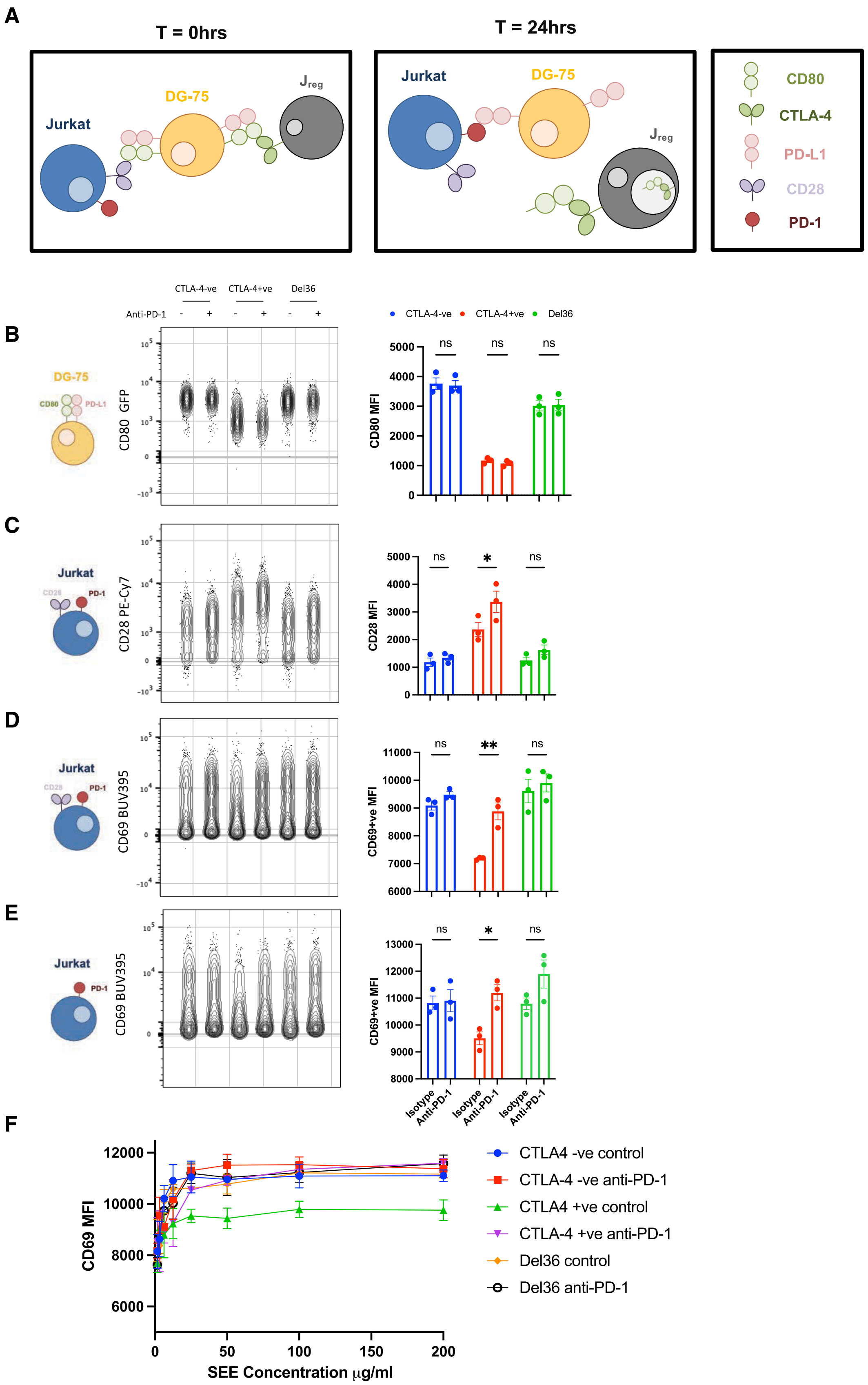

Figure 7.

 © 2023 The Authors

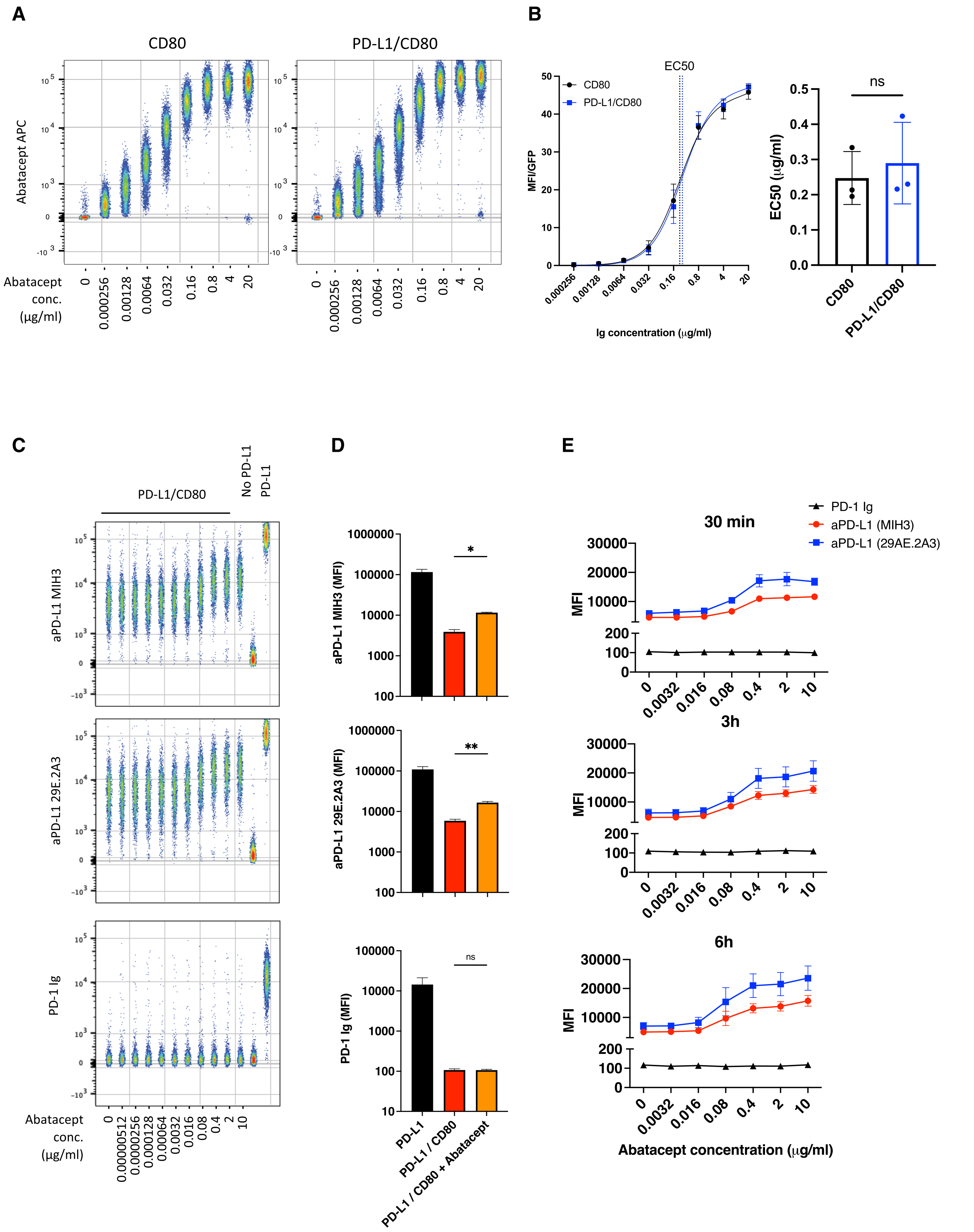
A
CD80
PD-L1/CD80
Abatacept APC
Abatacept conc. (µg/ml)
0
0.000256
0.00128
0.0064
0.032
0.16
0.8
4
20
B
EC50
CD80
PD-L1/CD80
MFI/GFP
Ig concentration (µg/ml)
ns
EC50 (µg/ml)
C
PD-L1/CD80
No PD-L1
PD-L1
aPD-L1 MIH3
aPD-L1 29E.2A3
PD-1 Ig
Abatacept conc. (µg/ml)
0
0.00000512
0.0000256
0.000128
0.00064
0.0032
0.016
0.08
0.4
2
10
D
aPD-L1 MIH3 (MFI)
aPD-L1 29E.2A3 (MFI)
PD-1 Ig (MFI)
*
**
ns
PD-L1
PD-L1 / CD80
PD-L1 / CD80 + Abatacept
E
30 min
3h
6h
PD-1 Ig
aPD-L1 (MIH3)
aPD-L1 (29AE.2A3)
MFI
Abatacept concentration (µg/ml)

Figure 8.

Figure 8. CTLA-4 Ig (Abatacept) modulates anti-PD-L1 antibody binding but fails to confer PD-1 Ig detection.

A Concatenated flow cytometry plots for Abatacept-APC binding to DG-75 cells expressing CD80 or PD-L1/CD80.
B Titration of Abatacept staining (left hand panel) and EC50 values of Abatacept (right hand panel) for CD80 only or PD-L1/CD80 co-expressing DG-75.
C Concatenated plot of a one in five serial dilution of Abatacept (starting at 10 μg/ml) followed by PD-L1 detection using antibody (MIH3 or 29E.2A3 clone) or using PD-1 Ig, all at 1 μg/ml. DG-75 with no ligand or PD-L1 alone (far right) are shown as staining controls in the presence of 10 μg/ml of abatacept.
D Comparison of PD-L1 detection using PD-1-Ig or anti-PD-L1 antibodies (MIH3 or 29E.2A3 clone) with and without prior incubation with 10 μg/ml of abatacept, based on data from (C).
E Graphs showing PD-L1 detection under conditions used in (C) following different Abatacept incubation periods (30 min, 3 h or 6 h).

Data information: Data are representative of three independent experiments showing mean ± SEM. *$P \leq 0.05$, **$P \leq 0.01$, ns, not significant: paired t-test (B) or RM one-way ANOVA (D).

et al, 2019). These data are somewhat in contrast to the suggestion of disrupted CD80:CTLA-4 interaction reported by Zhao *et al* (2019). This study concluded that transendocytosis of CD80 was impaired. However, our data show continued and effective binding of CTLA-4 to CD80-PD-L1 heterodimers, which allows transendocytosis to proceed unimpaired, resulting in robust and continuous removal of CD80 in a time dependent manner. The reason for the differences between our observations currently remains unclear but may relate to differences in how transendocytosis was measured. For example, our assays tracked the loss and uptake of tagged ligands, whereas those of Zhao utilised anti-CD80 to detect residual CD80 on Raji cells. The latter approach is subject to blocking effects by, for example, soluble or cleaved CTLA-4 molecules. Alternatively, there may be differences between the B cell APCs used, for example in expression of CMTM6, which associates with PD-L1 (Burr *et al*, 2017; Mezzadra *et al*, 2017) and might affect these results. Nonetheless, using our system we have not observed inhibitory effects on CD80 transendocytosis resulting from co-expression of PD-L1.

Our data further show that the outcome of CTLA-4 transendocytosis of CD80 is the liberation of PD-L1 that binds to PD-1, highlighting that CD80 is a potential regulator of the PD-1 pathway. Accordingly, in the context of PD-L1 co-expression, CTLA-4-mediated depletion of CD80 regulates PD-L1 availability, thus rescuing PD-1 receptor binding—in this context CTLA-4 becomes a regulator of PD-1 signalling. These data are consistent with those from Sugiura *et al* (2019), who showed that the level of available PD-L1 was related to the expression level of CD80 on different immune cell types. These concepts therefore have interesting implications for immunotherapy, where combinations of CTLA-4 and PD-L1 or PD-1 antibodies are used. For example, anti-CTLA-4 treatment would be predicted to increase levels of CD80 (by blocking transendocytosis), thereby potentiating CD80:PD-L1 interactions, effectively inhibiting PD-1 functions controlled by PD-L1.

Further data have suggested that CTLA-4 proteins lacking the cytoplasmic domain can effectively reveal PD-L1 after removing CD80 (Tekguc *et al*, 2021), in a process more akin to trogocytosis (Auscher *et al*, 2008; Daubeuf *et al*, 2010). Indeed, we also find that CTLA-4 when highly expressed at the cell surface (due to cytoplasmic domain deletion) is capable of trogocytosis, with CTLA-4 Del36 capturing relatively small amounts of CD80 as seen in both flow cytometric and microscopy assays. However, it is important to recognise that the tailless CTLA-4 Del36 mutant is highly overexpressed at the plasma membrane compared to WT CTLA-4, thereby facilitating trogocytosis. In our experience, trogocytosis is observed for many proteins following the physical disruption of cell conjugates that is used in order to measure protein transfer between cells using flow cytometry. In such settings, one concern is that proteins get transferred as a result of this physical separation, thereby possibly overestimating the amount of protein actually captured naturally. In contrast, transendocytosis robustly depletes CTLA-4 ligands *in situ*, as observed by confocal microscopy, without requiring cell separation (Qureshi *et al*, 2011). Accordingly, using confocal microscopy to analyse cells in contact, we did not observe robust detection of PD-L1 following trogocytosis. Moreover, a hallmark of transendocytosis is that it is a time sensitive process where transfer of ligands is ongoing during cell–cell contacts. This can result in the almost complete depletion of CD80 or CD86 given sufficient time. We observed here that the cytoplasmic domain of CTLA-4 was critical to this efficient time-dependent ligand transfer, with WT CTLA-4 significantly outperforming the Del36 cytoplasmic mutant. Therefore, whilst it is clear that high levels of surface CTLA-4 can capture significant quantities of CD80 by trogocytosis, the physiological importance of this compared to the transendocytosis process remains unclear. It is also important to note that the CTLA-4 cytoplasmic domain is highly conserved through mammalian evolution, suggesting it has important conserved functions that are likely to be involved in protein trafficking (Walker & Sansom, 2011; Lo *et al*, 2015; Hou *et al*, 2017). Since there are no reported mutations in the CTLA-4 cytoplasmic domain in humans despite numerous CTLA-4 mutations being reported clinically (Schwab *et al*, 2018), this attests to the importance of this region for normal CTLA-4 function and indicates that a tailless CTLA-4 molecule is not physiologically viable in humans.

A further observation from our work is the remarkable selectivity of transendocytosis. Whilst trogocytosis is generally nonselective since other proteins are co-transferred during these assays (Auscher *et al*, 2008; Daubeuf *et al*, 2010; Tekguc *et al*, 2021), we found transendocytosis extremely selective in our assays. Specifically, both CD80 and CD86 were depleted from B cells and CHO cells, whilst leaving PD-L1 on the donor cell membrane. This selectivity is not cell-type dependent, or due to the nature of the cell contact, but seems due to the nature of transendocytosis itself. This is all the more remarkable for CD80:PD-L1, where despite being part of a heterodimer, only CD80 is specifically depleted, whilst PD-L1 remains. Accordingly, CD80:PD-L1 heterodimers must separate prior to transendocytosis, although this is not induced *per se* by simple CTLA-4 binding. Interestingly, CD80 binds more avidly to PD-L1 than it does to itself, with interaction affinity between CD80 and PD-L1 in the low micromolar range (Butte *et al*, 2007, 2008; Cheng *et al*, 2013). This is somewhat stronger than the interaction between CD80 monomers, which form non-covalent homodimers with ~ 50 μM affinity

 © 2023 The Authors

(Ikemizu *et al*, 2000). Nonetheless, the 0.2 μM affinity between CTLA-4 and CD80 (Collins *et al*, 2002) is still highly favoured, presumably allowing CTLA-4 to specifically deplete free CD80 molecules, which may arise during normal dissociation of these non-covalent homo- and hetero- dimers.

A further significant observation from our work is that CTLA-4 binding by abatacept did not appear to dissociate the PD-L1:CD80 heterodimer effectively, such that PD-1 can bind. Our data show that soluble abatacept binding to CD80 is unimpeded by co-expression of PD-L1, consistent with the location of its binding site relative to PD-L1 binding (Stamper *et al*, 2001; Sugiura *et al*, 2019) Strikingly, whilst abatacept did affect staining by anti-PD-L1 antibodies, it was unable to recover detection by PD-1 Ig. Given that PD-1 Ig can readily stain free PD-L1, this suggests that abatacept does not readily generate free PD-L1. One possibility is that binding of abatacept alters the conformation of the heterodimer, such that high affinity antibodies can bind. Alternatively, there may be steric issues that arise in particular cell types or at specific expression ratios that allow CD80-PD-L1 binding to be disrupted by abatacept. However, in our experiments binding of abatacept and its higher affinity relative belatacept, was insufficient for liberation of PD-L1. Given the use of abatacept clinically to treat autoimmunity and LRBA deficiency (Chitale & Moots, 2008; Lo *et al*, 2015), understanding its impact on the PD-1 pathway is of some significance. Our data indicate that effective depletion of CD80 rather than simple binding of CTLA-4 is required to liberate functional PD-L1. Such a requirement for CD80 depletion is a strong fit with the transendocytosis process itself, which is a major feature of CTLA-4 biology.

The functional significance of the CD80:PD-L1 interaction is seen *in vivo* whereby CD80 or PD-L1 mutants lacking the ability to interact show attenuated immune responses due to excess engagement of PD-1, thereby limiting tumour immunity (Sugiura *et al*, 2019). This places CD80 as an accelerator or “turbocharger” of T-cell responses by inhibiting PD-L1:PD-1 function. We would suggest that CD80 has similar functions in the CD28/CTLA-4 pathway, whereby its dominant binding to CTLA-4 could protect CD86 from transendocytosis. Together these data raise the possibility that CD80 itself operates as a switch, promoting enhanced T-cell responses by inhibiting both CTLA-4 and PD-1 pathways.

Materials and Methods

Tissue culture and cell lines

All cell lines were maintained at 37°C, 5% CO_2 in a humidified atmosphere. Jurkat E6.1T cells (ATCC, TIB-152) and DG-75 B cells (ATCC, CRL-2625) were grown in complete RPMI 1640 media supplemented with 10% FBS, 2 mM L-Glutamine, 100 U/ml penicillin and 100 mg/ml streptomycin (all from Life Technologies, Gibco).

Chinese Hamster Ovary (CHO-K1) adherent cells (ATCC, CCL-61) were maintained in complete DMEM media (Dulbecco’s Modified Eagle Medium supplemented with 10% FBS, 2 mM L-Glutamine, 100 U/ml penicillin and 100 mg/ml streptomycin—all from Life Technologies, Gibco). Cells were routinely detached with trypsin EDTA and passaged 1 in 10.

Cell line engineering

Transduced cell lines expressed stable integrations of transgenes using human CTLA-4, CTLA-4 Del36, PD-L1, CD80 or CD86 tagged fusion proteins cloned into the MP71 retroviral vector. CTLA-4 Del36 was generated by introduction of a stop codon prior to the 36 amino acids comprising the cytoplasmic domain after lysine 188. Phoenix-Amphoteric packaging cells were transfected with MP71 constructs in combination with pVSV using FUGENE HD transfection reagent (Roche Molecular Biochemical) to obtain retroviral supernatants, which were harvested 24 h post-transfection and used to transduce CHO, Jurkat (CTLA-4) or DG-75 (CD80, CD86, PD-L1) cell lines. Endogenous CD80 and CD86 were initially knocked out from DG-75 cells using CRISPR/Cas9. Ribonucleoprotein complexes composed of 2 μg Cas9 protein (TrueCut™ Cas9 Protein v2) and 500 ng sgRNA (CD80 target: TTGAGGTATGGACACTTGGA; CD86 target sequence: GTAACCGTGTATAGATGAGC) were electroporated into 2×10^5 target cells using the Neon™ Transfection System (Thermo Fisher Scientific). Following recovery, cell populations were sorted, and endogenous ligand-negative cells were selected for further transduction with tagged fusion protein constructs. Fusion protein constructs were generated by the addition of a C terminal GFP (or mCherry) directly following a mutated stop codon. For transduction, non-tissue culture treated 24-well plates were coated with RetroNectin (TaKaRa) overnight at 30 mg/ml. 5×10^5 cells were added to 1 ml of retroviral supernatants in the RetroNectin pre-coated wells and centrifuged at 2000 rpm at 32°C for 2 h. Media was changed to fresh media appropriate for each cell type 24 h post-infection. Three days post-transduction, cells were stained for transduced protein expression and analysed by flow cytometry.

Flow cytometry

Antibodies and detection reagents

APC-conjugated proteins: PD-1-Ig (Bio-Techne, 1086-PD-050), anti-PD-L1 (Durvalumab) and CTLA-4 Ig (Abatacept & Belatacept) were generated using the APC Conjugation Kit—Lightning-Link® according to manufacturer’s instructions (Abcam, ab201807). APC-conjugated anti-PD-L1 antibodies were procured from ThermoFisher (clone MIH1 [#14-5983-82]), or Biolegend (clones MIH3 [#374513] and 29E.2A3 [#329707]). Anti CD28-APC was procured from BD Biosciences (#559770).

Transendocytosis assays

Ligand donor cells (CHO or DG-75 B cells) expressed CD80 or CD86 molecules C-terminally tagged with GFP and/or PD-L1 molecules C-terminally tagged with mCherry. Donor cells were labelled with CellTrace Violet (CTV) labelling kit (ThermoFisher Scientific) according to the manufacturer’s instructions. CHO or Jurkat cells expressing no CTLA-4, CTLA-4 WT or Del36 were used as recipient cells. Donor and recipient cells were plated in round-bottom 96-well plates at 37°C at the ratios of donor:recipient cells and incubation times indicated in the figure legends. 20 ng/ml *Staphylococcus aureus* Enterotoxin type E (Cusabio, CSB-YP320170FKZ) was added to Jurkat: DG-75 transendocytosis assays to provide TCR stimulation. Where CD28 downregualtion was investigated, DG-75 and Jurkat cells were combined as detailed above for 3 h prior to staining for surface CD28 levels on the Jurkat recipient cells.

© 2023 The Authors

Where the ability of transendocytosis of CD80 to release PD-L1 was tested, 750 ng/ml PD-1 Ig-APC or 500 ng/ml 29E.2A3 were added to the transendocytosis setup 2 h prior to the assay endpoint. After the indicated transendocytosis duration, cells were washed three times with ice-cold PBS, fixed in 4% ice-cold paraformaldehyde in PBS for 5 min on ice and another 15 min at room temperature. Cells were analysed by flow cytometry, gating on singlets and GFP, mCherry or APC fluorescence. Ligand loss from donor cells (CTV^+) was calculated as percentage of donor ligand remaining (MFI) relative to the control expression following incubation with no CTLA-4. Using the formula: ligand MFI after TE/ligand MFI with no CTLA-4 × 100.

Flow cytometry analysis

CHO cells expressing PD-L1 alone, or co-expressing CD80GFP or CD86GFP were stained with CD80-Ig, PD-1-Ig, CTLA-4 Ig (Abatacept) or a-PD-L1 clones MIH1, MIH3 or 29E.2A3 at 37°C. Antibodies were typically used at 2 μg/ml unless otherwise stated and secondary stained for anti-human IgG-PE. CHO cells expressing CTLA-4 WT or CTLA-4 Del36 were surface CTLA-4 stained on ice with anti-CTLA4-PE (BD Biosciences, clone BNI3) for 1 h. Cells were washed three times with ice-cold PBS, fixed with ice-cold 4% PFA in PBS for 5 min on ice and another 15 min at room temperature and analysed by flow cytometry gating on single cells.

Where the ability of soluble CTLA-4 (Abatacept or Belatacept) to release PD-L1 from the CD80/PD-L1 heterodimer was tested, cells were incubated for 0.5, 3 and 6 h with a titration of Abatacept or Belatacept (as indicated in figures) at 37°C. Cells were washed three times and stained with either 1 μg/ml PD-1 Ig-APC or anti-PD-L1 (clones MIH3 or 29E.2A3) for 30 min at 37°C. Cells were washed three times prior to analysis by flow cytometry.

PD-1 function assays

1×10^5 CTFR-stained DG-75 B-cells expressing PD-L1-mCherry/CD80GFP, were mixed with 0.5×10^5 CTV-stained PD-1 + ve Jurkat T-cells and 0.5×10^5 Jurkat T-cells (either CTLA-4^{-ve}, CTLA-4^{+ve} ('Jreg)' or CTLA-4-Del36 mutant) in round-bottom 96-well plates. Cells were supplemented with SEE superantigen at indicated concentrations. 5 μg/ml of blocking anti-PD-1 antibody or hIgG1 isotype antibody (both provided by AstraZeneca) were added to monitor PD-1 mediated effects upon activation. Cells were incubated for 24 h at 37°C followed by washing in PBS prior to staining for CD69 (FN50, BD Biosciences #564364) & CD28 (CD28.2, BD Biosciences, #560684) for 30 min at 37°C. Cells were washed three times followed by 4% PFA treatment prior to analysis by flow cytometry.

Confocal microscopy

Transendocytosis assays were performed as described for flow cytometry at a ratio of 1:1 Jurkat:DG-75 cells. Jurkat cells expressing no CTLA-4, CTLA-4 WT or CTLA-4 Del36 were labelled with CTV and incubated with CD80GFP, CD86GFP and/or PD-L1mCherry expressing DG-75 cells in round-bottom 96-well plates at 37°C at a 1:1 ratio Jurkat:DG-75 in the presence of 20 ng/ml *Staphylococcus aureus* Enterotoxin type E for the indicated time points to permit transendocytosis. 750 ng/ml APC-conjugated PD-1 Ig was added 2 h prior to transendocytosis endpoint. Cells were then washed twice in ice-cold PBS and resuspended in cold PBS. 2×10^5 cells were transferred into a well of a 0.01% Poly-L-lysine (Sigma Aldrich)-coated 96 well plate (Greiner Screenstar) on ice. Ice-cold 8% paraformaldehyde in PBS was added 1:1 v/v and cells were fixed onto the well bottom by centrifuging at room temperature for 20 min at 500 *g*. Following sequential washes of 2% FBS in PBS, PBS and 0.1% Saponin, cells were stained with 2 μg/ml DAPI for 45 min at room temperature in the dark. After sequential 0.1% Saponin, PBS and deionised water washes, cells were mounted in Mowiol with 2.5% DABCO.

All confocal data were acquired on an inverted Nikon Eclipse Ti equipped with a 60× oil immersion objective. Constant laser powers and acquisition parameters were maintained throughout. Digital images and scale bars were prepared using Fiji. Quantitation was performed using CellProfiler 2.2.0 analysis software.

Biochemistry

For crosslinking, DG-75 cells expressing CD80GFP, PD-L1mCherry, CD80GFP/PD-L1mCherry and CD86GFP/PD-L1mCherry were washed three times in PBS and resuspended at 25×10^6 cells/ml in 1 mM of BS3 crosslinker (ThermoFisher, cat. # A39266). Cells were incubated at room temperature for 30 min followed by quenching with RPMI for 15 min; cells were then washed three times in PBS.

Cell lysates were prepared in lysis buffer containing 50 mM Tris–HCl, pH 7.4, 50 mM NaCl, 10 mM EDTA and 1% Triton x-100 supplemented with Protease Inhibitor Cocktail (cat. Number: 5871, Cell Signalling Technology). After 30 min incubation at 4°C with gentle rotation, lysates were spun at 10,000 *g* for 10 min. Supernatants were denatured at 100°C and processed with the Bio-Rad MiniProtean Tetra Cell Gel Electrophoresis system. Gels were transferred to PVDF membranes and blocked for 1 h at room temperature in TBST supplemented with 5% milk. Western blotting was performed overnight at 4°C with the following antibodies: anti-GFP (Chromotek cat. # 3H9), anti-RFP (Chromotek cat. # 6G6). Blots were washed three times in TBST before secondary staining with HRP-linked anti-Mouse (Cell Signalling Technology #7076) at room temperature for 45 min. After incubation, blots were washed three times in TBST. To visualise the bands, blots were incubated for 5 min in Bio-Rad Clarity ECL substrate; blots were acquired on Bio-Rad Chemidoc.

For co-immunoprecipitation, supernatants from cell lysates were processed with an RFP-trap (Chromotek) or with Dynabeads protein A/protein G (ThermoFisher) combined with Abatacept. Beads and lysates were incubated for 1 h at 4°C with gentle rotation and subsequently washed three times in PBST. Immuno-precipitates were resolved by Western Blot as described for cell lysates.

Calculation of CD80:PD-L1 Ratios on DG-75 cell lines

Levels of CD80 and PD-L1 on DG-75 cells expressing either CD80GFP or PD-L1mCherry were calculated using Quantum™ Simply Cellular® beads (Bangs Laboratories Inc., #815) according to manufacturer's instructions. Saturating concentrations of anti-CD80 Alexa Fluor 700 (clone L307.4, BD Biosciences, # 561133, 5 μg/ml), anti-PD-L1 APC clone MIH3 (Biolegend, #374514, 25 μg/ml) and clone 29E.2A3 (Biolegend, #329708, 25 μg/ml) were determined. Cells were washed with PBS and stained separately at room temperature with each antibody diluted in PBS containing 2% FBS for

 © 2023 The Authors

30 min. The same staining protocol was applied in parallel to Quantum™ Simply Cellular® beads. Cells and beads were washed three times with PBS and analysed by flow cytometry.

The MFI measured in the different bead populations was used to fit a calibration curve in the QuickCal® Excel template provided by Bang's Laboratories, plotting the MFI against the beads' antibody-binding capacity (ABC) as specified by the manufacturer. Based on the calibration curve, the number of CD80GFP and PD-L1mCherry molecules in DG-75 cell lines expressing different levels of each ligand were determined. The corresponding GFP or mCherry MFIs were used to calculate CD80GFP or PD-L1mCherry levels on cell lines expressing both ligands, which were analysed using the same instrument settings. To determine the CD80:PD-L1 ratio at which free PD-L1 is available for PD-1 binding, these cell lines were washed with PBS, stained with 1.5 μg/ml APC-conjugated PD-1 Ig for 1 h at 37°C and washed three times with PBS prior to analysis by FACS. GFP levels were split into seven bins and the respective CD80:PD-L1 ratio calculated to increase resolution for plotting a curve representing free PD-L1 detected by PD-1 Ig stain depending on CD80:PD-L1 ratios.

Statistical analysis

Statistical analyses and significance were determined using GraphPad Prism v9.02 software (GraphPad Software Inc.). All analyses were performed in triplicate or greater and the means obtained were used for independent *t*-tests or two-way ANOVA with Tukey's correction for multiple comparisons unless otherwise stated. No statistical methods were used to pre-determine sample sizes. Data distribution was assumed to be normal, but this was not formally tested. Data collection and analysis were not performed blind to the conditions of the experiments.

Data availability

This study includes no data deposited in external repositories.

Expanded View for this article is available online.

Acknowledgements

AK, EW, CH, NH and DMS were funded by the Wellcome Trust (Grants 204798/Z/16/Z, and 110297). MR was funded by an MRC industrial collaborative studentship with Astra Zeneca. CW was funded by Versus Arthritis (Grant 21147). This research was funded in whole, or in part, by the Wellcome Trust (Grant 204798/Z/16/Z). For the purpose of Open Access, the author has applied a CC BY public copyright licence to any Author Accepted Manuscript version arising from this submission.

Author contributions

Alan Kennedy: Data curation; formal analysis; investigation; methodology; writing—review and editing. **Max Robinson:** Data curation; formal analysis; investigation; methodology; writing—review and editing. **Claudia Hinze:** Data curation; formal analysis; investigation; methodology; writing—review and editing. **Erin Waters:** Data curation; investigation; methodology. **Cayman Williams:** Data curation; investigation; methodology. **Neil Halliday:** Data curation; investigation; methodology. **Simon Dovedi:** Supervision; funding acquisition; writing—review and editing. **David M Sansom:** Conceptualization; data curation; funding acquisition; investigation; methodology; writing—original draft; writing—review and editing.

Disclosure and competing interests statement

SJD is full time employee and shareholder in AstraZeneca. MR received funding from AstraZeneca in support of this work.

References

Auscher A, Magdeleine E, Joly E, Hudrisier D (2008) Capture of plasma membrane fragments from target cells by trogocytosis requires signaling in T cells but not in B cells. *Blood* 111: 5621–5628

Burr ML, Sparbier CE, Chan YC, Williamson JC, Woods K, Beavis PA, Lam EYN, Henderson MA, Bell CC, Stolzenburg S *et al* (2017) CMTM6 maintains the expression of PD-L1 and regulates anti-tumour immunity. *Nature* 549: 101–105

Butte MJ, Keir ME, Phamduy TB, Sharpe AH, Freeman GJ (2007) Programmed death-1 ligand 1 interacts specifically with the B7-1 costimulatory molecule to inhibit T cell responses. *Immunity* 27: 111–122

Butte MJ, Pena-Cruz V, Kim MJ, Freeman GJ, Sharpe AH (2008) Interaction of human PD-L1 and B7-1. *Mol Immunol* 45: 3567–3572

Cassady K, Martin PJ, Zeng D (2018) Regulation of GVHD and GVL activity via PD-L1 interaction with PD-1 and CD80. *Front Immunol* 9: 3061

Chaudhri A, Xiao Y, Klee AN, Wang X, Zhu B, Freeman GJ (2018) PD-L1 binds to B7-1 only In cis on the same cell surface. *Cancer Immunol Res* 6: 921–929

Cheng X, Veverka V, Radhakrishnan A, Waters LC, Muskett FW, Morgan SH, Huo J, Yu C, Evans EJ, Leslie AJ *et al* (2013) Structure and interactions of the human programmed cell death 1 receptor. *J Biol Chem* 288: 11771–11785

Chitale S, Moots R (2008) Abatacept: the first T lymphocyte co-stimulation modulator, for the treatment of rheumatoid arthritis. *Expert Opin Biol Ther* 8: 115–122

Collins A, Brodie D, Gilbert R, Iaboni A, Manso-Sancho R, Walse B, Stuart D, van der Merwe P, Davis S (2002) The interaction properties of costimulatory molecules revisited. *Immunity* 17: 201–210

Daubeuf S, Aucher A, Bordier C, Salles A, Serre L, Gaibelet G, Faye JC, Favre G, Joly E, Hudrisier D (2010) Preferential transfer of certain plasma membrane proteins onto T and B cells by trogocytosis. *PLoS One* 5: e8716

Freeman GJ (2008) Structures of PD-1 with its ligands: sideways and dancing cheek to cheek. *Proc Natl Acad Sci USA* 105: 10275–10276

Freeman GJ, Long AJ, Iwai Y, Bourque K, Chernova T, Nishimura H, Fitz LJ, Malenkovich N, Okazaki T, Byrne MC *et al* (2000) Engagement of the PD-1 immunoinhibitory receptor by a novel B7 family member leads to negative regulation of lymphocyte activation. *J Exp Med* 192: 1027–1034

Garrett-Thomson SC, Massimi A, Fedorov EV, Bonanno JB, Scandiuzzi L, Hillerich B, Seidel RD 3rd, Love JD, Garforth SJ, Guha C *et al* (2020) Mechanistic dissection of the PD-L1:B7-1 co-inhibitory immune complex. *PLoS One* 15: e0233578

Halliday N, Williams C, Kennedy A, Waters E, Pesenacker AM, Soskic B, Hinze C, Hou TZ, Rowshanravan B, Janman D *et al* (2020) CD86 is a selective CD28 ligand supporting FoxP3+ regulatory T cell homeostasis in the presence of high levels of CTLA-4. *Front Immunol* 11: 600000

Hou TZ, Qureshi O, Wang CJ, Baker J, Young SP, Walker LS, Sansom DM (2015) A Transendocytosis model of CTLA-4 function predicts its suppressive behaviour on regulatory T cells. *J Immunol* 194: 2148–2159

Hou TZ, Verma N, Wanders J, Kennedy A, Soskic B, Janman D, Halliday N, Rowshanravan B, Worth A, Qasim W *et al* (2017) Identifying functional

defects in patients with immune dysregulation due to LRBA and CTLA-4 mutations. *Blood* 129: 1458–1468

Hui E, Cheung J, Zhu J, Su X, Taylor MJ, Wallweber HA, Sasmal DK, Huang J, Kim JM, Mellman I *et al* (2017) T cell costimulatory receptor CD28 is a primary target for PD-1-mediated inhibition. *Science* 355: 1428–1433

Ikemizu S, Gilbert RJ, Fennelly JA, Collins AV, Harlos K, Jones EY, Stuart DI, Davis SJ (2000) Structure and dimerization of a soluble form of B7-1. *Immunity* 12: 51–60

Kallies A, Zehn D, Utzschneider DT (2020) Precursor exhausted T cells: key to successful immunotherapy? *Nat Rev Immunol* 20: 128–136

Kamphorst AO, Wieland A, Nasti T, Yang S, Zhang R, Barber DL, Konieczny BT, Daugherty CZ, Koenig L, Yu K *et al* (2017) Rescue of exhausted CD8 T cells by PD-1-targeted therapies is CD28-dependent. *Science* 355: 1423–1427

Kennedy A, Waters E, Rowshanravan B, Hinze C, Williams C, Janman D, Fox TA, Booth C, Pesenacker AM, Halliday N *et al* (2022) Differences in CD80 and CD86 transendocytosis reveal CD86 as a key target for CTLA-4 immune regulation. *Nat Immunol* 23: 1365–1378

Khailaie S, Rowshanravan B, Robert PA, Waters E, Halliday N, Badillo Herrera JD, Walker LSK, Sansom DM, Meyer-Hermann M (2018) Characterization of CTLA4 trafficking and implications for its function. *Biophys J* 115: 1330–1343

Kuehn HS, Ouyang W, Lo B, Deenick EK, Niemela JE, Avery DT, Schickel JN, Tran DQ, Stoddard J, Zhang Y *et al* (2014) Immune dysregulation in human subjects with heterozygous germline mutations in CTLA4. *Science* 345: 1623–1627

Larkin J, Chiarion-Sileni V, Gonzalez R, Grob JJ, Rutkowski P, Lao CD, Cowey CL, Schadendorf D, Wagstaff J, Dummer R *et al* (2019) Five-year survival with combined nivolumab and ipilimumab in advanced melanoma. *N Engl J Med* 381: 1535–1546

Lin DY, Tanaka Y, Iwasaki M, Gittis AG, Su HP, Mikami B, Okazaki T, Honjo T, Minato N, Garboczi DN (2008) The PD-1/PD-L1 complex resembles the antigen-binding Fv domains of antibodies and T cell receptors. *Proc Natl Acad Sci USA* 105: 3011–3016

Lo B, Zhang K, Lu W, Zheng L, Zhang Q, Kanellopoulou C, Zhang Y, Liu Z, Fritz JM, Marsh R *et al* (2015) Patients with LRBA deficiency show CTLA4 loss and immune dysregulation responsive to abatacept therapy. *Science* 349: 436–440

Mezzadra R, Sun C, Jae LT, Gomez-Eerland R, de Vries E, Wu W, Logtenberg MEW, Slagter M, Rozeman EA, Hofland I *et al* (2017) Identification of CMTM6 and CMTM4 as PD-L1 protein regulators. *Nature* 549: 106–110

Mizuno R, Sugiura D, Shimizu K, Maruhashi T, Watada M, Okazaki IM, Okazaki T (2019) PD-1 primarily targets TCR signal in the inhibition of functional T cell activation. *Front Immunol* 10: 630

Ni X, Song Q, Cassady K, Deng R, Jin H, Zhang M, Dong H, Forman S, Martin PJ, Chen YZ *et al* (2017) PD-L1 interacts with CD80 to regulate graft-versus-leukemia activity of donor $CD8^+$ T cells. *J Clin Invest* 127: 1960–1977

Nishimura H, Nose M, Hiai H, Minato N, Honjo T (1999) Development of lupus-like autoimmune diseases by disruption of the PD-1 gene encoding an ITIM motif-carrying immunoreceptor. *Immunity* 11: 141–151

Paterson AM, Brown KE, Keir ME, Vanguri VK, Riella LV, Chandraker A, Sayegh MH, Blazar BR, Freeman GJ, Sharpe AH (2011) The programmed death-1 ligand 1:B7-1 pathway restrains diabetogenic effector T cells *in vivo*. *J Immunol* 187: 1097–1105

Qureshi OS, Zheng Y, Nakamura K, Attridge K, Manzotti C, Schmidt EM, Baker J, Jeffery LE, Kaur S, Briggs Z *et al* (2011) Trans-endocytosis of CD80 and CD86: a molecular basis for the cell-extrinsic function of CTLA-4. *Science* 332: 600–603

Qureshi OS, Kaur S, Hou TZ, Jeffery LE, Poulter NS, Briggs Z, Kenefeck R, Willox AK, Royle SJ, Rappoport JZ *et al* (2012) Constitutive clathrin-mediated endocytosis of CTLA-4 persists during T cell activation. *J Biol Chem* 287: 9429–9440

Ribas A, Wolchok JD (2018) Cancer immunotherapy using checkpoint blockade. *Science* 359: 1350–1355

Schildberg FA, Klein SR, Freeman GJ, Sharpe AH (2016) Coinhibitory pathways in the B7-CD28 ligand-receptor family. *Immunity* 44: 955–972

Schubert D, Bode C, Kenefeck R, Hou TZ, Wing JB, Kennedy A, Bulashevska A, Petersen BS, Schaffer AA, Gruning BA *et al* (2014) Autosomal dominant immune dysregulation syndrome in humans with CTLA4 mutations. *Nat Med* 20: 1410–1416

Schwab C, Gabrysch A, Olbrich P, Patino V, Warnatz K, Wolff D, Hoshino A, Kobayashi M, Imai K, Takagi M *et al* (2018) Phenotype, penetrance, and treatment of 133 cytotoxic T-lymphocyte antigen 4-insufficient subjects. *J Allergy Clin Immunol* 142: 1932–1946

Sharma P, Allison JP (2015) Immune checkpoint targeting in cancer therapy: toward combination strategies with curative potential. *Cell* 161: 205–214

Stamper CC, Zhang Y, Tobin JF, Erbe DV, Ikemizu S, Davis SJ, Stahl ML, Seehra J, Somers WS, Mosyak L (2001) Crystal structure of the B7-1/CTLA-4 complex that inhibits human immune responses. *Nature* 410: 608–611

Sugiura D, Maruhashi T, Okazaki IM, Shimizu K, Maeda TK, Takemoto T, Okazaki T (2019) Restriction of PD-1 function by cis-PD-L1/CD80 interactions is required for optimal T cell responses. *Science* 364: 558–566

Tekguc M, Wing JB, Osaki M, Long J, Sakaguchi S (2021) Treg-expressed CTLA-4 depletes CD80/CD86 by trogocytosis, releasing free PD-L1 on antigen-presenting cells. *Proc Natl Acad Sci USA* 118: e2023739118

Tivol EA, Borriello F, Schweitzer AN, Lynch WP, Bluestone JA, Sharpe AH (1995) Loss of CTLA-4 leads to massive lymphoproliferation and fatal multiorgan tissue destruction, revealing a critical negative regulatory role of CTLA-4. *Immunity* 3: 541–547

Walker LS, Sansom DM (2011) The emerging role of CTLA4 as a cell-extrinsic regulator of T cell responses. *Nat Rev Immunol* 11: 852–863

Walker LS, Sansom DM (2015) Confusing signals: recent progress in CTLA-4 biology. *Trends Immunol* 36: 63–70

Wei SC, Levine JH, Cogdill AP, Zhao Y, Anang NAS, Andrews MC, Sharma P, Wang J, Wargo JA, Pe'er D *et al* (2017) Distinct cellular mechanisms underlie anti-CTLA-4 and anti-PD-1 checkpoint blockade. *Cell* 170: e1117

Wei SC, Sharma R, Anang NAS, Levine JH, Zhao Y, Mancuso JJ, Setty M, Sharma P, Wang J, Pe'er D *et al* (2019) Negative Co-stimulation constrains T cell differentiation by imposing boundaries on possible cell states. *Immunity* 50: e1010

Wherry EJ, Kurachi M (2015) Molecular and cellular insights into T cell exhaustion. *Nat Rev Immunol* 15: 486–499

Wing K, Onishi Y, Prieto-Martin P, Yamaguchi T, Miyara M, Fehervari Z, Nomura T, Sakaguchi S (2008) CTLA-4 control over $Foxp3^+$ regulatory T cell function. *Science* 322: 271–275

Zhao Y, Lee CK, Lin CH, Gassen RB, Xu X, Huang Z, Xiao C, Bonorino C, Lu LF, Bui JD *et al* (2019) PD-L1:CD80 cis-heterodimer triggers the Co-stimulatory receptor CD28 while repressing the inhibitory PD-1 and CTLA-4 pathways. *Immunity* 51: e1059

License: This is an open access article under the terms of the Creative Commons Attribution License, which permits use, distribution and reproduction in any medium, provided the original work is properly cited.

Expanded View Figures

Figure EV1. Impact of co-expression of CD80, CD86 and PD-L1 on detection.

A CHO cells expressing PD-L1 alone or in combination with CD80 or CD86 were stained using the reagents shown and quantified by flow cytometry. The impact of CD80 and CD86 on detection of PD-L1 using PD-1 Ig (Left panel) or CTLA-4 Ig (abatacept- Right panel) is shown. Note the low binding of CTLA-4-Ig to CD86 is due to natural affinity differences (mean ± SD, three independent experiments). $^{*}P \leq 0.05$, $^{**}P \leq 0.01$, $^{****}P \leq 0.0001$, ns, not significant: RM one-way ANOVA.

B Concatenated flow cytometry plots (left panel) for Durvalumab-APC binding to DG-75 cells expressing PD-L1 or PD-L1/CD80, with dose response from three independent experiments showing mean ± SEM (middle panel). EC50 values of Durvalumab binding are plotted (right hand panel). $^{**}P \leq 0.01$: paired *t*-test.

C Down regulation of Jurkat CD28 following engagement by CD80 or CD86 in the presence or absence of PD-L1 is shown as a measure of CD28 interaction with ligands. CD28 MFI was determined using flow cytometry is quantified in right hand panel (mean ± SEM, three independent experiments, $^{****}P \leq 0.0001$: two-way ANOVA with Tukey's multiple comparisons test).

© 2023 The Authors

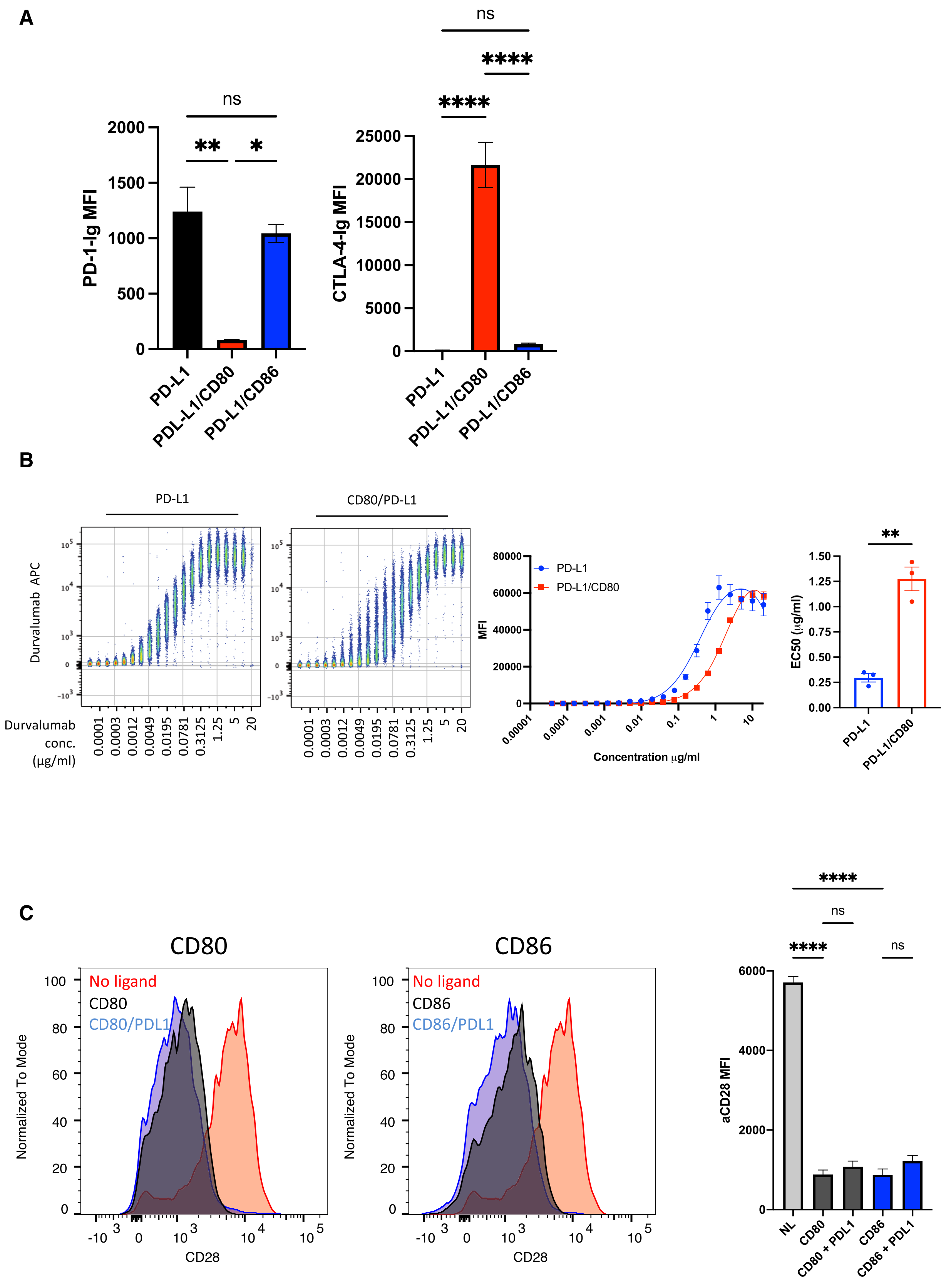

Figure EV1.

 © 2023 The Authors

Figure EV2. Measurement of transendocytosis by flow cytometry.

A Cartoon showing the principle of transendocytosis assays by flow cytometry (licenced under CC-BY from ref.22). Ligand (donor) cells expressing CD80 or CD86 proteins with GFP fusion tags (green plasma membrane) are labelled with CellTrace Violet (CTV^+, purple) and mixed with CTLA-4 expressing recipient cells (orange dots and membrane, CTV^-). During transendocytosis, plasma membrane expressed ligands are removed from donor cells (reduced green plasma membrane signal) and fluorescent ligand is now detected in CTLA-4 expressing recipient cells. Internalised ligands either separate from CTLA-4 (green dots) or remain colocalised (orange dots with green outline). Right hand panel—FACS plot shows representative result of TE, where ligand removal results in loss of GFP fluorescence from the CTV^+ donor cell, and a concomitant increase in GFP fluorescence in the CTV^- recipient cells, as indicated. Note that ligand gain by recipients is subject to continuous degradation and is not a reliable indicator of total ligand transfer. CD86 transendocytosis is unaffected by PD-L1.

B, C Transendocytosis assays were carried out using CHO cells at a ratio of 2 donor (CD86GFP alone or CD86GFP/PD-L1mCherry): 1 CTLA-4^+ recipient. Transendocytosis assays were performed for the times indicated. Representative FACS plots show CD86GFP downregulation (B) and PD-L1mCherry expression (C) at time points indicated. CD80 and CD80-PD-L1 cell lines have matched expression of ligands.

D Histograms showing CD80GFP and PD-L1mCherry expression levels on transduced DG-75 cell lines. Expression was monitored by measuring GFP (CD80) and mCherry (PD-L1) by flow cytometry.

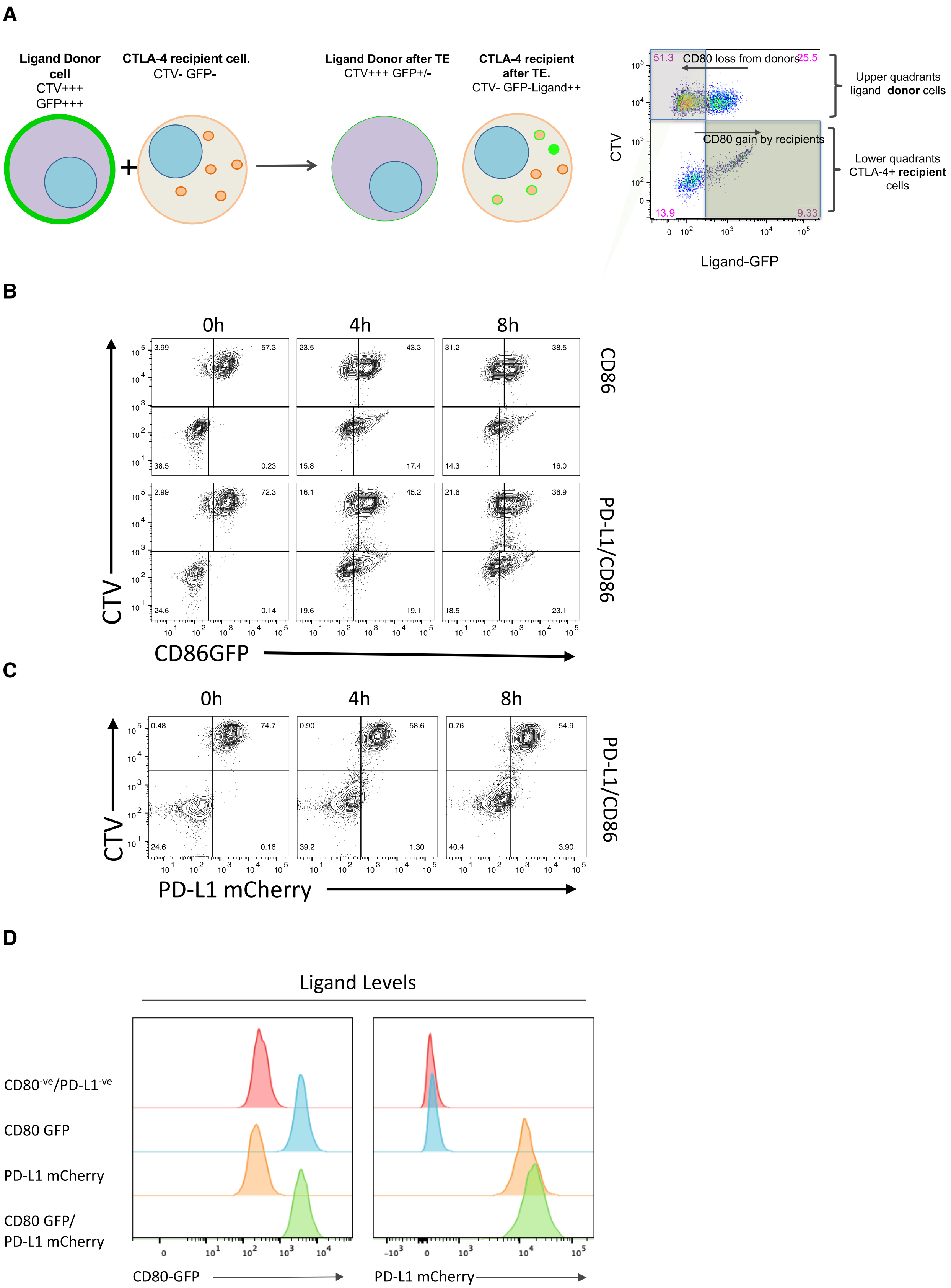

Figure EV2.

© 2023 The Authors

Figure EV3. Efficient removal of CD80 by transendocytosis liberates free PD-L1 for antibody detection.

A, B Anti-PD-L1 Ab (clone 29E.2A3, at 0.5 μg/ml) was used to detect PD-L1 in DG-75 cells co-expressing CD80GFP (A) or CD86GFP (B). Staining was carried out following transendocytosis with Jurkat cells expressing no CTLA-4, CTLA-4 WT or CTLA-4 Del36 (at a ratio of 1:1) for the indicated durations. (A) shows representative FACS plots of CD80GFP vs. PD-1 Ig at the time points indicated with full kinetic data plotted below (mean ± SEM, three independent experiments, ***$P \le 0.001$, ****$P \le 0.0001$: two-way ANOVA with Tukey's multiple comparisons test). (B) As for (A) except using CD86-PD-L1 expressing cells.

C Bar chart comparing the proportion of anti-PD-L1 positive cells after 24 h transendocytosis in DG-75 cells co-expressing PD-L1mCherry and CD80GFP or CD86GFP (mean ± SEM, three independent experiments, ****$P \le 0.0001$, ns not significant: two-way ANOVA with Tukey's multiple comparisons test).

© 2023 The Authors

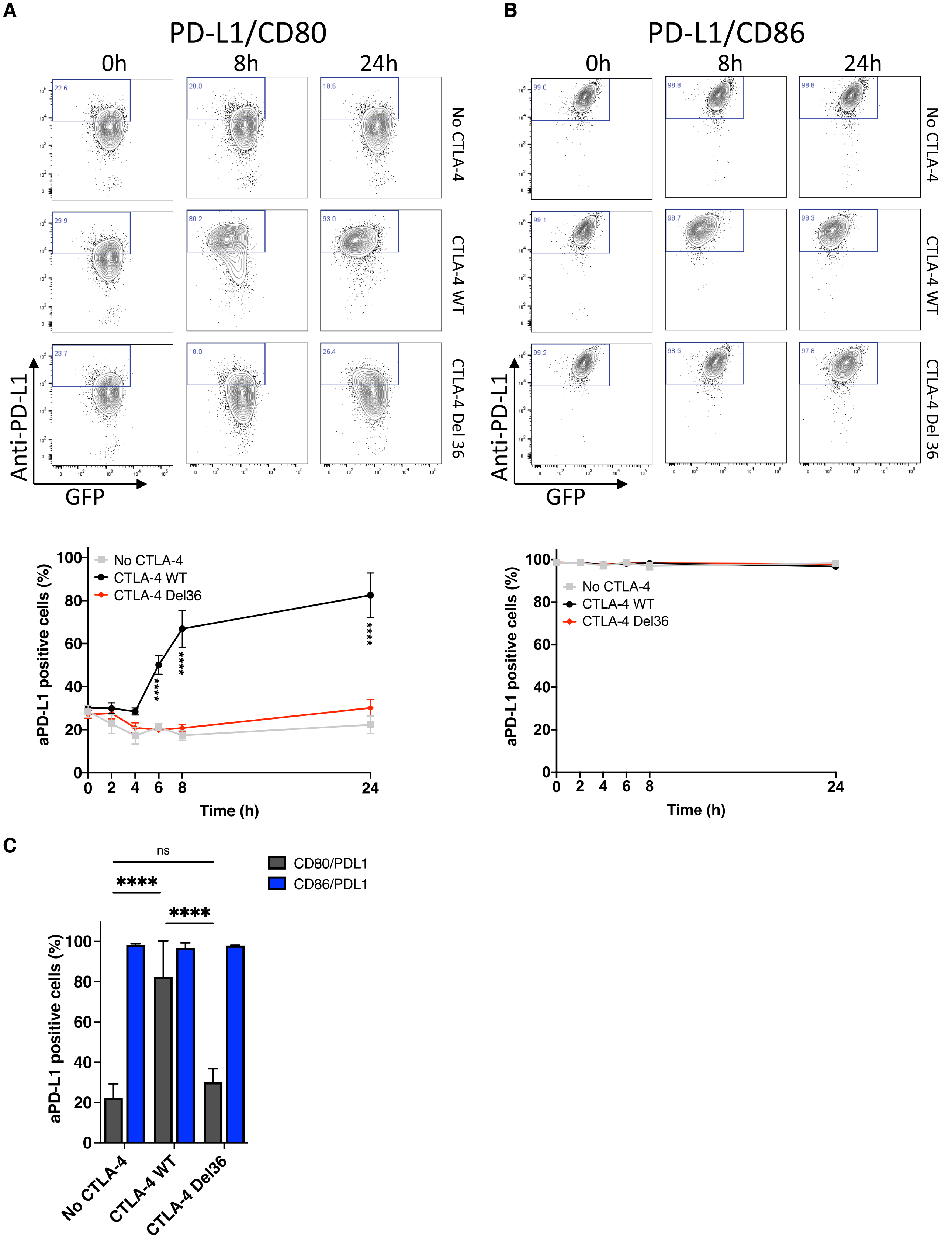

Figure EV3.

 © 2023 The Authors

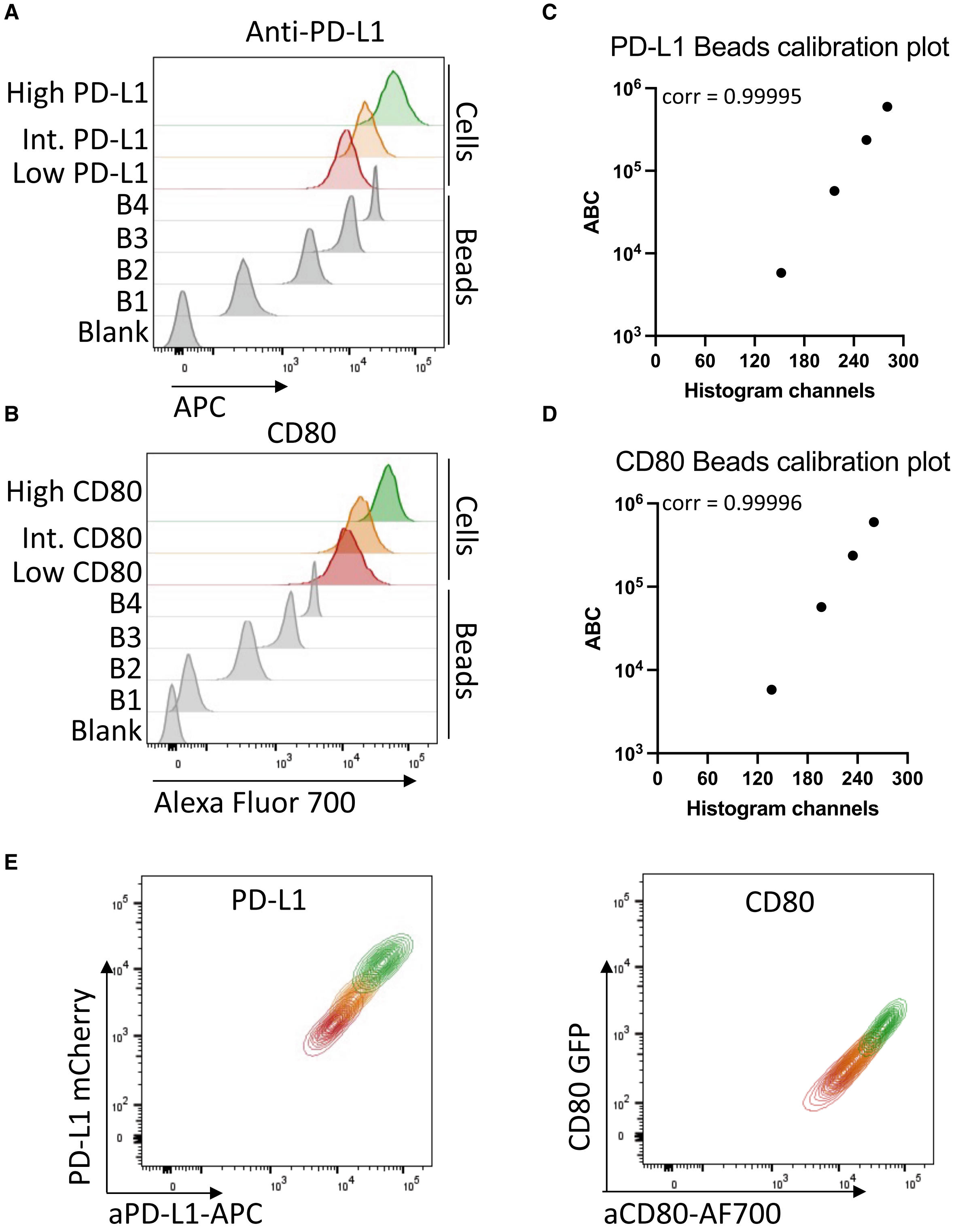
A
Anti-PD-L1
High PD-L1
Int. PD-L1
Low PD-L1
B4
B3
B2
B1
Blank
Cells
Beads
APC
B
CD80
High CD80
Int. CD80
Low CD80
Alexa Fluor 700
C
PD-L1 Beads calibration plot
corr = 0.99995
ABC
Histogram channels
D
CD80 Beads calibration plot
corr = 0.99996
E
PD-L1
PD-L1 mCherry
aPD-L1-APC
CD80
CD80 GFP
aCD80-AF700

Figure EV4.

Figure EV4. Calculation of CD80 and PD-L1 ratios on DG-75 cells.

A, B Representative histograms showing antibody staining at saturating concentrations for (A) PD-L1 (clone 29E.2A3, at 25 μg/ml) or (B) CD80 (clone L307.4, at 5 μg/ml) on Quantum™ Simply Cellular® bead populations (grey) or DG-75 cell lines expressing low (red), intermediate (orange) or high (green) levels of respective ligand.

C, D Calibration curves were fitted based on the bead MFI and the corresponding antibody binding capacity (ABC). Representative calibration curves are shown for PD-L1 (C) and CD80 (D) staining with correlation coefficient calculated after curve fitting in the manufacturer-provided QuickCal® spreadsheet.

E Correlation between anti-PD-L1 staining and mCherry intensity, or anti-CD80 staining and GFP intensity in representative FACS plots showing the cell lines from (A) and (B).

Figure EV5. Abatacept immunoprecipitates PD-L1:CD80 interactions.

A Western blot analysis of Abatacept immunoprecipitates from indicated DG-75 cell lines, with and without the BS_3 crosslinker. Precipitates were immunoblotted for GFP (CD80/ CD86) and mCherry (PD-L1) as indicated. Belatacept modulates anti-PD-L1 antibody binding but fails to confer PD-1 Ig detection.

B Concatenated plot of a one in four serial dilution of Belatacept (starting at 20 μg/ml) of CD80 only (top left hand panel) or CD80/PD-L1 co-expressing DG-75 (top right hand panel), with combined titration curves (bottom left hand panel) and EC50 values (bottom right hand values) of Belatacept binding of CD80 only or PD-L1/CD80 co-expressing DG-75. Data are representative of three independent experiments showing mean ± SEM. ns, not significant: paired *t*-test.

C Concatenated plot of a one in four serial dilution of Belatacept (starting at 20 μg/ml) followed by PD-L1 detection using antibody (MIH3 or 29E.2A3 clone) or PD-1 Ig, all at 1ug/ml.

D Graphical representation showing PD-L1 detection under conditions used in (C). Data are representative of three independent experiments showing mean ± SEM.

 © 2023 The Authors

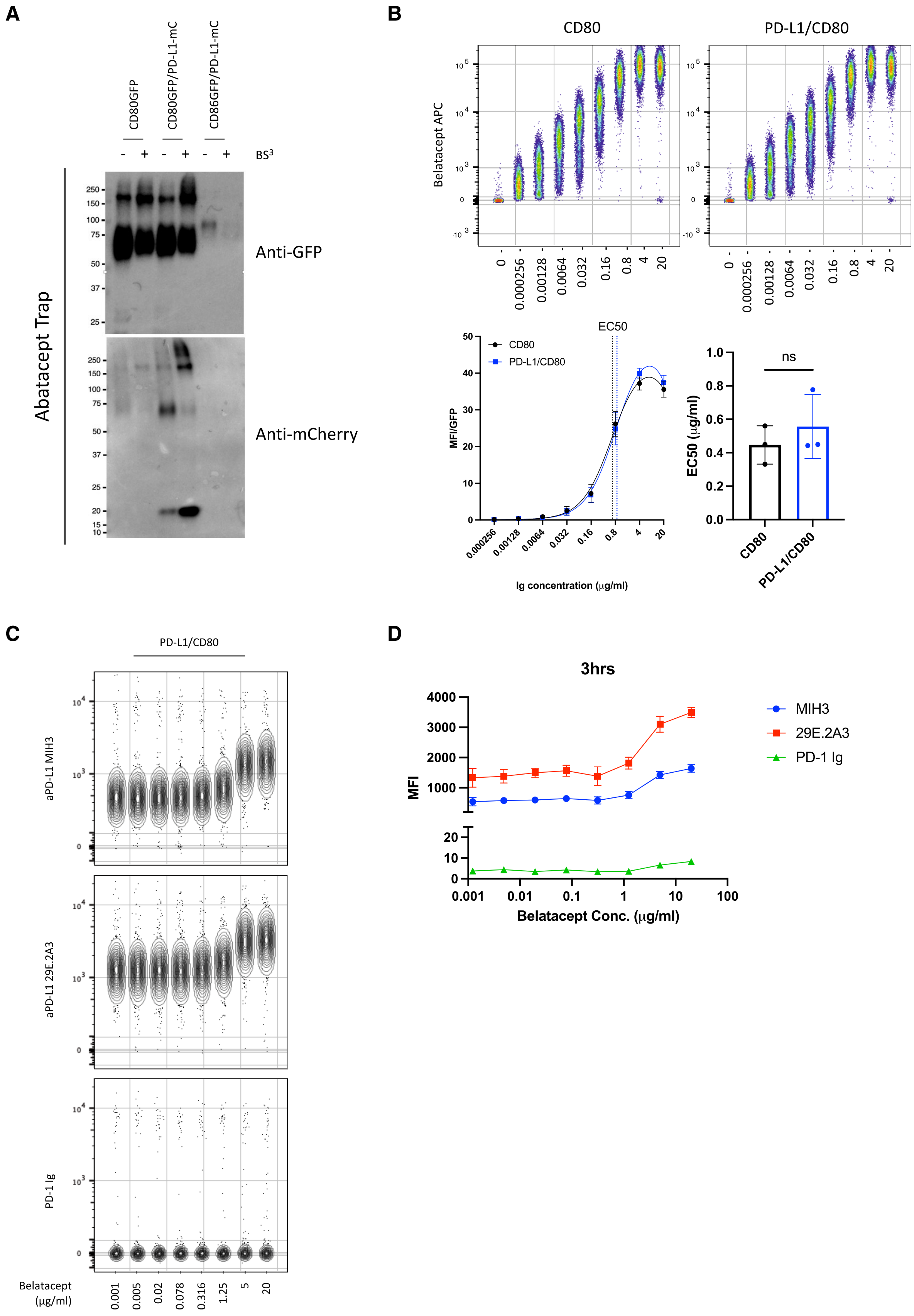
A
CD80GFP
CD80GFP/PD-L1-mC
CD86GFP/PD-L1-mC
BS3
Abatacept Trap
Anti-GFP
Anti-mCherry
B
CD80
PD-L1/CD80
Belatacept APC
EC50
CD80
PD-L1/CD80
MFI/GFP
Ig concentration (µg/ml)
ns
EC50 (µg/ml)
C
PD-L1/CD80
aPD-L1 MIH3
aPD-L1 29E.2A3
PD-1 Ig
Belatacept (µg/ml)
D
3hrs
MIH3
29E.2A3
PD-1 Ig
MFI
Belatacept Conc. (µg/ml)

Figure EV5.
