# Supplementary material for: The CTLA‐4 immune checkpoint protein regulates PD‐L1:PD‐1 interaction via transendocytosis of its ligand CD80
Source: EMBO J. 2023 Feb 2;42(5):e111556. doi: 10.15252/embj.2022111556 (PMC9975936; doi:10.15252/embj.2022111556)
Supplement: Supplementary file 3 [file EMBJ-42-e111556-s002.pdf]

THE EMBO JOURNAL

The CTLA-4 immune checkpoint protein regulates PD-L1:PD-1 interaction via transendocytosis of its ligand CD80

Alan Kennedy, Maximillian Robinson, Claudia Hinze, Erin Waters, Cayman Williams, Neil Halliday, Simon Dovedi, and David Sansom
DOI: 10.15252/embj.2022111556

Corresponding author(s): David Sansom (d.sansom@ucl.ac.uk)

Review Timeline:

Submission Date:	29th Apr 22
Editorial correspondence:	14th Jun 22
Editorial Decision:	21st Jun 22
Revision Received:	31st Oct 22
Editorial Decision:	28th Nov 22
Revision Received:	16th Dec 22
Accepted:	21st Dec 22

Editor: Karin Dumstrei

Transaction Report:

(Note: With the exception of the correction of typographical or spelling errors that could be a source of ambiguity, letters and reports are not edited. Depending on transfer agreements, referee reports obtained elsewhere may or may not be included in this compilation. Referee reports are anonymous unless the Referee chooses to sign their reports.)

Dear David,

Thank you for submitting your manuscript to The EMBO Journal. Your study has now been seen by three referees and their comments are provided below.

The referees find the analysis interesting but also find that further insight is needed in order to consider publication here. Both referees #1 and 3 find that we need some functional insight in addition to using primary cells.

As it is not clear how much data you have on hand or what you can generate within a reasonable timeframe I find it most helpful to do a pre-consultation before taking a decision on the manuscript.

Could you send me a point-by-point response to the concerns raised and based upon this I will then take the decision on the paper.

We can also schedule a zoom call to discuss the points further.

With best wishes

Karin

Karin Dumstrei, PhD
Senior Editor
The EMBO Journal

Referee #1

PD1 and CTLA4 are important immune checkpoints that are targeted in cancer therapy. PDL1 and CD80, which are ligands for PD1 and CTLA4 respectively interact with each other. This interaction mainly occurs in cis. Previous work by this group has uncovered a process termed transendocytosis where CTLA4 acquires CD80 and CD86 from adjacent cells. In this study. Kennedy et al have investigated how PDL1-CD80 interaction impact the binding of these ligands to their receptors. In line with some of the earlier studies they observed that upon engagement of PDL1 by CD80 in trans binding of PDL1 to PD-1 is blocked whereas CD80 still can bind CTLA4. In addition, the authors show that CTLA4 mediated transendocytosis of CD80 does not result in depletion of PDL1 bound to CD80. Thus CD80-transendocytosis liberates PD-L1 for engagement of PD-1 in a time-dependent manner. Earlier work showed that the cytoplasmic tail of CTLA-4 is required for transendocytosis. In this study the authors provide evidence that simple binding of CTLA4 is insufficient to liberate PDL1 and that trogocytosis which also results in the removal of CD80 and which does not require the cytoplasmic tail of CTLA4 is insufficient to liberate PDL1. This is a well-conducted study and the manuscript is well written and the data are clearly presented. However, the study is limited to binding studies and experiments that monitor the expression levels of CD80 and PDL1 on cell lines in presence of cell lines expressing CTLA4. No functional studies have been performed to address the impact of CTLA4 on reinstalling PDL1 mediated inhibition. Consequently, they have not addressed whether full length CTLA4 is really significantly more efficient in reinstalling PDL1-mediated PD1-inhibition than CTLA4 lacking its cytoplasmic tail. This is an important question in this context since CTLA4 lacking its cytoplasmic tail (termed CTLA4 Del36 in this study) is expressed at much higher levels and might thus strongly engage CD80 (and thereby enhance PD1-inhibition via PDL1) despite the fact that it is less efficient in reducing CD80 on adjacent cells than full length CTLA4. Due to this

lack functional data the gives limited insight into how the interaction of PDL1 and CD80 in cis and the interaction of CD80 and CTLA4 in trans impact on the PD1 and CTLA4/CD28 pathways.

Major concerns:

1. Important aspects shown in this manuscript have been reported earlier (e.g. by Sugiura et al). This is a rather small study with a limited set of experiments in cell lines. More experiments - especially functional experiments should have been performed to gain a more comprehensive insight on the role of CTLA-4 mediated transendocytosis of CD80 in PD-1 function to justify publication in a renowned high-impact journal with a broad readership.

2. As pointed out above the major limitation of the study is that it lacks functional data. It would be important to learn how CTLA4 activity impacts on PD-1 inhibition on T cells or T cell lines (in the presence or absence of CD28 costimulation). Without functional experiments it cannot be claimed that transendocytosis of CD80 is required to effectively control PD-L1-PD-1 interactions.

3. For the experiments summarized in figure 1 there is no information provided how often the experiments were repeated and statistical analysis is lacking

4. No experiments with primary APC have been performed. PDL1-CD80 coexpression mainly occurs on professional APC which frequently also express the second ligand for CTLA4 and in many cases also PD-L2 the second ligand for PD-1. Potentially blockade of PDL1 by CD80 engagement could have less impact if PDL2 is present.

Minor points:

In the binding of therapeutic PDL1 antibodies which bind very strongly to PDL1 also blocked by CD80 binding in cis?

Figure 7: the authors report that abatacept does not alter PD-1 binding; it can be expected that this is also the case for belatacept but if would be interesting to address this.

Ligand levels: The authors should show fluorophore expressing and ligand expression (e.g. GFP expression and CD80 staining in Fig. EV4)
If CD80 and CD86 are coexpressed is CD80 preferentially removed from the APC via CTLA-4 mediated transendocytosis?

Does CD80 has to be expressed in excess of PD-L1 to prevent PD-1-Ig binding or is this observed when both ligands are expressed at equimolar concentrations?

Referee #2

Kennedy et al. show in in vitro experiments 1) CTLA4 on donor cells specially bind to CD80 in the heterodimer of PD-L1:CD80; 2) CTLA4 transendocytosis selectively remove CD80 but not PD-L1; 3) Effective removal of CD80 via transendocytosis requires CTLA4 cytoplasmic domain; 4) Transendocytosis of CD80 by CTLA4 but not blocking soluble CTLA4 Ig effectively liberates PD-L1. The authors conclude that efficient CD80 depletion by transendocytosis but not simply CTLA-4 binding to CD80 is required to effectively liberate PD-L1 that can engage PD-1. These observations

are interesting, and the manuscript is clearly and concisely written.

There is a minor concern need to be addressed. Most of the recent publications on cis PD-L1/CD80 interactions are based on in vitro transfection experiments with PD-L1 and CD80. Those reports and the current report all elegantly show that there is only cis PD-L1/CD80 interactions. However, previous experiments (Park et al: Blood 2010; Yi et al J. Immunol. 2011, Deng et al 2015; Ni et al: JCI 2017) clearly indicate the existence of trans PD-L1/CD80 interactions in vivo. In vitro interactions cannot be used to exclude complex in vivo interactions. This notion should be made in the discussion.

Referee #3

Sansom review

Adding to several recent publications on the cis CD80/PD-L1 interaction, the authors show that transendocytosis by CTLA4 of a CD80/PD-L1 heterodimer removes CD80 but not PD-L1 from the cell surface, allowing the PD-L1 to interact with PD-1. They propose that CD80 can operate as a switch and by preventing PD-L1 from interacting with PD-1 and by its higher avidity binding to CTLA4 also protect CD86 from transendocytosis by CTLA4, the CD80 can promote enhanced T cell responses by inhibiting both CTLA-4 and PD-1 pathways. The work is well done and deepens our understanding of a wicked complicated set of interactions but is with transfectants and not natural cells. No direct experiments on immune function are performed.

Major:
1. Zhao et al propose that the CD80/PD-L1 interaction, by reducing CD80 to a monomeric state, reduces the avidity for CTLA-4. Do a dose titration of CTLA4-Ig on CHO-CD80 and CHO-CD80/PD-L1 cells and determine the EC50 of CTLA4 binding and see if the avidity is altered. This will have to be done on CHO cell transfectants since endogenous CD80 and CD86 would complicate the analysis in DG-75 cells.
2. Specify concentrations on x-axis of Figure 7 (these are DG-75 cells). Calculate EC50 of CTLA4-Ig binding to CD80 vs CD80/PD-L1 from Fig 7A.
3. What is the molar ratio of PD-L1 to CD80 in the transfectants? Which is in excess? This cannot be determined by FACS but could be done by mass spectroscopy.
4. How much CD80, CD86, PD-L1 does the Burkitts lymphoma cell line DG-75 express (generally Burkitts lines express)? Show in a supplemental figure. Is there binding to GFP-less native CD80 or CD86 which may complicate interpretation?

Minor:
5. In introduction, distinguish characteristics of transendocytosis and trogocytosis. How are they distinguished?
6. "acquisition by the CTLA-4+ recipient population using a well-established assay " describe cells used in assay, particularly CTLA-4 recipient cells.
7. Move Figure EV2 to main text so assay is clear to reader at beginning.
8. The labeling of the figures and descriptions in the figure legends is in many places inadequate to clearly understand what has been done and how. There are many examples but to specify a few:
9. In 1D, specify ab used for IP and for WB
10. The labeling of Figure 2 does not provide enough information. The donor and recipient cells for each panel (and their CTV labeling) should be clearly indicated in the figure.

11. In 2A, are the upper left and right panels duplicate assays?
12. Throughout all figures, where "ligand" is written, specify molecule. Do not use "no ligand"
13. Methods says "ratios of donor: recipient cells and incubation times indicated in the figure legends." But figures often do not provide these numbers. Recipient cells need to be specified in figures.
14. In 3C and similar, the left panel y-axis is labeled "ligand remaining". Specify which ligand in each case; presumably CD80?
15. In methods, give equation of how ligand remaining was calculated in Figure 2D and similar figures.
16. In 2A, choice of overnight incubation is a poor one since the amount of detectable transferred ligand is low (presumably because of degradation). Suggest using a time point where degradation is less dominant.
17. In 2B, the question is whether the PD-L1mCherry is being transendocytosed by the CTLA4 cell. Say the red quadrant is loss of PD-L1. There does not seem to be a shift in the CTV positive population but the MFI intensity of the total cell population of the lower (CTV negative) population is clearly shifted to the right. Why does this shift not mean that PD-L1 is being broadly taken up by the recipient cells, maybe on the cell surface and not internalized?
18. Calculate statistical significance between lines in 2D (CD80 vs CD80+PD-L1)
19. "CTLA-4 cytoplasmic domain was required for effective time-dependent depletion of CD80 and CD86 "
Soften this statement (required is too extreme). In general, this data is discussed appropriately but the titles are extreme. There is some trogocytosis/transendocytosis by Cytdel (11% vs control of 2%)
20. "The reason for the differences between these observations currently remain unclear but may relate to differences in how transendocytosis was measured. "
Describe differences between your method and Zhao's.
21. In the discussion, also note that in natural cells, PD-L1 is associated with CMTM6 and this is not replicated in transfectants.
22. Specify GFP used in methods and linker length.
23. Specify CTLA-4 del36 in methods.

1st Editorial Decision 21st Jun 2022

Dear David,

Thank you for sending me the point-by-point response. I have now had a chance to look at it and I appreciate the proposed experiments.

I would therefore like to invite you to submit a revised version addressing the concerns as proposed.

When preparing your letter of response to the referees' comments, please bear in mind that this will form part of the Review Process File, and will therefore be available online to the community. For more details on our Transparent Editorial Process, please visit our website: https://www.embopress.org/page/journal/14602075/authorguide#transparentprocess

I thank you for the opportunity to consider your work for publication. I look forward to your revision.

with best wishes

Karin

Karin Dumstrei, PhD
Senior Editor
The EMBO Journal

Instructions for preparing your revised manuscript:

I have attached a PDF with helpful tips on how to prepare the revised version.

Guide For Authors: https://www.embopress.org/page/journal/14602075/authorguide

We realize that it is difficult to revise to a specific deadline. In the interest of protecting the conceptual advance provided by the work, we recommend a revision within 3 months (19th Sep 2022).

As a matter of policy, competing manuscripts published during this period will not negatively impact on our assessment of the conceptual advance presented by your study. However, we request that you contact the editor as soon as possible upon publication of any related work, to discuss how to proceed.

If you require more time to complete the revisions let me know as as I can grant an extension.

Use the link below to submit your revision:

https://emboj.msubmit.net/cgi-bin/main.plex

Referee #1:

PD1 and CTLA4 are important immune checkpoints that are targeted in cancer therapy. PDL1 and CD80, which are ligands for PD1 and CTLA4 respectively interact with each other. This interaction mainly occurs in cis. Previous work by this group has uncovered a process termed transendocytosis where CTLA4 acquires CD80 and CD86 from adjacent cells. In this study. Kennedy et al have investigated how PDL1-CD80 interaction impact the binding of these ligands to their receptors. In line with some of the earlier studies they observed that upon engagement of PDL1 by CD80 in trans binding of PDL1 to PD-1 is blocked whereas CD80 still can bind CTLA4. In addition, the authors show that CTLA4 mediated transendocytosis of CD80 does not result in depletion of PDL1 bound to CD80. Thus CD80-transendocytosis liberates PD-L1 for engagement of PD-1 in a time-dependent manner. Earlier work showed that the cytoplasmic tail of CTLA-4 is required for transendocytosis. In this study the authors provide evidence that simple binding of CTLA4 is insufficient to liberate PDL1 and that trogocytosis which also results in the removal of CD80 and which does not require the cytoplasmic tail of CTLA4 is insufficient to liberate PDL1. This is a well-conducted study and the manuscript is well written and the data are clearly presented. However, the study is limited to binding studies and experiments that monitor the expression levels of CD80 and PDL1 on cell lines in presence of cell lines expressing CTLA4. No functional studies have been performed to address the impact of CTLA4 on reinstalling PDL1 mediated inhibition. Consequently, they have not addressed whether full length CTLA4 is really significantly more efficient in reinstalling PDL1-

mediated PD1-inhibition than CTLA4 lacking its cytoplasmic tail. This is an important question in this context since CTLA4 lacking its cytoplasmic tail (termed CTLA4 Del36 in this study) is expressed at much higher levels and might thus strongly engage CD80 (and thereby enhance PD1-inhibition via PDL1) despite the fact that it is less efficient in reducing CD80 on adjacent cells than full length CTLA4. Due to this lack functional data the gives limited insight into how the interaction of PDL1 and CD80 in cis and the interaction of CD80 and CTLA4 in trans impact on the PD1 and CTLA4/CD28 pathways.
Major concerns:
1. Important aspects shown in this manuscript have been reported earlier (e.g. by Sugiura et al). This is a rather small study with a limited set of experiments in cell lines. More experiments - especially functional experiments should have been performed to gain a more comprehensive insight on the role of CTLA-4 mediated transendocytosis of CD80 in PD-1 function to justify publication in a renowned high-impact journal with a broad readership.
2. As pointed out above the major limitation of the study is that it lacks functional data. It would be important to learn how CTLA4 activity impacts on PD-1 inhibition on T cells or T cell lines (in the presence or absence of CD28 costimulation). Without functional experiments it cannot be claimed that transendocytosis of CD80 is required to effectively control PD-L1-PD-1 interactions.
3. For the experiments summarized in figure 1 there is no information provided how often the experiments were repeated and statistical analysis is lacking
4. No experiments with primary APC have been performed. PDL1-CD80 coexpression mainly occurs on professional APC which frequently also express the second ligand for CTLA4 and in many cases also PD-L2 the second ligand for PD-1. Potentially blockade of PDL1 by CD80 engagement could have less impact if PDL2 is present.
Minor points:
In the binding of therapeutic PDL1 antibodies which bind very strongly to PDL1 also blocked by CD80 binding in cis?
Figure 7: the authors report that abatacept does not alter PD-1 binding; it can be expected that this is also the case for belatacept but if would be interesting to address this.
Ligand levels: The authors should show fluorophore expressing and ligand expression (e.g. GFP expression and CD80 staining in Fig. EV4)
If CD80 and CD86 are coexpressed is CD80 preferentially removed from the APC via CTLA-4 mediated transendocytosis?
Does CD80 has to be expressed in excess of PD-L1 to prevent PD-1-Ig binding or is this observed when both ligands are expressed at equimolar concentrations?

Referee #2:

Kennedy et al. show in in vitro experiments 1) CTLA4 on donor cells specially bind to CD80 in the heterodimer of PD-L1:CD80; 2) CTLA4 transendocytosis selectively remove CD80 but not PD-L1; 3) Effective removal of CD80 via transendocytosis requires CTLA4 cytoplasmic domain; 4) Transendocytosis of CD80 by CTLA4 but not blocking soluble CTLA4 Ig effectively liberates PD-L1. The authors conclude that efficient CD80 depletion by transendocytosis but not simply CTLA-4 binding to CD80 is required to effectively liberate PD-L1 that can engage PD-1. These observations are interesting, and the manuscript is clearly and concisely written.

There is a minor concern need to be addressed. Most of the recent publications on cis PD-L1/CD80 interactions are based on in vitro transfection experiments with PD-L1 and CD80. Those reports and the current report all elegantly show that there is only cis PD-L1/CD80 interactions. However, previous experiments (Park et al: Blood 2010; Yi et al J. Immunol. 2011, Deng et al 2015; Ni et al: JCI 2017) clearly indicate the existence of trans PD-L1/CD80 interactions in vivo. In vitro interactions cannot be used to exclude complex in vivo interactions. This notion should be made in the discussion.

Referee #3:

Sansom review

Adding to several recent publications on the cis CD80/PD-L1 interaction, the authors show that transendocytosis by CTLA4 of a CD80/PD-L1 heterodimer removes CD80 but not PD-L1 from the cell surface, allowing the PD-L1 to interact with PD-1. They propose that CD80 can operate as a switch and by preventing PD-L1 from interacting with PD-1 and by its higher avidity binding to CTLA4 also protect CD86 from transendocytosis by CTLA4, the CD80 can promote enhanced T cell responses by inhibiting both CTLA-4 and PD-1 pathways. The work is well done and deepens our understanding of a wicked complicated set of interactions but is with transfectants and not natural cells. No direct experiments on immune function are performed.

Major:
1. Zhao et al propose that the CD80/PD-L1 interaction, by reducing CD80 to a monomeric state, reduces the avidity for CTLA-4.

Do a dose titration of CTLA4-Ig on CHO-CD80 and CHO-CD80/PD-L1 cells and determine the EC50 of CTLA4 binding and see if the avidity is altered. This will have to be done on CHO cell transfectants since endogenous CD80 and CD86 would complicate the analysis in DG-75 cells.
2. Specify concentrations on x-axis of Figure 7 (these are DG-75 cells). Calculate EC50 of CTLA4-Ig binding to CD80 vs CD80/PD-L1 from Fig 7A.
3. What is the molar ratio of PD-L1 to CD80 in the transfectants? Which is in excess? This cannot be determined by FACS but could be done by mass spectroscopy.
4. How much CD80, CD86, PD-L1 does the Burkitts lymphoma cell line DG-75 express (generally Burkitts lines express)? Show in a supplemental figure. Is there binding to GFP-less native CD80 or CD86 which may complicate interpretation?

Minor:
5. In introduction, distinguish characteristics of transendocytosis and trogocytosis. How are they distinguished?
6. "acquisition by the CTLA-4+ recipient population using a well-established assay " describe cells used in assay, particularly CTLA-4 recipient cells.
7. Move Figure EV2 to main text so assay is clear to reader at beginning.
8. The labeling of the figures and descriptions in the figure legends is in many places inadequate to clearly understand what has been done and how. There are many examples but to specify a few:
9. In 1D, specify ab used for IP and for WB
10. The labeling of Figure 2 does not provide enough information. The donor and recipient cells for each panel (and their CTV labeling) should be clearly indicated in the figure.
11. In 2A, are the upper left and right panels duplicate assays?
12. Throughout all figures, where "ligand" is written, specify molecule. Do not use "no ligand"
13. Methods says "ratios of donor: recipient cells and incubation times indicated in the figure legends." But figures often do not provide these numbers. Recipient cells need to be specified in figures.
14. In 3C and similar, the left panel y-axis is labeled "ligand remaining". Specify which ligand in each case; presumably CD80?
15. In methods, give equation of how ligand remaining was calculated in Figure 2D and similar figures.
16. In 2A, choice of overnight incubation is a poor one since the amount of detectable transferred ligand is low (presumably because of degradation). Suggest using a time point where degradation is less dominant.
17. In 2B, the question is whether the PD-L1mCherry is being transendocytosed by the CTLA4 cell. Say the red quadrant is loss of PD-L1. There does not seem to be a shift in the CTV positive population but the MFI intensity of the total cell population of the lower (CTV negative) population is clearly shifted to the right. Why does this shift not mean that PD-L1 is being broadly taken up by the recipient cells, maybe on the cell surface and not internalized?
18. Calculate statistical significance between lines in 2D (CD80 vs CD80+PD-L1)
19. "CTLA-4 cytoplasmic domain was required for effective time-dependent depletion of CD80 and CD86 "
Soften this statement (required is too extreme). In general, this data is discussed appropriately but the titles are extreme. There is some trogocytosis/transendocytosis by Cytdel (11% vs control of 2%)
20. "The reason for the differences between these observations currently remain unclear but may relate to differences in how transendocytosis was measured. "
Describe differences between your method and Zhao's.
21. In the discussion, also note that in natural cells, PD-L1 is associated with CMTM6 and this is not replicated in transfectants.
22. Specify GFP used in methods and linker length.
23. Specify CTLA-4 del36 in methods.

Point by point response

Referee #1:

PD1 and CTLA4 are important immune checkpoints that are targeted in cancer therapy. PDL1 and CD80, which are ligands for PD1 and CTLA4 respectively interact with each other. This interaction mainly occurs in cis. Previous work by this group has uncovered a process termed transendocytosis where CTLA4 acquires CD80 and CD86 from adjacent cells. In this study. Kennedy et al have investigated how PDL1-CD80 interaction impact the binding of these ligands to their receptors. In line with some of the earlier studies they observed that upon engagement of PDL1 by CD80 in trans binding of PDL1 to PD-1 is blocked whereas CD80 still can bind CTLA4. In addition, the authors show that CTLA4 mediated transendocytosis of CD80 does not result in depletion of PDL1 bound to CD80. Thus CD80-transendocytosis liberates PD-L1 for engagement of PD-1 in a time-dependent manner. Earlier work showed that the cytoplasmic tail of CTLA-4 is required for transendocytosis. In this study the authors provide evidence that simple binding of CTLA4 is insufficient to liberate PDL1 and that trogocytosis which also results in the removal of CD80 and which does not require the cytoplasmic tail of CTLA4 is insufficient to liberate PDL1. This is a well-conducted study and the manuscript is well written and the data are clearly presented. However, the study is limited to binding studies and experiments that monitor the expression levels of CD80 and PDL1 on cell lines in presence of cell lines expressing CTLA4. No functional studies have been performed to address the impact of CTLA4 on reinstalling PDL1 mediated inhibition. Consequently, they have not addressed whether full length CTLA4 is really significantly more efficient in reinstalling PDL1-mediated PD1-inhibition than CTLA4 lacking its cytoplasmic tail. This is an important question in this context since CTLA4 lacking its cytoplasmic tail (termed CTLA4 Del36 in this study) is expressed at much higher levels and might thus strongly engage CD80 (and thereby enhance PD1-inhibition via PDL1) despite the fact that it is less efficient in reducing CD80 on adjacent cells than full length CTLA4. Due to this lack functional data the gives limited insight into how the interaction of PDL1 and CD80 in cis and the interaction of CD80 and CTLA4 in trans impact on the PD1 and CTLA4/CD28 pathways.

We thank the referee for their thoughtful comments and have addressed their point-by-point concerns below. The data in our manuscript shows clearly that CTLA-4 molecule is effective at removing CD80 (from CD80-PDL1 cells) by transendocytosis and restores PD-L1 availability. We make the comparison with CTLA-4 Del36 (trogocytosis) partly because we know this molecule is defective in transendocytosis and partly because the studies of Sakaguchi and colleagues (Tekguc et al., 2021) claim that a similar tailless CTLA-4 molecule in mice is effective in regulating PD-L1 expression. However, the Del36 molecule is problematic both because it is highly overexpressed at the plasma membrane and because it does not exist in nature. It is worth reflecting that there are no reported mutations of the highly conserved CTLA-4 cytoplasmic domain in humans, despite a number of mutations being detected in the ligand binding domain. This strongly suggests that such mutations are not tolerated and that an intact CTLA-4 cytoplasmic domain is therefore essential for proper CTLA-4 function.

Thus, while we make comparisons for reference here, it is only the WT CTLA-4 molecule that can be considered to have physiological behaviour. Therefore, the referee's point ".... [Del36] is expressed at much higher levels and might thus strongly engage CD80 (and thereby enhance PD1-inhibition via PDL1) despite the fact that it is less efficient......." is rather moot. Nonetheless, we go on to show this is not the case in our manuscript and that WT CTLA-4 is more efficient in spite of higher levels of surface expression by Del 36.

Major concerns:

1. Important aspects shown in this manuscript have been reported earlier (e.g. by Sugiura et al). This is a rather small study with a limited set of experiments in cell lines. More experiments - especially functional experiments should have been performed to gain a more comprehensive insight on the role of CTLA-4 mediated transendocytosis of CD80 in PD-1 function to justify publication in a renowned high-impact journal with a broad readership. *We agree our data support the work of Sugiura et al.,(Sugiura et al., 2019) and we cite their excellent work appropriately in the manuscript. However, our findings represent a significant extension of current understanding. Moreover, we have now provided a number of new experiments which we believe further enhance the importance of our manuscript.*

2. As pointed out above the major limitation of the study is that it lacks functional data. It would be important to learn how CTLA4 activity impacts on PD-1 inhibition on T cells or T cell lines (in the presence or absence of CD28 costimulation). Without functional experiments it cannot be claimed that transendocytosis of CD80 is required to effectively control PD-L1-PD-1 interactions. *We have now provided data in a new figure 7, which shows that removal of CD80 by transendocytosis (and not trogocytosis) liberates PD-L1 which is capable of binding to PD-1 on Jurkat T cells and inhibiting CD69 expression. This shows that PD-L1 is indeed functional following removal of CD80 by transendocytosis. We also performed these experiments in the absence of CD28 as suggested, which again show that liberated PD-L1 can bind PD-1 and inhibit T cell activation signals propagated via the TCR.*

Royal Free & University College Medical School Rowland Hill Street, London NW3 2PF
Tel: +44 (0)20 7794 0500 x 22469. Email: d.sansom@ucl.ac.uk
http://www.ucl.ac.uk/medicalschool/infection-immunity/

3. For the experiments summarized in figure 1 there is no information provided how often the experiments were repeated and statistical analysis is lacking. *We apologise for this oversight; we have added this information to figure 1C.*

4. No experiments with primary APC have been performed. PDL1-CD80 co-expression mainly occurs on professional APC which frequently also express the second ligand for CTLA4 and in many cases also PD-L2 the second ligand for PD-1. Potentially blockade of PDL1 by CD80 engagement could have less impact if PDL2 is present.
We acknowledge this important point; however it is not clear that all of these additional issues can all be adequately studied within the scope of the present manuscript. The impact of PD-L2 is largely unknown and the cell types and conditions where primary human APCs express CD80, PD-L1, PD-L2 and CD86 are not established. We note that, data from Sugiura et.al., suggest that even in the presence of detectable PD-L2, the major binding of PD-1, was via PD-L1. However, their data used splenic DC and thioglycollate elicited Macrophages to identify PD-L1 expressing cells with differing levels of CD80, neither of which are realistically available for human studies. At present, we believe the best option is to use the B cell lines we describe in the manuscript, which are clearly a relevant APC cell type from an immunological perspective.

Minor points:
In the binding of therapeutic PDL1 antibodies which bind very strongly to PDL1 also blocked by CD80 binding in cis?
We have now provided new data in EV1 to address this point. The data show that even in the presence of high affinity clinical antibodies, the staining of PD-L1 is inhibited by CD80 resulting in an ~4 fold shift in EC 50. It is notable that at the highest concentrations of antibody the stain reaches maximal levels comparable with PD-L1 in isolation.

Figure 7: the authors report that abatacept does not alter PD-1 binding; it can be expected that this is also the case for belatacept but if would be interesting to address this. *We now show this data in EV8. The referee is indeed correct that belatacept does not enhance PD-1-Ig binding any more than abatacept. Indeed the data are remarkably similar to abatacept. We believe this highlights an important point, which is that simple binding of CTLA-4 molecules to CD80-PD-L1 is not responsible for release of PD-L1.*

Ligand levels: The authors should show fluorophore expressing and ligand expression (e.g. GFP expression and CD80 staining in Fig. EV4) *We have now added additional data to EV6, which shows the correlation between antibody stains and GFP or mCherry expression as part of our experiments to more accurately quantify expression levels of these proteins.*

If CD80 and CD86 are coexpressed is CD80 preferentially removed from the APC via CTLA-4 mediated transendocytosis? *This is the case in our experience, and we are presently putting together a separate manuscript that studies this issue in detail.*

Does CD80 has to be expressed in excess of PD-L1 to prevent PD-1-Ig binding or is this observed when both ligands are expressed at equimolar concentrations? *We have now provided a new figure (figure 5), which shows that PD-1 binding to PD-L1 can be extinguished at a 1:1 ratio.*

Royal Free & University College Medical School Rowland Hill Street, London NW3 2PF
Tel: +44 (0)20 7794 0500 x 22469. Email: d.sansom@ucl.ac.uk
http://www.ucl.ac.uk/medicalschool/infection-immunity/

Referee #2:

Kennedy et al. show in in vitro experiments 1) CTLA4 on donor cells specially bind to CD80 in the heterodimer of PD-L1:CD80; 2) CTLA4 transendocytosis selectively remove CD80 but not PD-L1; 3) Effective removal of CD80 via transendocytosis requires CTLA4 cytoplasmic domain; 4) Transendocytosis of CD80 by CTLA4 but not blocking soluble CTLA4 Ig effectively liberates PD-L1. The authors conclude that efficient CD80 depletion by transendocytosis but not simply CTLA-4 binding to CD80 is required to effectively liberate PD-L1 that can engage PD-1. These observations are interesting, and the manuscript is clearly and concisely written.

There is a minor concern need to be addressed. Most of the recent publications on cis PD-L1/CD80 interactions are based on in vitro transfection experiments with PD-L1 and CD80. Those reports and the current report all elegantly show that there is only cis PD-L1/CD80 interactions. However, previous experiments (Park et al: Blood 2010; Yi et al J. Immunol. 2011, Deng et al 2015; Ni et al: JCI 2017) clearly indicate the existence of trans PD-L1/CD80 interactions in vivo. In vitro interactions cannot be used to exclude complex in vivo interactions. This notion should be made in the discussion.
We thank the referee for their comments and have now added some further references in the introduction and discussion that provide more balance in this area.

Referee #3:

Sansom review

Adding to several recent publications on the cis CD80/PD-L1 interaction, the authors show that transendocytosis by CTLA4 of a CD80/PD-L1 heterodimer removes CD80 but not PD-L1 from the cell surface, allowing the PD-L1 to interact with PD-1. They propose that CD80 can operate as a switch and by preventing PD-L1 from interacting with PD-1 and by its higher avidity binding to CTLA4 also protect CD86 from transendocytosis by CTLA4, the CD80 can promote enhanced T cell responses by inhibiting both CTLA-4 and PD-1 pathways. The work is well done and deepens our understanding of a wicked complicated set of interactions but is with transfectants and not natural cells. No direct experiments on immune function are performed.
We thank the referee for their helpful comments and critical reading of our manuscript. We have provided a point-by-point response below.

Major:
1. Zhao et al propose that the CD80/PD-L1 interaction, by reducing CD80 to a monomeric state, reduces the avidity for CTLA-4. Do a dose titration of CTLA4-Ig on CHO-CD80 and CHO-CD80/PD-L1 cells and determine the EC50 of CTLA4 binding and see if the avidity is altered. This will have to be done on CHO cell transfectants since endogenous CD80 and CD86 would complicate the analysis in DG-75 cells.
We have now performed these experiments and provide an EC50 for abatacept (figure 8) and the higher affinity belatacept (EV8). In neither case do we observe significant shifts in EC50, strongly suggesting that

2. Specify concentrations on x-axis of Figure 7 (these are DG-75 cells). Calculate EC50 of CTLA4-Ig binding to CD80 vs CD80/PD-L1 from Fig 7A. *We have now shown concentrations of Abatacept in the figures along with the EC50 as above.*

3. What is the molar ratio of PD-L1 to CD80 in the transfectants? Which is in excess? This cannot be determined by FACS but could be done by mass spectroscopy. *We have now provided a new figure (figure 5), which shows that PD-1 binding to PD-L1 can be extinguished at a CD80-PD-L1 ratio of 1:1. In this figure we show the results from cells expressing different molar ratios quantified using calibration beads to quantify number of molecules.*

4. How much CD80, CD86, PD-L1 does the Burkitts lymphoma cell line DG-75 express (generally Burkitts lines express)? Show in a supplemental figure. Is there binding to GFP-less native CD80 or CD86 which may complicate interpretation? *We have now provided information in the methods to make it clear we have deleted the endogenous CD80 and CD86 molecules before replacing them with tagged versions used in the study.*

Royal Free & University College Medical School Rowland Hill Street, London NW3 2PF
Tel: +44 (0)20 7794 0500 x 22469. Email: d.sansom@ucl.ac.uk
http://www.ucl.ac.uk/medicalschool/infection-immunity/

Minor:
5. In introduction, distinguish characteristics of transendocytosis and trogocytosis. How are they distinguished? *We have now provided further detail in the introduction.*

6. "acquisition by the CTLA-4+ recipient population using a well-established assay " describe cells used in assay, particularly CTLA-4 recipient cells.
7. Move Figure EV2 to main text so assay is clear to reader at beginning. *We tried to move EV2 but could not satisfactorily incorporate it into a main figure. We have now tried to alert readers to the assay design further by reference to EV2 in the legend to fig.2.*

8. The labeling of the figures and descriptions in the figure legends is in many places inadequate to clearly understand what has been done and how. There are many examples but to specify a few: *Apologies we have now been through and corrected these issues in the legends and methods to make this clearer.*

9. In 1D, specify ab used for IP and for WB *Done*

10. The labeling of Figure 2 does not provide enough information. The donor and recipient cells for each panel (and their CTV labeling) should be clearly indicated in the figure. *We have tried to improve the labelling, CTV labelling is clearly shown, and we have added recipient and donor labels to 2A to help orient the reader, together with Fig EV2 to clearly explain the panels.*

11. In 2A, are the upper left and right panels duplicate assays? *No. the top left panel is a CD80 donor cell, and the top right is CD80-PD-L1. In both cases there is no CTLA-4 in this system which is shown in the two panels below.*

12. Throughout all figures, where "ligand" is written, specify molecule. Do not use "no ligand" *We specified ligand throughout and removed the use of "no ligand".*

13. Methods says "ratios of donor: recipient cells and incubation times indicated in the figure legends." But figures often do not provide these numbers. Recipient cells need to be specified in figures. *We have now specified the recipient and donor cell quadrants in the first TE FACS plot (Fig. 2A), which together with the cartoon depicting the TE assay in Fig. EV2 should hopefully orient the reader.*

14. In 3C and similar, the left panel y-axis is labeled "ligand remaining". Specify which ligand in each case; presumably CD80? *We have labelled these more clearly*

15. In methods, give equation of how ligand remaining was calculated in Figure 2D and similar figures. *We have added this.*

16. In 2A, choice of overnight incubation is a poor one since the amount of detectable transferred ligand is low (presumably because of degradation). Suggest using a time point where degradation is less dominant. *We agree there are some limitations in showing the longer time point first, however, we wanted to emphasise the extent of removal of ligand from the donor cell. Since a time course showing earlier time points is available in 2C below, we feel the reader should be able to gauge these various nuances from the data.*

17. In 2B, the question is whether the PD-L1mCherry is being transendocytosed by the CTLA4 cell. Say the red quadrant is loss of PD-L1. There does not seem to be a shift in the CTV positive population but the MFI intensity of the total cell population of the lower (CTV negative) population is clearly shifted to the right. Why does this shift not mean that PD-L1 is being broadly taken up by the recipient cells, maybe on the cell surface and not internalized? *We agree there is a small shift of the recipient cells to the right. However, we don't feel this signifies high levels of uptake. In our experience of transendocytosis assays measuring ligand uptake is the least reliable- as transferred material is often degraded- as is the case for CD80 or CD86. Whenever labelled cells are in contact there is always some transfer by trogocytosis, which occurs at the point at which the cells are separated- resulting in small fluorescent signals. In contrast, removal of ligand from the donor cell in a time dependent manner is a much more rigorous measure and it is clear from this data that the amount of PD-L1 lost from the donor cells must be small. Comparing the graphs in 2E and 2F gives some sense of this.*

18. Calculate statistical significance between lines in 2D (CD80 vs CD80+PD-L1) No significant difference was observed between these 2 lines, this has now been stated in the main text and added to fig 2D.

Royal Free & University College Medical School Rowland Hill Street, London NW3 2PF
Tel: +44 (0)20 7794 0500 x 22469. Email: d.sansom@ucl.ac.uk
http://www.ucl.ac.uk/medicalschool/infection-immunity/

19. "CTLA-4 cytoplasmic domain was required for effective time-dependent depletion of CD80 and CD86 " Soften this statement (required is too extreme). In general, this data is discussed appropriately but the titles are extreme. There is some trogocytosis/transendocytosis by Cytdel (11% vs control of 2%) *We have toned down the titles and text where appropriate.*
20. "The reason for the differences between these observations currently remain unclear but may relate to differences in how transendocytosis was measured. "
Describe differences between your method and Zhao's. *We have added this discussion*

21. In the discussion, also note that in natural cells, PD-L1 is associated with CMTM6 and this is not replicated in transfectants. *We have added some discussion on this point.*
22. Specify GFP used in methods and linker length. *done*
23. Specify CTLA-4 del36 in methods. done

References

Sugiura, D., Maruhashi, T., Okazaki, I.M., Shimizu, K., Maeda, T.K., Takemoto, T., and Okazaki, T. (2019). Restriction of PD-1 function by cis-PD-L1/CD80 interactions is required for optimal T cell responses. Science *364*, 558-566.

Tekguc, M., Wing, J.B., Osaki, M., Long, J., and Sakaguchi, S. (2021). Treg-expressed CTLA-4 depletes CD80/CD86 by trogocytosis, releasing free PD-L1 on antigen-presenting cells. Proceedings of the National Academy of Sciences of the United States of America *118*.

Zhao, Y., Lee, C.K., Lin, C.H., Gassen, R.B., Xu, X., Huang, Z., Xiao, C., Bonorino, C., Lu, L.F., Bui, J.D., and Hui, E. (2019). PD-L1:CD80 Cis-Heterodimer Triggers the Co-stimulatory Receptor CD28 While Repressing the Inhibitory PD-1 and CTLA-4 Pathways. Immunity *51*, 1059-1073 e1059.

Royal Free & University College Medical School Rowland Hill Street, London NW3 2PF
Tel: +44 (0)20 7794 0500 x 22469. Email: d.sansom@ucl.ac.uk
http://www.ucl.ac.uk/medicalschool/infection-immunity/

1st Revision - Editorial Decision 28th Nov 2022

Dear David,

Thank you for submitting your revised manuscript to The EMBO Journal. Your study has now been seen by referees # 1 & 3. As you can see from the comments below, both referees appreciate the introduced changes. Referee #3 has a few remaining points regarding the text that I would like to ask you to address in a final revision.

When you submit your revised version, please also address the following editorial points:

- Please upload a "clean" manuscript version with no text markings.

- You are missing a Data Availability section. This is the place to enter accession numbers etc. If no data is generated that needs to be deposited in a databasethen please state: Data Availability: This study includes no data deposited in external repositories.

- You have 8 EV figures but can only have 5. Can you combine some of them for example EV2, 3 & EV7?

- Please make sure that EV figures panels are called out - use EV1a... nomenclature.

- COI needs to be renamed as " Disclosure and competing interests statement"

- Please check the size of the synopsis figure should be [550 wide by 200-400]

- Our publisher has also done their pre-publication check on your manuscript. When you log into the manuscript submission system you will see the file "Data Edited Manuscript file". Take a look at the word file and the comments regarding the figure legends and respond to the issues.

That should be all!

Congratulations on an interesting study

With best wishes

Karin

Karin Dumstrei, PhD
Senior Editor
The EMBO Journal

Instructions for preparing your revised manuscript:

Guide For Authors: https://www.embopress.org/page/journal/14602075/authorguide

Use the link below to submit your revision:

https://emboj.msubmit.net/cgi-bin/main.plex

Referee #1:

The authors have undergone considerable effort to revise their work. The manuscript has clearly improved.

Referee #3:

The authors have done additional experimental work which strengthens their story and addresses my concerns. This includes an experiment showing a biological effect.
I would suggest the authors:
Point 12, Reviewer 3 has not been adequately addressed. Ligand is still used in Figure labels without specifying what the molecule is. In Figure 6 labeling, explicitly say what molecule is meant. Do not use Ligand-GFP or Ligand-PD-L1 colocalization.

If appropriate, add more description to Figure legend.

The experimental setup in Figure EV1C is unclear. The legend says: "Down regulation of CD28 following engagement by CD80 or CD86 in the presence or absence of PD-L1 is shown as a measure of CD28 interaction with ligands." Please provide enough information to understand clearly. There must be a CD28 T cell here?

Fig 7 legend. "WT CTLA-4+ Jurkat T- cells (Jreg) or control cells". Specify control cells; untransfected Jurkat?

All editorial and formatting issues were resolved by the authors.

Dear David,

Thank you for submitting your revised manuscript. I have now looked at everything and all looks good. I am therefore very pleased to accept the MS for publication here.

Congratulations on a nice study!

best Karin

Karin Dumstrei, PhD
Senior Editor
The EMBO Journal

Please note that it is EMBO Journal policy for the transcript of the editorial process (containing referee reports and your response letter) to be published as an online supplement to each paper. If you do NOT want this, you will need to inform the Editorial Office via email immediately. More information is available here:
https://www.embopress.org/page/journal/14602075/authorguide#transparentprocess

Your manuscript will be processed for publication in the journal by EMBO Press. Manuscripts in the PDF and electronic editions of The EMBO Journal will be copy edited, and you will be provided with page proofs prior to publication. Please note that supplementary information is not included in the proofs.

You will be contacted by Wiley Author Services to complete licensing and payment information. The required 'Page Charges Authorization Form' is available here: https://www.embopress.org/pb-assets/embo-site/tej_apc.pdf - please download and complete the form and return to embopressproduction@wiley.com

EMBO Press participates in many Publish and Read agreements that allow authors to publish Open Access with reduced/no publication charges. Check your eligibility: https://authorservices.wiley.com/author-resources/Journal-Authors/open-access/affiliation-policies-payments/index.html

Should you be planning a Press Release on your article, please get in contact with embojournal@wiley.com as early as possible, in order to coordinate publication and release dates.

If you have any questions, please do not hesitate to call or email the Editorial Office. Thank you for your contribution to The EMBO Journal.

** Click here to be directed to your login page: https://emboj.msubmit.net

EMBO Press Author Checklist

Corresponding Author Name: David M Sansom
Journal Submitted to: The EMBO Journal
Manuscript Number: EMBOJ-2022-111556R

USEFUL LINKS FOR COMPLETING THIS FORM
The EMBO Journal - Author Guidelines
EMBO Reports - Author Guidelines
Molecular Systems Biology - Author Guidelines
EMBO Molecular Medicine - Author Guidelines

Reporting Checklist for Life Science Articles (updated January
This checklist is adapted from Materials Design Analysis Reporting (MDAR) Checklist for Authors. MDAR establishes a minimum set of requirements in transparent reporting in the life sciences (see Statement of Task: 10.31222/osf.io/9sm4x). Please follow the journal's guidelines in preparing your
Please note that a copy of this checklist will be published alongside your article.

Abridged guidelines for figures
1. Data
The data shown in figures should satisfy the following conditions:
- the data were obtained and processed according to the field's best practice and are presented to reflect the results of the experiments in an accurate and unbiased manner.
- ideally, figure panels should include only measurements that are directly comparable to each other and obtained with the same assay.
- plots include clearly labeled error bars for independent experiments and sample sizes. Unless justified, error bars should not be shown for technical
- if n<5, the individual data points from each experiment should be plotted. Any statistical test employed should be justified.
- Source Data should be included to report the data underlying figures according to the guidelines set out in the authorship guidelines on Data

2. Captions
Each figure caption should contain the following information, for each panel where they are relevant:
- a specification of the experimental system investigated (eg cell line, species name).
- the assay(s) and method(s) used to carry out the reported observations and measurements.
- an explicit mention of the biological and chemical entity(ies) that are being measured.
- an explicit mention of the biological and chemical entity(ies) that are altered/varied/perturbed in a controlled manner.
- the exact sample size (n) for each experimental group/condition, given as a number, not a range;
- a description of the sample collection allowing the reader to understand whether the samples represent technical or biological replicates (including how many animals, litters, cultures, etc.).
- a statement of how many times the experiment shown was independently replicated in the laboratory.
- definitions of statistical methods and measures:
  - common tests, such as t-test (please specify whether paired vs. unpaired), simple χ2 tests, Wilcoxon and Mann-Whitney tests, can be unambiguously identified by name only, but more complex techniques should be described in the methods section;
  - are tests one-sided or two-sided?
  - are there adjustments for multiple comparisons?
  - exact statistical test results, e.g., P values = x but not P values < x;
  - definition of 'center values' as median or average;
  - definition of error bars as s.d. or s.e.m.

Please complete ALL of the questions below.
Select "Not Applicable" only when the requested information is not relevant for your study.

Materials

Newly Created Materials	Information included in the manuscript?	In which section is the information available? (Reagents and Tools Table, Materials and Methods, Figures, Data Availability Section)
New materials and reagents need to be available; do any restrictions apply?	Yes	

Antibodies	Information included in the manuscript?	In which section is the information available? (Reagents and Tools Table, Materials and Methods, Figures, Data Availability Section)
For **antibodies** provide the following information:<br>- Commercial antibodies: RRID (if possible) or supplier name, catalogue number and or/clone number<br>- Non-commercial: RRID or citation	Yes	Materials and Methods

DNA and RNA sequences	Information included in the manuscript?	In which section is the information available? (Reagents and Tools Table, Materials and Methods, Figures, Data Availability Section)
**Short novel DNA or RNA including primers, probes**: provide the sequences.	Yes	Materials and Methods

Cell materials	Information included in the manuscript?	In which section is the information available? (Reagents and Tools Table, Materials and Methods, Figures, Data Availability Section)
**Cell lines:** Provide species information, strain. Provide accession number in repository **OR** supplier name, catalog number, clone number, and/**OR** RRID.	Yes	Materials and methods
**Primary cultures:** Provide species, strain, sex of origin, genetic modification status.	Not Applicable	
Report if the cell lines were recently **authenticated** (e.g., by STR profiling) and tested for mycoplasma contamination.	Yes	

Experimental animals	Information included in the manuscript?	In which section is the information available? (Reagents and Tools Table, Materials and Methods, Figures, Data Availability Section)
**Laboratory animals or Model organisms:** Provide species, strain, sex, age, genetic modification status. Provide accession number in repository **OR** supplier name, catalog number, clone number, **OR** RRID.	Not Applicable	
**Animal observed in or captured from the field:** Provide species, sex, and age where possible.	Not Applicable	
Please detail **housing and husbandry conditions**.	Not Applicable	

Plants and microbes	Information included in the manuscript?	In which section is the information available? (Reagents and Tools Table, Materials and Methods, Figures, Data Availability Section)
**Plants:** provide species and strain, ecotype and cultivar where relevant, unique accession number if available, and source (including location for collected wild specimens).	Not Applicable	
**Microbes:** provide species and strain, unique accession number if available, and source.	Not Applicable	

Human research participants	Information included in the manuscript?	In which section is the information available? (Reagents and Tools Table, Materials and Methods, Figures, Data Availability Section)
If collected and within the bounds of privacy constraints report on age, sex and gender or ethnicity for all study participants.	Not Applicable	

Core facilities	Information included in the manuscript?	In which section is the information available? (Reagents and Tools Table, Materials and Methods, Figures, Data Availability Section)
If your work benefited from core facilities, was their service mentioned in the acknowledgments section?	Not Applicable	

Design

Study protocol	Information included in the manuscript?	In which section is the information available? (Reagents and Tools Table, Materials and Methods, Figures, Data Availability Section)
If study protocol has been **pre-registered, provide DOI in the manuscript**. For clinical trials, provide the trial registration number **OR** cite DOI.	Not Applicable	
Report the **clinical trial registration number** (at ClinicalTrials.gov or equivalent), where applicable.	Not Applicable	

Laboratory protocol	Information included in the manuscript?	In which section is the information available? (Reagents and Tools Table, Materials and Methods, Figures, Data Availability Section)
Provide DOI OR other citation details if **external detailed step-by-step protocols** are available.	Not Applicable	

Experimental study design and statistics	Information included in the manuscript?	In which section is the information available? (Reagents and Tools Table, Materials and Methods, Figures, Data Availability Section)
Include a statement about **sample size** estimate even if no statistical methods were used.	Yes	Materials & Methods, Figure Legends
Were any steps taken to minimize the effects of subjective bias when allocating animals/samples to treatment (e.g. **randomization procedure**)? If yes, have they been described?	Not Applicable	
Include a statement about **blinding** even if no blinding was done.	Yes	Materials and Methods
Describe **inclusion/exclusion criteria** if samples or animals were excluded from the analysis. Were the criteria pre-established?<br><br>If sample or data points were omitted from analysis, report if this was due to attrition or intentional exclusion and provide justification.	Not Applicable	
For every figure, are **statistical tests** justified as appropriate? Do the data meet the assumptions of the tests (e.g., normal distribution)? Describe any methods used to assess it. Is there an estimate of variation within each group of data? Is the variance similar between the groups that are being statistically compared?	Yes	

Sample definition and in-laboratory replication	Information included in the manuscript?	In which section is the information available? (Reagents and Tools Table, Materials and Methods, Figures, Data Availability Section)
In the figure legends: state number of times the experiment was **replicated** in laboratory.	Yes	Figure legends
In the figure legends: define whether data describe **technical or biological replicates**.	Yes	Figure legends

Ethics

Ethics	Information included in the manuscript?	In which section is the information available? (Reagents and Tools Table, Materials and Methods, Figures, Data Availability Section)
Studies involving **human participants**: State details of **authority granting ethics approval** (IRB or equivalent committee(s), provide reference number for approval.	Not Applicable	
Studies involving **human participants**: Include a statement confirming that **informed consent** was obtained from all subjects and that the experiments conformed to the principles set out in the WMA Declaration of Helsinki and the Department of Health and Human Services Belmont Report.	Not Applicable	
Studies involving **human participants:** For publication of **patient photos**, include a statement confirming that consent to publish was obtained.	Not Applicable	
Studies involving experimental **animals**: State details of **authority granting ethics approva**l (IRB or equivalent committee(s), provide reference number for approval. Include a statement of compliance with ethical regulations.	Not Applicable	
Studies involving **specimen and field samples:** State if relevant **permits** obtained, provide details of authority approving study; if none were required, explain why.	Not Applicable	

Dual Use Research of Concern (DURC)	Information included in the manuscript?	In which section is the information available? (Reagents and Tools Table, Materials and Methods, Figures, Data Availability Section)
Could your study fall under dual use research restrictions? Please check biosecurity documents and list of **select agents and toxins** (CDC): https://www.selectagents.gov/sat/list.htm	Not Applicable	
If you used a select agent, is the security level of the lab appropriate and reported in the manuscript?	Not Applicable	
If a study is subject to dual use research of concern regulations, is the name of the **authority granting approval and reference number** for the regulatory approval provided in the manuscript?	Not Applicable	

Reporting

The MDAR framework recommends adoption of discipline-specific guidelines, established and endorsed through community initiatives. Journals have their own policy about requiring specific guidelines and recommendations to complement MDAR.

Adherence to community standards	Information included in the manuscript?	In which section is the information available? (Reagents and Tools Table, Materials and Methods, Figures, Data Availability Section)
State if relevant guidelines or checklists (e.g., **ICMJE, MIBBI, ARRIVE, PRISMA**) have been followed or provided.	Not Applicable	
For **tumor marker prognostic studies,** we recommend that you follow the **REMARK** reporting guidelines (see link list at top right). See author guidelines, under 'Reporting Guidelines'. Please confirm you have followed these guidelines.	Not Applicable	
For **phase II and III randomized controlled trials**, please refer to the **CONSORT** flow diagram (see link list at top right) and submit the CONSORT checklist (see link list at top right) with your submission. See author guidelines, under 'Reporting Guidelines'. Please confirm you have submitted this list.	Not Applicable	

Data Availability

Data availability	Information included in the manuscript?	In which section is the information available? (Reagents and Tools Table, Materials and Methods, Figures, Data Availability Section)
Have **primary datasets** been deposited according to the journal's guidelines (see 'Data Deposition' section) and the respective accession numbers provided in the Data Availability Section?	Not Applicable	
Were **human clinical and genomic datasets** deposited in a public access-controlled repository in accordance to ethical obligations to the patients and to the applicable consent agreement?	Not Applicable	
Are **computational models** that are central and integral to a study available without restrictions in a machine-readable form? Were the relevant accession numbers or links provided?	Not Applicable	
If publicly available data were reused, provide the respective **data citations in the reference list**.	Not Applicable	
